# In vivo and in vitro reconstitution of unique key steps in cystobactamid antibiotic biosynthesis

**DOI:** 10.1038/s41467-021-21848-3

**Published:** 2021-03-16

**Authors:** Sebastian Groß, Bastien Schnell, Patrick A. Haack, David Auerbach, Rolf Müller

**Affiliations:** 1 Helmholtz Institute for Pharmaceutical Research Saarland (HIPS), Helmholtz Centre for Infection Research, Saarland University, Campus E8.1, 66123 Saarbrücken, Germany; 2 Department of Pharmacy, Saarland University, 66123 Saarbrücken, Germany; 3 DZIF - German Centre for Infection Research, Partnersite Hannover-Braunschweig, 38124 Braunschweig, Germany

**Keywords:** Expression systems, Multienzyme complexes, Natural products, Antibiotics

## Abstract

Cystobactamids are myxobacteria-derived topoisomerase inhibitors with potent anti-Gram-negative activity. They are formed by a non-ribosomal peptide synthetase (NRPS) and consist of tailored *para*-aminobenzoic acids, connected by a unique α-methoxy-l-isoasparagine or a β-methoxy-l-asparagine linker moiety. We describe the heterologous expression of the cystobactamid biosynthetic gene cluster (BGC) in *Myxococcus xanthus*. Targeted gene deletions produce several unnatural cystobactamids. Using in vitro experiments, we reconstitute the key biosynthetic steps of linker formation and shuttling via CysB to the NRPS. The biosynthetic logic involves a previously uncharacterized bifunctional domain found in the stand-alone NRPS module CysH, albicidin biosynthesis and numerous BGCs of unknown natural products. This domain performs either an aminomutase (AM) or an amide dehydratase (DH) type of reaction, depending on the activity of CysJ which hydroxylates CysH-bound l-asparagine. Furthermore, CysQ *O*-methylates hydroxyl-l-(iso)asparagine only in the presence of the AMDH domain. Taken together, these findings provide direct evidence for unique steps in cystobactamid biosynthesis.

## Abstract (web summary)

The biosynthetic pathway leading to the production of cystobactamids, a group of myxobacteria-derived topoisomerase inhibitors with potent anti-Gram-negative activity, remains unclear. Here, the authors report in vivo and in vitro evidence for unique steps in cystobactamid biosynthesis.

## Introduction

**Fig. 1:**
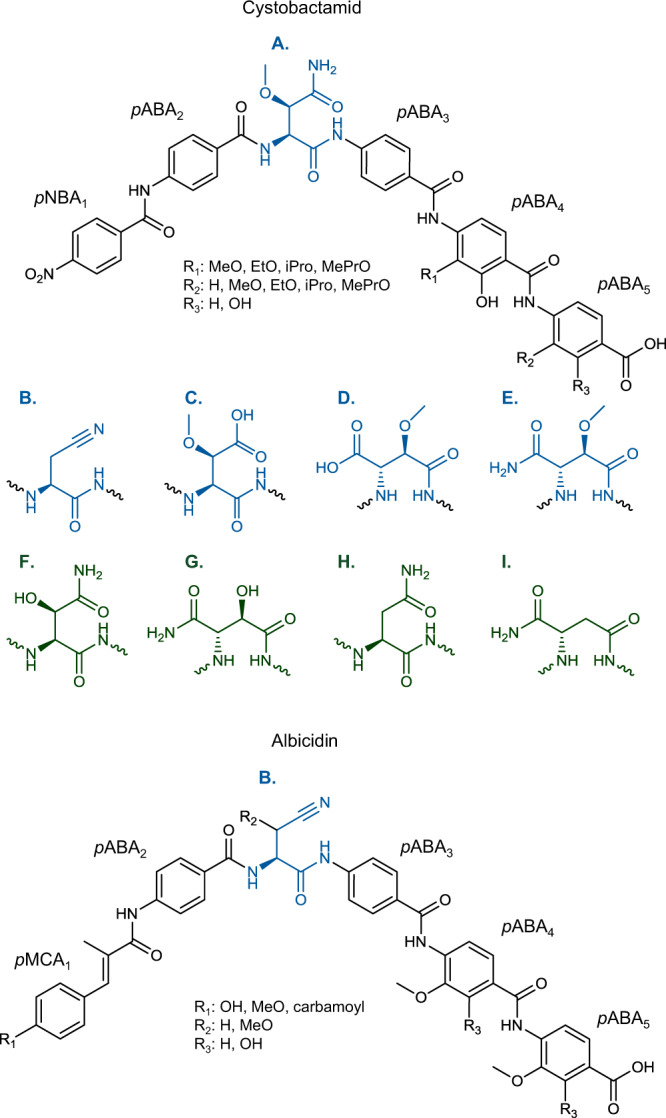
Structural variations among native and unnatural cystobactamids and structure comparison with albicidin. *para*-Nitrobenzoic acid (*p*NBA) and *para*-aminobenzoic acid (*p*ABA) with possible substitutions (*R*_1_, *R*_2_, *R*_3_) are shown in black. Different linker moieties of natural cystobactamids (shown in blue; linker (**A**)–(**E**)) and unnatural cystobactamids (shown in green; linker (**F**)–(**I**); Table 1): (**A**) β-methoxy-l-asparagine, (**B**) β-cyano-l-alanine, (**C**) β-methoxy-l-aspartate, (**D**) α-methoxy-l-isoaspartate, (**E**) α-methoxy-l-isoasparagine, (**F**) β-hydroxy-l-asparagine, (**G**) α-hydroxy-l-isoasparagine, (**H**) l-asparagine, (**I**) l-isoasparagine. The scheme was adapted from Hüttel et al.^2^. The stereochemistry of the linker moiety is based on the assignment by Planke et al.^44^. Albicidin carries an N-terminal *para*-methylcoumaric acid (*p*MCA_1_), two *p*ABAs, two substituted *p*ABAs, and a (possibly modified) β-cyano-l-alanine (**B**) or β-methoxy-l-asparagine (**A**) linker. Different possible substitutions (*R*_1_, *R*_2_, *R*_3_) are given.

The cystobactamids are a family of non-ribosomally synthesized peptide antibiotics produced by different myxobacteria such as *Cystobacter velatus* Cbv34 and *Myxococcus fulvus* SBMx122^1,2^. They target the bacterial topoisomerase IIA, but no cross-resistance was observed to clinically used gyrase inhibitors of the fluoroquinolone family, which share the same target^1^. The major derivatives in Cbv34 extracts are cystobactamid (Cys)919-1, Cys919-2, and Cys507^1^. The prototypical structures (shown in Fig. 1) feature one *para*-nitrobenzoic acid (*p*NBA), four *para*-aminobenzoic acids (*p*ABA), and an unusual l-isoasparagine or l-asparagine linker moiety (*p*NBA_1_-*p*ABA_2_-(iso)Asn-*p*ABA_3_-*p*ABA_4_-*p*ABA_5_).

l-isoasparagine or l-asparagine are usually α- or β-methoxylated, respectively. *p*ABA_4_ and *p*ABA_5_ are commonly isopropoxylated in position 2 and *p*ABA_5_ are additionally hydroxylated in position 3^1^. Cys507, however, only consists of the three (tailored) *C*-terminal *p*ABA_4-6_ moieties. In total, thirteen native cystobactamid derivatives were described, which differ in the structure of their linker moiety and the tailoring pattern of *p*ABA_4_ and *p*ABA_5_^2^. Interestingly, antibacterial activity is nearly limited to derivatives that belong to cystobactamid series 2 harboring an l-asparagine linker. Particularly interesting is the native derivative Cys861-2 showing low micromolar activity against *Acinetobacter baumanii*, *Pseudomonas aeruginosa*, *Escherichia coli*, and other pathogens^2^ that are classified with high- to critical-priority by the WHO^3^. Most of the cystobactamids with other linker moieties are inactive or show only weak antibacterial activity. Furthermore, total syntheses for several native cystobactamids and synthetic derivatives with improved antibacterial activity or metabolic stability were described^2,4–7^. Notably, cystobactamids show structural similarity with albicidins (shown in Fig. 1), aPKS/NRPS product class isolated from *Xanthomonas albilineans*, which also shows antibacterial activity^8–10^. However, significant structural differences between both compound classes are found in the N-terminal parts and the linker moieties. A methylated *para*-coumaric acid moiety (*p*MCA_1_) typically forms the N-terminal part in albicidins, which can be further tailored, e.g., with a carbamoyl group^10^, whereas native cystobactamids are restricted to *p*NBA_1_. The linker moieties arising in albicidins are β-cyano-l-alanine, which was also observed in Cys871^2^, and l-asparagine, both optionally methoxylated^10^, but no l-isoasparagine linker was described so far.

Non-ribosomal peptide synthetases (NRPSs) are large enzyme complexes with a multimodular architecture, in which each module is subdivided into independent domains, each usually catalyzing a single reaction. Adenylation (A) domains activate amino acids using ATP, thiolation (T) domains tether the activated amino acid or the growing peptide, and condensation (C) domains catalyze peptide bond formation^11–13^. Tailoring of the product happens either after product release or on the assembly line. In the latter case, both in trans tailoring by independent enzymes and in cis modifications by tailoring domains, such as epimerization (E), heterocyclization (Cy), or methyltransferase (MT) domains, can occur^14^. NRPS biosynthesis is not limited to proteinogenic amino acids, thus allowing a great variability of chemical scaffolds^15^, such as shown for the daptomycin, a natural product featuring the unusual amino acid l-kynurenine^16^. Notably, NRPSs typically follow two important rules: first, the collinearity rule states that each module catalyzes the incorporation of a single building block into a growing peptide chain^17^. Second, the processivity rule states that the biosynthesis starts from the first module and proceeds sequentially to the next modules.

A model for the biosynthesis of cystobactamids was proposed by Baumann and coworkers based on in silico analyses of the BGC and feeding experiments with isotope-labeled amino acids^1^. In this model, modules 1, 2, and 4–6 on the NRPS enzymes CysK and CysG incorporate the two N-terminal and three C-terminal *p*ABA units, *p*ABA_1_-_2_ and *p*ABA_4_-_6_, respectively. However, the A_3_ domain of module 3 in CysK was assumed inactive, because the core motif A10^18^ lacks the catalytically essential lysine residue^19,20^. Therefore, the authors proposed that the T_3_ domain in CysK is primed in trans by the stand-alone NRPS CysH with the help of the putative shuttling protein CysB. The stand-alone NRPS module CysH was proposed to activate l-asparagine, which is either used directly to prime T_3_ or isomerized by an unusual ammonia/amine-ligase-like domain in CysH. Finally, Baumann and coworkers assumed that the linear hexapeptide is released from the assembly line by the TE of CysG being further modified by various tailoring enzymes afterward. By comparison, in the β-cyano-l-alanine linker biosynthesis in albicidin, Cociancich and colleagues proposed activation of l-asparagine by the stand-alone NRPS module 2* (AlbIV) and phosphorylation of the side chain amide oxygen in cis by a domain harboring an ATP-binding motif. They postulated that subsequent dephosphorylation would lead to formal elimination of water and formation of β-cyano-l-alanine (shown in Supplementary Fig. 9b). However, none of the reaction steps were experimentally proven in either of the previous publications.

Native myxobacterial producing strains are often difficult to cultivate and genetically manipulate, making the study of their natural products biosynthesis challenging^21^. Heterologous expression of biosynthetic gene clusters (BGCs) can circumvent these issues, but identification of proper host strains and subsequent cloning of the complex clusters remain significant bottlenecks. Nevertheless, a number of myxobacterial BGCs have been heterologously expressed in *Myxococcus xanthus* DK1622^21,22^. In addition to heterologous expression systems, overexpression of individual proteins and their biochemical analysis in vitro can be used to gain further insights into the biosynthesis of natural products.

Herein, we describe the design, assembly, and heterologous expression of a modified cystobactamid BGC in *M. xanthus* DK1622. We identify 13 previously uncharacterized natural cystobactamids upon expression of all biosynthetic genes and nine unnatural derivatives after targeted gene deletions. Targeted gene deletions in combination with in vitro investigation of the enzyme activities allow explaining the unique biosynthesis steps of the α-methoxy-l-isoasparagine linker and its shuttling to the assembly line. This building block is synthesized by the independent NRPS module CysH and the bifunctional in cis tailoring aminomutase/amide dehydratase (AMDH) domain, working in tandem with the oxygenase CysJ and the *O*-methyltransferase CysQ. Finally, this moiety is transferred onto module 3 of CysK by the shuttling protein CysB. Furthermore, we confirm that the biosynthesis of the *N*-terminally truncated derivative Cys507 starts from the middle of the assembly line, bending the processivity rule. With these results, we are able to decipher most of the obscure and unique steps of cystobactamid linker biosynthesis. We provide a heterologous production platform for topoisomerase inhibitors and discover unexpected plasticity of NRPS biosynthesis.

## Results

### Heterologous production of cystobactamids in *M. xanthus* DK1622

A modified BGC together with a cloning and expression vector system were designed in silico for the heterologous production of cystobactamids in *M. xanthus* DK1622 (described in Supplementary Method 2 and 3; Supplementary Figs. 1–3 and Supplementary Tables 1–5). The revised template sequence of the BGC originated from *C. velatus* Cbv34 (see Supplementary Method 1), including the 25 biosynthetic genes *cysA-T* and *Orf1-5* as previously described^1^. The modified BGC was chemically synthesized in fragments, because of the size, GC content, and repetitive sequence segments in the cluster. Assembly of the cluster fragments was done by a combination of in vivo transformation-associated recombination (TAR) cloning in yeast^23^ and a previously described in vitro three-step restriction/ligation cloning strategy^24^ using *Bsa*I (cloning steps are summarized in Supplementary Method 4, Supplementary Figs. 4 and 5 and Supplementary Table 6). The final expression construct pMYC20Cys_v2 was integrated into the *M. xanthus* DK1622 genome via the *Mx8* phage integrase.

UPLC-HRMS analysis and MS^2^ experiments confirmed the heterologous production of 13 unknown and 9 known cystobactamids (Table 1; see Supplementary Method 5; Supplementary Figs. 6–8 and 14; Supplementary Data 4). Notably, the 13 previously unidentified derivatives were also produced in native producer strains under the same cultivation conditions. The production titer of the major product Cys919-1 was 8.1 mg L^−1^ in the heterologous producer as compared to 3.6 mg L^−1^ in *Myxococcus fulvus* SBMx122. Formerly mentioned production yields in native producer strains were much lower with 60–100 µg compound isolated per 1 L culture^1,2^. However, difficulties in upscaling and compound loss during purification resulted in yields of isolated products similar to those originally reported. Subsequently, Red/ET recombineering in combination with *Bsa*I restriction and ligation was used to generate scarless gene deletions of *cysQ*, *cysJ*, the AMDH domain of *cysH*, *cysB*, and *cysR* independently. Using this strategy, we identified nine unnatural cystobactamid derivatives (plus one recently reported as Coralmycin D^25^) by heterologous expression of the manipulated constructs (Table 1; see Supplementary Information; Supplementary Figs. 6–7 and 15; Supplementary Data 4).

**Table 1:** Natural and unnatural cystobactamids heterologously produced by *M. xanthus* DK1622.

Construct	Product	Linker	*R*_1_	*R*_2_	*R*_3_
pMYC20 Cys_v2	Cys449^a^	–	*i*PrO	H	H
	Cys507^a^	–	*i*PrO	*i*PrO	H
	Cys861-1	E	*i*PrO	H	H
	Cys861-2^a^	A	*i*PrO	H	H
	Cys871^b^	B	*i*PrO	*i*PrO	H
	Cys877-1	E	EtO	MeO	H
	Cys877-2^a^	A	EtO	MeO	H
	Cys891-1a	E	EtO	EtO	H
	Cys891-1b	E	*i*PrO	MeO	H
	Cys891-2a	A	EtO	EtO	H
	Cys891-2^a^	A	*i*PrO	MeO	H
	Cys905-1a	E	*i*PrO	EtO	H
	Cys905-1b	E	EtO	*i*PrO	H
	Cys905-2a	A	EtO	*i*PrO	H
	Cys905-2^a^	A	*i*PrO	EtO	H
	**Cys919-1**^a^	**E**	***i*PrO**	***i*PrO**	**H**
	**Cys919-2**^a^	**A**	***i*PrO**	***i*PrO**	**H**
	Cys920-1^b^	C	*i*PrO	*i*PrO	H
	Cys920-2^b^	D	*i*PrO	*i*PrO	H
	Cys933-1a	E	*i*PrO	1-MePrO	H
	Cys933-1b	E	MePrO	*i*PrO	H
	Cys933-2a	A	*i*PrO	1-MePrO	H
	Cys933-2b	A	MePrO	*i*PrO	H
	Cys934-2^b^	D	iPro	1-MePrO	H
	Cys935-1	E	*i*PrO	*i*PrO	OH
	Cys935-2^a^	A	*i*PrO	*i*PrO	OH
pMYC20Cys_v2∆AMDH	Cys905-2c	F	*i*PrO	*i*PrO	H
pMYC20Cys_v4∆*cysQ*	Cys905-1c	G	*i*PrO	*i*PrO	H
	Cys905-2c	F	*i*PrO	*i*PrO	H
pMYC20Cys_v4∆*cysJ*	Cys889-1a	I	*i*PrO	*i*PrO	H
	Cys889-2a	H	*i*PrO	*i*PrO	H
	Cys871^a^	B	*i*PrO	*i*PrO	H
pMYC20Cys_v4∆*cysJ*∆AMDH	Cys889-2a	H	*i*PrO	*i*PrO	H
pMYC20Cys_v4∆*cysB*	Cys507^a^	–	*i*PrO	*i*PrO	H
pMYC20Cys_v4∆*cysR*	Cys889-1b^c^	E	*i*PrO	*i*PrO	H
	Cys889-2b^a,c^	A	*i*PrO	*i*PrO	H

Linker and *R*_1_, *R*_2_, *R*_3_ labeling was adapted from Fig. 1. pMYC20Cys_v2 includes all genes from the cystobactamid BGC described by Baumann et al.^1^. Derivatives **in bold** are major products in native producer strains and the heterologous producer. For deletion constructs, only major products, which are relevant for the elucidation of the linker biosynthesis, are shown.

^a^Heterologously produced derivatives that were described previously.

^b^Known derivatives that were not identified in the heterologous producer.

^c^Cys889-1b and Cys889-2b (reported as Coralmycin D)^25^ carry an N-terminal amine rather than a nitro-group.

### Biosynthesis of the linker moiety

β-cyano-l-alanine linkers were found both in albicidin and in minor cystobactamid derivatives (Fig. 1)^2,9^. Interestingly, we found high structural similarity between the unknown domain of the single-standing NRPS AlbIV, which was hypothesized to catalyze dehydration of l-asparagine^9^, with the 38 kDa domain found inserted in the stand-alone NRPS CysH (69% identity/83% similarity), which is involved in cystobactamid linker biosynthesis (Supplementary Fig. 9a)^1^. Despite the high structural similarity between these two unusual domains, completely different reaction mechanisms were proposed (Supplementary Fig. 9b, c). The major cystobactamid derivative harbors a modified l-isoasparagine linker and Baumann *et al*. proposed that the unusual domain catalyzes the isomerization of CysH-bound l-asparagine to l-isoasparagine^1^. Feeding of ^15^N_2_^13^C_4_-labeled l-asparagine during fermentation of native producer *C. velatus* Cbv34 confirmed full conservation of all carbons and nitrogens from l-asparagine in l-isoasparagine^1^ indicating an aminomutase-type reaction. Since cystobactamids contain β-cyano-l-alanine or l-isoasparagine linkers, we hypothesize that the unusual domain in CysH catalyzes either aminomutation (AM) or dehydration (DH) of l-asparagine. Hence, we named the domain AMDH.

**Fig. 2:**
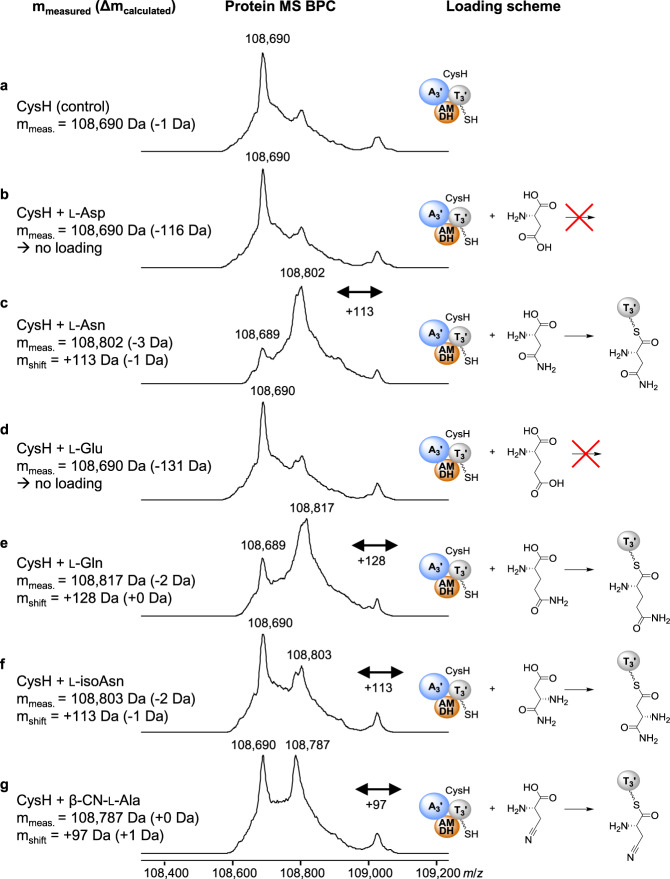
Substrate specificity of CysH. Observed mass shifts in deconvoluted protein MS BPCs reveal loading of l-asparagine (**c**), l-glutamine (**e**), l-isoasparagine (**f**), and β-cyano-l-alanine (**g**) onto CysH. l-aspartic acid (**b**) and l-glutamic acid (**d**) were not accepted by CysH (control: **a**). Blue sphere: adenylation domain; gray sphere: thiolation domain; orange sphere: AMDH domain.

We overexpressed and purified the enzymes CysH, CysH without AMDH domain (CysH∆AMDH), and CysJ from *E. coli* BL21. The enzymes were incubated in vitro individually or in combination using different substrates. Loading of the substrate onto the T domain and subsequent biochemical conversion resulted in mass shifts observed after deconvolution of direct intact protein ESI-MS spectra^26^. First, CysH was incubated with different amino acids to test substrate specificity. Although l-asparagine was favored as substrate by CysH, we also observed loading of l-glutamine, β-cyano-l-alanine, and l-isoasparagine (Fig. 2).

**Fig. 3:**
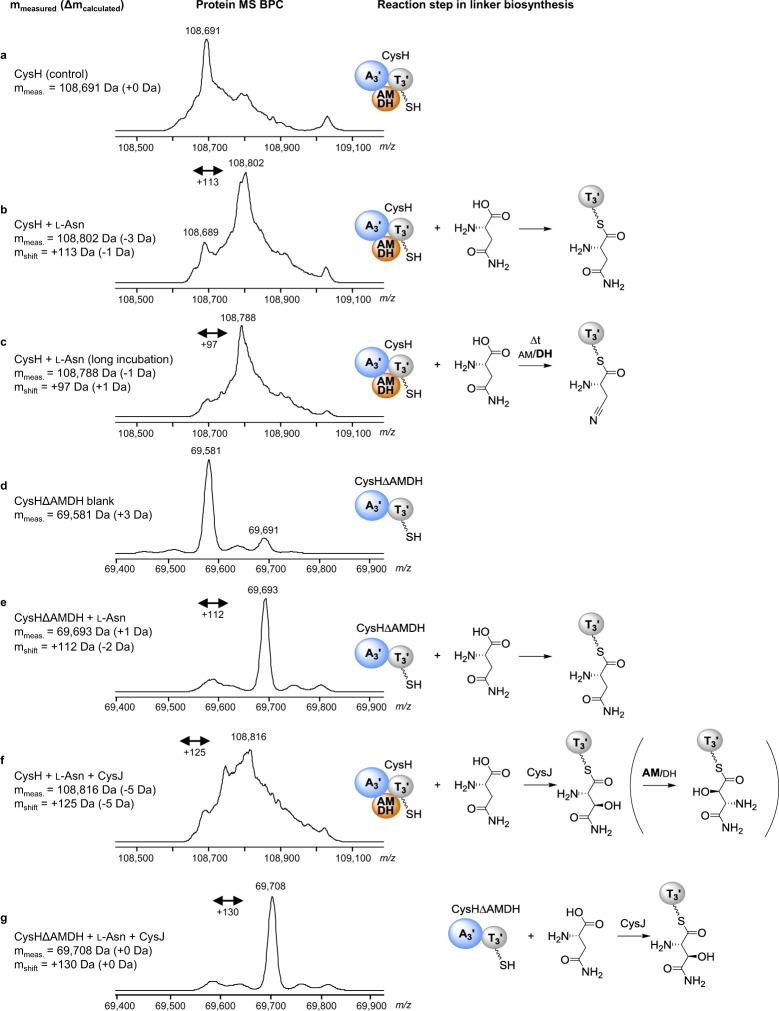
Loading and hydroxylation of l-asparagine on CysH or CysH∆AMDH. Deconvoluted protein MS BPCs (left) reveal different reaction mechanisms (models shown on the right) after in vitro incubation of CysH or CysH∆AMDH with l-asparagine with and/or without CysJ. **a** CysH control. **b** Loading of l-asparagine onto CysH. **c** The dehydratase activity of the AMDH domain leads to dehydration of l-asparagine and formation of β-cyano-l-alanine after prolonged incubation times. **d** CysH∆AMDH control. **e** CysH∆AMDH incubated with l-asparagine. No dehydration of l-asparagine was observed since CysH has no AMDH domain. **f** CysH incubated with l-asparagine and CysJ leads to loading of the substrate onto CysH with subsequent hydroxylation by CysJ (see Fig. 4). The isomerization of (β-hydroxy-)l-asparagine to (α-hydroxy-)l-isoasparagine is shown in parenthesis because this step cannot be observed by MS. The isomerization was confirmed by deletion of the AMDH domain from the BGC and analysis of the production profile after heterologous expression of the respective construct in *M. xanthus* DK1622 (see Fig. 5). **g** CysH∆AMDH incubated with l-asparagine and CysJ leads only to the formation of β-hydroxy-l-asparagine. Blue sphere: adenylation domain; gray sphere: thiolation domain; orange sphere: AMDH domain.

All naturally occurring cystobactamids (except the linker-free derivatives Cys449 and Cys507), which were identified thus far, harbor linker moieties deriving from l-asparagine. Since we also observed acceptance of other substrates by CysH, we assume that substrate specificities of downstream modules in the assembly line hinder the incorporation of different amino acids than l-asparagine. Interestingly, incubation of CysH with l-asparagine for longer than 5 min at room temperature (RT) led to a mass increase of +96 *m/z* instead of +114 *m/z* indicating substrate dehydration (−18 *m/z*) and formation of β-cyano-l-alanine (Fig. 3a–c). Incubation of CysH∆AMDH with l-asparagine only resulted in substrate loading but not in dehydration since only the mass shift expected for l-asparagine was observed even after prolonged incubation (Fig. 2d–e). This experiment was the first indication of the dehydratase activity of the AMDH domain.

**Fig. 4:**
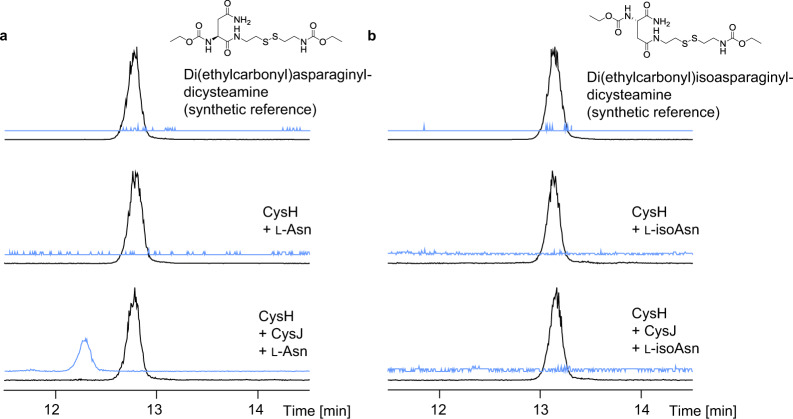
Hydroxylation of CysH-bound l-asparagine by CysJ. HPLC-MS analysis of cysteamine-unloaded and derivatized substrate from the CysH protein. EICs 411.1 *m/z* [M + H]^+^ are shown in black and EICs 427.1 *m/z* [M + H]^+^ (Di(ethylcarbonyl)-hydroxy-l-(iso)asparaginyl-dicysteamine) are shown in blue. **a** Di(ethylcarbonyl)-l-asparaginyl-dicysteamine synthetic reference. CysH-bound l-asparagine unloaded and derivatized showed the same retention time as the synthetic reference. Incubation of CysH with CysJ and l-asparagine lead to hydroxylation of l-asparagine and different retention times of the unloaded substrate compared to the reference. **b** Di(ethylcarbonyl)-l-isoasparaginyl-dicysteamine synthetic reference. CysH-bound l-isoasparagine unloaded and derivatized showed the same retention time as the synthetic reference. No hydroxylation occurred upon the addition of CysJ.

**Fig. 5:**
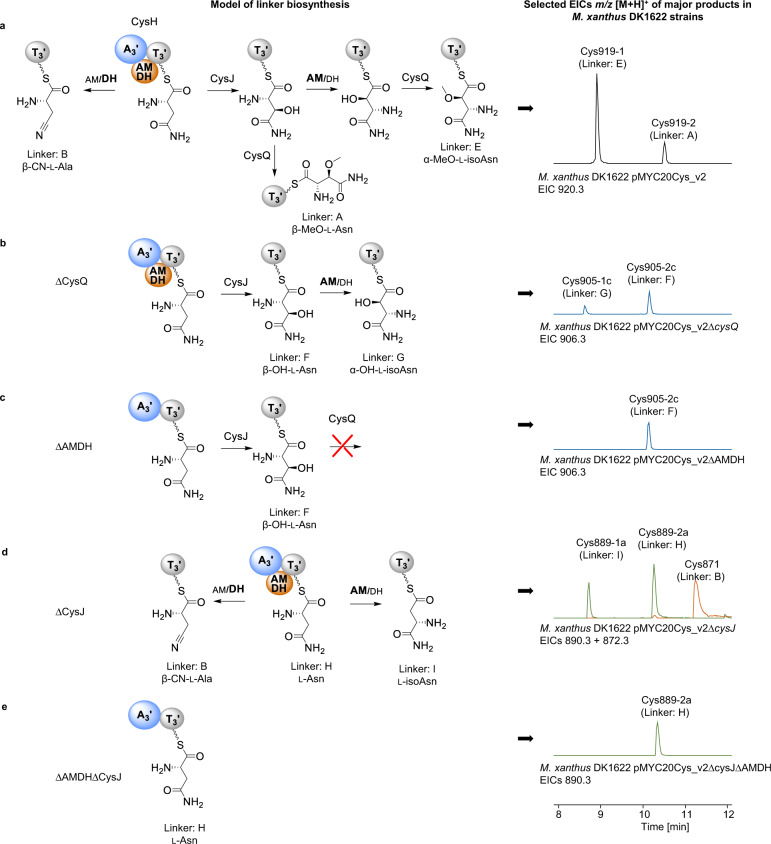
Summary of biosynthesis pathways for cystobactamid linkers. **a** Hydroxylation of CysH-bound l-asparagine by CysJ with subsequent isomerization by AMDH plus *O*-methylation by CysQ leading to α-methoxy-l-isoasparagine (E linker); *O*-methylation without isomerization leads to β-methoxy-l-asparagine (A linker); direct dehydration by AMDH domain leads to the formation of β-cyano-l-alanine (B linker). EIC 920.3 [M + H]^+^ (black) shows the production of Cys919-1 (E linker) and Cys919-2 (A linker) in *M. xanthus* DK1622 pMYC20Cys_v2. **b** Absence/deletion of CysQ leads to the formation of β-hydroxy-l-asparagine (F linker) or α-hydroxy-l-isoasparagine (G linker) after isomerization by AMDH. EIC 906.3 [M + H]^+^ (blue) shows the production of Cys905-1c (G linker) and Cys905-2c (F linker), which lack one methyl group in the linker compared to Cys919 (−14 Da shift). **c** Deletion of the AMDH domain leads to hydroxylation of CysH-bound l-asparagine by CysJ, but *O*-methylation by CysQ does not occur (only formation of β-hydroxy-l-asparagine/production of Cys905-2c). **d** Absence/deletion of CysJ leads to the formation of l-asparagine (H linker), l-isoasparagine (I linker), or β-cyano-l-alanine (B linker). Overlay of EIC 890.3 [M + H]^+^ (green) and EIC 872.3 [M + H]^+^ (orange) shows the production of Cys889-1a (I linker), Cys889-2a (H linker) and Cys871 (B linker) **e** Deletion of CysJ and the AMDH domain prevents any modification of asparagine leading only to the production of Cys889-2a. Blue sphere: adenylation domain; gray sphere: thiolation domain; orange sphere: AMDH domain; red cross: nonfunctional pathway.

Next, we investigated the β-hydroxylation of l-asparagine, which was speculated to be catalyzed by CysJ on T domain-bound substrate in an α-ketoglutarate (α-KG)-dependent reaction^1^. Incubation of CysJ with free l-asparagine and α-KG with subsequent analysis using thin-layer chromatography (TLC) indicated no hydroxylation of free l-asparagine (see Supplementary Method 6 and Supplementary Fig. 16). Since CysJ also revealed high structural similarity to SyrP^27^, which has been shown to catalyze β-hydroxylation of T domain-bound aspartyl residues in syringomycin biosynthesis, we expected an in trans tailoring step of CysJ in coordination with CysH. However, we observed significant peak broadening during protein MS upon incubation of CysH with l-asparagine and CysJ (Fig. 3f), preventing clear indication of the β-hydroxylation. Interestingly, incubation of CysH∆AMDH with l-asparagine and CysJ could be analyzed without peak broadening and β-hydroxylation of l-asparagine could be confirmed (Fig. 3g). To prove β-hydroxylation of l-asparagine in presence of the AMDH domain, we incubated CysH with CysJ and l-asparagine and subsequently unloaded the carrier protein-bound intermediate via trans-thioesterification using cysteamine^28^, which was analyzed using UPLC-MS after further derivatization (Fig. 4; see Supplementary Method 7 and Supplementary Fig. 17). We observed a different mass and retention time of the unloaded substrate in the presence of CysJ which confirms the in trans-β-hydroxylation of CysH-bound l-asparagine. Surprisingly, we could not observe α-hydroxylation of CysH-bound l-isoasparagine by CysJ. Consequently, the β-hydroxylation of l-asparagine occurs prior to the expected aminomutase reaction. However, in this set of in vitro experiments, we were unable to detect the α,β-aminomutase activity of the AMDH domain, because the expected isomerization of l-asparagine cannot be observed by MS. We thus performed numerous targeted gene and domain deletion experiments with subsequent heterologous expression of the modified BGC in *M. xanthus* DK1622 (Fig. 5).

Most importantly, the deletion of the AMDH domain in *cysH* resulted in the abolishment of the production of all cystobactamids with l-isoasparagine or β-cyano-l-alanine linkers in the heterologous producer. This result can be taken as experimental proof confirming the AMDH domain asparaginyl α,β-aminomutase activity. Surprisingly, the major product of this construct was the unnatural derivative Cys905-2c carrying a β-hydroxy-l-asparagine linker instead of the expected β-methoxy-l-asparagine (Fig. 5c). The production of the unnatural Cys905-1c and Cys905-2c derivatives, both lacking *O*-methylation in the linkers, was also achieved through deletion of the gene *cysQ* encoding an *O*-methyltransferase (Fig. 5b). We speculate that the abolishment of *O*-methylation in absence of the AMDH domain in CysH is linked to protein-protein interaction between AMDH and CysQ. These results allowed us to devise a biosynthesis model for the production of the native β-methoxy-l-asparagine (linker A), the α-methoxy-l-isoasparagine (linker E), and the β-cyano-l-alanine (linker B) moieties, as shown in Fig. 5a. In the presence of CysH (including the AMDH domain), CysJ, and CysQ, the major products were Cys919-1 and Cys919-2 harboring linkers E and A, respectively. However, Cys871 with linker B was not detected in the cultivation broth of the heterologous producer but previously described in native producer strains^2^. As shown in Fig. 3b, c, dehydration of l-asparagine by the AMDH domain mainly occurs in absence of CysJ. We assume that the activity of CysJ in the heterologous producer prevented the AMDH domain from dehydrating l-asparagine. To test this hypothesis, we deleted *cysJ* in the modified BGC, which indeed lead to heterologous production of substantial amounts of Cys871 (Fig. 5d) confirming our previous assumption. Consequently, we hypothesize that, in presence of CysJ, β-hydroxylation of l-asparagine occurs much faster than dehydration of l-asparagine by the AMDH domain. Furthermore, two unnatural major derivatives, namely Cys889-1a and Cys889-2a, were produced (Fig. 5d; Supplementary Fig. 15). Cys889-1a and Cys889-2a lack the methoxy group in the linker, thus only having either l-isoasparagine (linker I) or l-asparagine (linker H), because neither hydroxylation by CysJ nor *O*-methylation by CysQ can occur. Interestingly, the isomerization of l-asparagine still occurs, but now leads to a much less abundant product. We speculate that the isomerization of β-hydroxyl-l-asparagine by the AMDH domain is more efficient than the isomerization of l-asparagine, or that CysH, CysJ, and CysQ form a protein complex influencing the reactivity of the AMDH domain. If CysJ is deleted, the isomerization reaction may occur much slower, thus leading to a six to eightfold decreased l-isoasparagine/l-asparagine linker ratio—compared to the α-methoxy-l-isoasparagine/β-methoxy-l-asparagine ratio in Cys919-1 and Cys919-2—and an increased probability of l-asparagine dehydration. Finally, we generated an expression construct with a double deletion of both *cysJ* and the AMDH domain. As expected, only Cys889-2a featuring a simple l-asparagine linker was produced, whereas neither Cys889-1a nor Cys871 could be detected (Fig. 5e). This experiment again proves that the AMDH domain catalyzes either dehydration or aminomutation of l-asparagine. The type of reaction catalyzed by the AMDH domain depends on the preceding hydroxylation of the substrate by CysJ. Notably, after deletion of *cysJ* or the AMDH domain, we also identified four minor unnatural cystobactamids for which we were not able to propose putative structures (Supplementary Method 5; Supplementary Fig. 15).

Including all results of the in vitro and in vivo experiments, we are able to provide biosynthesis schemes for the α-methoxy-l-isoasparagine linker and also other linker derivatives, which are summarized in Fig. 5. Although CysH loads a variety of amino acids, l-asparagine is the favored substrate, which is either directly dehydrated by AMDH to form β-cyano-l-alanine or β-hydroxylated by CysJ with subsequent isomerization by AMDH. CysQ performs the *O*-methylation of β-hydroxy-l-asparagine or α-hydroxy-l-isoasparagine leading to the formation of β-methoxy-l-asparagine or α-methoxy-l-isoasparagine (Fig. 5a), respectively. Furthermore, we speculated that the product ratio of cystobactamid derivatives with different linkers is highly dependent on the reaction kinetics of the enzymes involved in linker biosynthesis. In absence of CysJ substantially higher production of cystobactamid with the dehydrated linker was observed (Fig. 5d). Furthermore, heterologous expression of constructs with deleted AMDH domain resulted in the elimination of production of cystobactamids with l-isoasparagine linkers, which only showed weak or no antibacterial activity^2^.

Our findings suggest that the AMDH domain performs both amide dehydration and aminomutation via an unknown mechanism. Structure prediction was precluded by unsuccessful crystallization attempts. Additionally, no known templates with a crystal structure were available, preventing in silico 3D modeling. A protein BLAST query of the AMDH domain identified numerous homologs, all inserted into A domains with l-asparagine-specificity based on Stachelhaus prediction^18^. Since none of these homologs could be linked to a known secondary metabolite, we speculate that the dehydration or isomerization of l-asparagine is a common mechanism in the biosynthesis of hitherto unknown natural products. We analyzed the 25 closest BLAST homologs of the AMDH domain in silico and identified seven conserved core motif regions (Supplementary Fig. 10) that might be required for catalysis or folding. Core region 1 contains a highly conserved ATP-binding motif (SGGKD), which was also found in the homologous domain in AlbIV (Supplementary Fig. 9a) and hypothesized to be involved in l-asparagine dehydration^9^. To investigate if the AMDH domain shares any homology with known aminomutases, we searched for conserved sequence motifs such as an ASG motif described in tyrosine and phenylalanine aminomutases that contain the cofactor 4-methylideneimidazole-5-one^29,30^. No such motif was identified, preventing comparison to this class of aminomutases. Interestingly, we observed that CysH shows a dark brown color after overexpression and purification from *E. coli*, indicating the presence of metal as a cofactor. Since we did not identify a CxxCxxxC motif serving as Fe–S cluster binding site^31^, we assume that a radical mode of action is unlikely for the AMDH domain, even if a few radical-SAM proteins were described not harboring this motif^32^. Although the enzymatic mechanism of amide dehydration to nitriles is not yet known, the reverse reaction catalyzed by a heterodimeric nitrile hydratase has been described. Interestingly, this reaction relies on a single, cysteine-bound iron atom^33^. Summarized, we consider a radical mode of action or a similar one to known aminomutases unlikely. We speculate that the AMDH domain involves a not yet described mode of catalysis, which may require a metal cofactor or might be similar to the ATP-dependent reaction proposed for albicidin formation and certainly deserves future investigation.

### Shuttling of the linker moiety to the assembly line by CysB

**Fig. 6:**
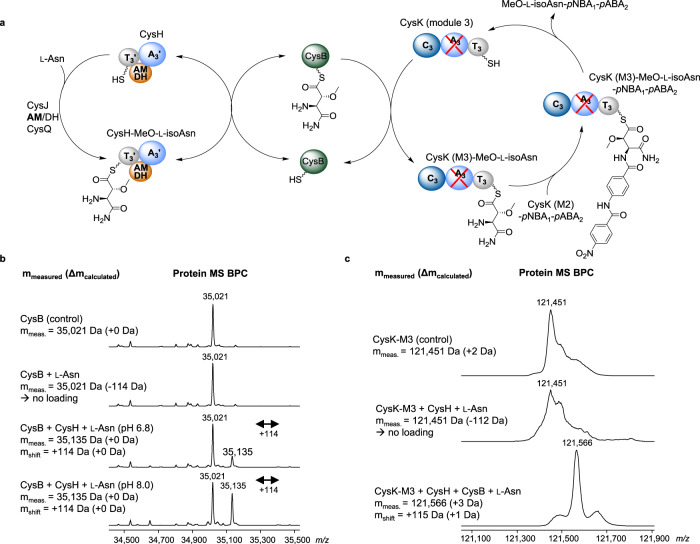
CysB-mediated transfer of β-methoxy-l-isoasparagine from CysH to CysK. **a** Model for the CysB-mediated shuttling process. l-asparagine is activated by CysH and modified by CysJ, the AMDH domain, and CysQ as shown in Fig. 5. β-methoxy-l-isoasparagine is transferred from CysH to module 3 of CysK (M3) by CysB (green sphere). Condensation of the linker moiety with the *p*NBA_1_-*p*ABA_2_ dipeptide leads to the formation of the shown tripeptide. **b** Deconvoluted protein MS analysis of CysB control; CysB incubated with free l-asparagine does not result in CysB loading; CysB incubated with l-asparagine and CysH leads to pH-sensitive loading of CysB (+114 *m/z* shift). **c** Protein MS analysis verifies the transfer of l-asparagine from CysB to CysK (M3) in the presence of CysH. Blue sphere: adenylation domain; gray sphere: thiolation domain; orange sphere: AMDH domain; red cross: inactive domain.

Another intriguing feature of cystobactamid biosynthesis is the incorporation of the linker moiety into the NRPS assembly line. Interestingly, the A domain of module 3 in CysK (CysK-M3) was proposed to be inactive, because the catalytic lysine residue in core motif A10 is missing^1^. We performed direct intact protein MS analysis to confirm experimentally that l-asparagine is not accepted by module 3 (Fig. 6c), which was separately overexpressed and purified, because of the large size of CysK (507 kDa).

Initially, the overexpression yields of CysK-M3 were very low, but we overcame this problem by coexpressing CysA, which is an MbtH-type A domain activator protein supposed to be required for expression and activity of NRPS modules^34^. Incubation of CysK-M3 with l-asparagine and subsequent full protein MS analysis confirmed that no substrate loading occurred. Thus, we hypothesize that loading of the respective T_3_ domain requires an in trans shuttling process between CysH and CysK via a third enzyme.

BLAST analysis of the genes in the cystobactamid BGC showed the similarity of CysB to SyrC (35% similarity/20% identity), which is a (chloro)threonyl aminoacyl transferase in syringomycin biosynthesis^35^. Another CysB homolog, CmaE, was shown to shuttle aminoacyl groups between carrier protein domains in coronamic acid biosynthesis^36^. With CysB being our candidate enzyme for the hypothesized shuttling process, we overexpressed and purified CysB, CysH, and CysK-M3 from *E. coli* BL21. First, we analyzed the aminoacyl transfer reaction of l-asparagine from CysH to CysB using protein MS. We used commercially available l-asparagine instead of methoxylated l-(iso)asparagine derivatives since the deletion of *cysJ* from the BGC with subsequent heterologous expression in *M. xanthus* DK1622 showed that l-asparagine is also accepted by the assembly line (Fig. 5d). Protein MS analysis showed that CysB was only partially loaded with l-asparagine in the presence of CysH because the major peak still derived from unloaded CysB, whereas no free l-asparagine was loaded (Fig. 6b). To exclude that the partial loading is caused by inappropriate reaction conditions, we tested different pH values. Even though the equilibrium between l-asparagine loaded CysH and CysB shifted pH dependently, we still observed partial loading of CysB. Thus, we conclude that the reaction is reversible. Next, we analyzed the transfer of CysB-loaded l-asparagine to CysK-M3. Interestingly, loading of l-asparagine from CysB to module 3 of CysK was almost stoichiometric (Fig. 6c), implying that this second part of the shuttling process probably drives the cycle towards the transfer from CysH to CysK-M3. With those experiments, we confirmed the hypothesis that CysB mediates the shuttling process of the linker moiety between CysH and CysK-M3 and provide a respective model in Fig. 6a.

### Revision of the complete cystobactamid biosynthesis model

**Fig. 7:**
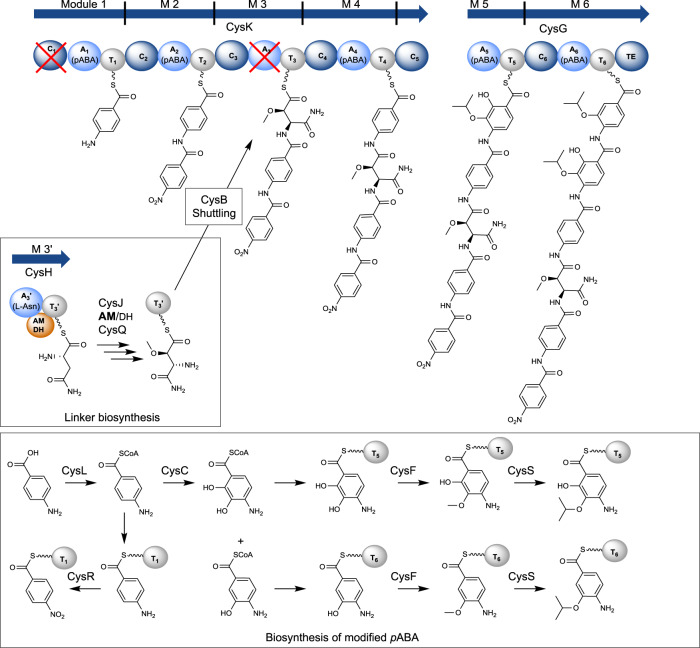
Revised biosynthesis of cystobactamids on the example of Cys919-1. C: condensation domain (dark blue sphere), A: adenylation domain (light blue sphere), T: thiolation domain (gray sphere), TE: thioesterase domain (dark blue sphere), AMDH: aminomutase dehydratase domain (orange sphere), red cross: inactive domains. Biosynthesis of the α-methoxy-l-isoasparagine linker moiety is described in more detail in Fig. 5. The 6-modular assembly line is encoded by *cysK* and *cysG* (blue arrows). CysR converts *p*ABA to *p*NBA in trans or after product release from the assembly line. Biosynthesis of 2-isopropoxyl-*p*ABA and 2-isopropoxyl-3-hydroxy-*p*ABA is presumably catalyzed by CysC, CysF, and CysS. *p*ABA is incorporated by M 1 and M 2, respectively. The linker moiety is transferred from T_3’_ (CysH) to T_3_ (CysK) by CysB (see Fig. 6). Another *p*ABA is incorporated by M 4. Two tailored *p*ABAs are incorporated by M 5 and M 6.

Based on our findings regarding the linker biosynthesis and transfer and incorporation into the assembly line, we provide a revised biosynthesis model on the example of Cys919-1 (Fig. 7). The biosynthesis of the cystobactamid peptide scaffold starts with CysK. Separate overexpression and purification of modules 1, 2, and 4 (CysK) with subsequent in vitro loading experiments revealed in the protein MS analyses that various *p*ABA derivatives are accepted (Supplementary Fig. 11). Interestingly, module 1 does not accept *p*NBA as substrate, which means that the oxygenation of *p*ABA_1_ is performed in trans or after final product release. Deletion of *cysR* and subsequent heterologous expression of the respective construct in *M. xanthus* DK1622 lead to the production of derivatives with an N-terminal amine, thus proving that CysR is the *p*ABA-*N*-oxygenase (Supplementary Fig. 12). CysL is assumed to be a *p*ABA-CoA ligase that activates free *p*ABA^1^. We assume that the oxidation of CoA-bound *p*ABA is performed by CysC forming 3-hydroxy-*p*ABA and 2,3-dihydroxy-*p*ABA prior to incorporation into the assembly line by modules 5 and 6 (CysG), respectively. CysC is homologous to the benzoate oxidase BoxB, which is described as dioxygenase requiring a CoA-activated substrate^37^. In addition, deletion of *cysC* and heterologous expression of the construct in *M. xanthus* DK1622 lead to complete abolishment of cystobactamid production. This underlines the importance of *p*ABA_5_ and *p*ABA_6_ hydroxylation prior to their activation by A_5_ and A_6_ from CysG. We separately overexpressed and purified modules 5 and 6 from *E. coli* BL21 and analyzed which substrates are processed in vitro. Likewise, for modules 1, 2, and 4, protein MS analysis revealed loading of various *p*ABA derivatives by modules 5 and 6, respectively (Supplementary Fig. 13).

CysF is a SAM-dependent methyltransferase assumed to be involved in the formation of 2-hydroxy-3-methoxy-pABA on module 5 and 3-methoxy-pABA on module 6. The final tailoring steps of *p*ABA_5_ and *p*ABA_6_ are iterative methyl group alkylations leading to various branched alkoxy groups. Those reactions are performed by CysS, a cobalamin-dependent radical-SAM enzyme^38^.

We observed another special feature of the cystobactamid biosynthesis when we deleted *cysB* from the BGC and subsequently expressed the modified construct in *M. xanthus* DK1622. Surprisingly, Cys507, a tripeptide consisting only of the three C-terminal (tailored) *p*ABAs, was still produced as the only derivative (Supplementary Fig. 18). This not only proves CysB being indispensable for the biosynthesis of full-length cystobactamids but also that Cys507 is not a degradation product as initially thought^1^. Instead, the biosynthesis can also start from module 4 on, which is contradictory to the processivity rule in NRPSs.

## Discussion

Our results present features in NRPS synthesis demonstrating that the AMDH domain performs both dehydration and aminomutation of l-asparagine in a single domain. The bifunctionality and ability to perform completely different biochemical reactions dependent on preceding tailoring steps enables the production of a variety of different compounds with a yet unknown mechanism. We excluded a radical mode of action and refuted similar mechanisms to known tyrosine- and phenylalanine aminomutases^29,30^. Further biochemical characterization and crystallization experiments are necessary to elucidate the underlying mechanisms of the AMDH domain in the future. The mutation of conserved amino acid residues in the core motifs of the AMDH domain, e.g., in the ATP binding site, might inactivate only one of the two functions and does not pose the risk to disrupt protein–protein interactions as compared to deletion of the entire domain. Interestingly, we found numerous unannotated homologous domains in several BGCs of unknown function, indicating that this AMDH domain is involved in the biosyntheses of a significant number of yet-to-be-identified natural products. Consequently, the AMDH domain can be queried to identify additional rarely observed modified l-asparagine-containing natural products and their derivatives.

Interestingly, no albicidin derivative harboring an l-isoasparagine linker has been identified so far, indicating that the AMDH domain homolog in the albicidin biosynthesis might not be able to perform an aminomutation-type reaction. Furthermore, von Eckardstein and coworkers described an albicidin derivative with a methoxylated β-cyano-l-alanine linker, a linker-type not found in cystobactamids. Based on our experiments, we hypothesized that β-hydroxylation of l-asparagine occurs much faster than dehydration and that β-hydroxylation prevents the AMDH domain from dehydrating the substrate. This explains why we only identified a considerable amount of Cys871 harboring a β-cyano-l-alanine linker after deletion of the hydroxylase CysJ. However, the existence of the methoxylated β-cyano-l-alanine linker in albicidin either means that AlbVIII (the homolog of CysJ) is able to hydroxylate β-cyano-l-alanine or that the AMDH domain homolog in AlbIV is able to dehydrate β-hydroxy-l-asparagine (both of which was not observed in the cystobactamid biosynthesis) or that another enzyme than AlbVIII catalyzes the hydroxylation of β-cyano-l-alanine. Since Cys871 is only a very minor derivative in the presence of CysJ, one could also speculate that CysJ, likewise to AlbVIII, is able to hydroxylate β-cyano-l-alanine but the generated cystobactamid derivatives with methoxylated β-cyano-l-alanine linkers are only produced in such minor amounts that the ion intensity in MS does not exceed the detection limit. In this case, the inactivation of the aminomutation function of the AMDH domain in CysH might lead to increased production of Cys871 and the detection of methoxylated β-cyano-l-alanine linkers in cystobactamids. An alternative experiment would be the exchange of the AMDH domain in CysH by its homolog from AlbIV, which could potentially lead to a more pronounced dehydration reaction and subsequent hydroxylation by CysJ. In any case, it needs further experimental investigation to understand the order and under which circumstances the respective linker modifications take place in the cystobactamid and albicidin biosyntheses.

The modification of amino acids by independent NRPS modules and subsequent incorporation into nascent polypeptides in the assembly line is not an unknown phenomenon in natural product biosynthesis as similar findings have been reported for novobiocin, nikkomycin, and vancomycin^39–41^. In those examples, a TE releases the modified amino acid from the T domain of the independent module on which the modification reaction occurs. The free modified amino acid is then tethered to the core peptide by A domain reactivation or by a specific ligase. However, in cystobactamid biosynthesis, the shuttling process of the modified l-asparagine between the independent module CysH and the assembly line is mediated by CysB. The differences in the kinetics of the first (CysH to CysB) and second (CysB to CysK-M3) part of the reaction are highly pH-dependent, thus indicating that the reaction might be driven by pI differences between the T domains of CysH (pI = 5.6) and CysK-M3 (pI = 6.3). A similar correlation was also proposed for the CysB homolog CmaE^36^. Notably, we demonstrated that the assembly line is able to start the biosynthesis from module 4 on, skipping the first three modules, when we deleted *cysB* and heterologously expressed the modified construct in *M. xanthus* DK1622. This leads to an interruption of the shuttling process and the production of the *N*-terminally truncated, linker-free cystobactamid derivative Cys507. Consequently, the cystobactamid biosynthesis shows exceptions for two common NRPS rules, the collinearity and the processivity rule, which underlines the diversity in the functionality of NRPS systems. The production of Cys507 even in the presence of the shuttling protein CysB shows that the transfer of the linker moiety to the assembly line is a bottleneck for the production of full-length cystobactamids.

Finally, we demonstrated that the heterologous expression platform can be used to produce a variety of previously unknown cystobactamids by genetic engineering of the BGC. Despite serious efforts, we were not able to isolate sufficient quantities of those derivatives, because they are present in much lower concentrations compared to the major product Cys919-1. Furthermore, the cystobactamid production decreased substantially when we scaled up the cultivation from 50 mL to 1.5 L, which may also explain the low formerly reported yields. This drop-in production was even worse for unnatural cystobactamids, for which even the production of the major products was a fraction compared to Cys919-1. Therefore, we relied on exact HRMS data and MS^2^ fragmentation in this study. We assigned the stereocenters of the derivatives based on the stereochemistry of previously described cystobactamids. Furthermore, we assigned the linker moieties with the same masses (e.g., linker A and E) based on different retention times that were also observed for previously reported cystobactamids, in which derivatives harboring l-isoasparagine linkers always eluted first. However, the establishment of a robust fermentation process combined with media optimization has to be addressed in future experiments to enable purification, NMR verification, and determination of the antibacterial activity of the cystobactamid derivatives from this study. Moreover, the deletion of the AMDH domain may be used in the future to drive the heterologous production profile towards cystobactamids with l-asparagine rather than l-isoasparagine linkers. Notably, cystobactamids with l-asparagine linker showed superior antibacterial activity against numerous human pathogens like *A. baumanii*, *Citrobacter freundii*, *E. coli*, *Enterobacter cloacae*, *P. aeruginosa*, *Proteus vulgaris*, *Bacillus subtilis*, *Staphylococcus aureus*, and *Streptococcus pneumoniae*^1,2^. Thus, the question arises why Nature established such a complex biosynthesis route including a *trans*-acting independent NRPS module with a shuttling process to produce cystobactamids that are biologically less active? It was previously shown^1,2^ and confirmed in this study that naturally a whole cocktail of cystobactamids is produced. Even though cystobactamids with l-asparagine linkers exhibited superior antibacterial activity against a small panel of tested human pathogens, the natural producer strains have to outcompete a myriad of rival strains in their natural environment. It thus appears likely that the diversity of cystobactamids produced helps the natural producers to gain the advantage over a variety of their competitors. Furthermore, it cannot be excluded that cystobactamids possess another function apart from their antibacterial activity, e.g., the involvement in developmental processes of the cell. However, from a human point of view, the simplest solution to produce more active cystobactamids with medicinal relevance harboring l-asparagine linkers would be the existence of an active l-asparagine-specific CysK-A_3_ domain. Restoring the activity of the natively inactive A_3_ domain by genetic engineering of the assembly line and thus bypassing the production-limiting shuttling process will be addressed in future experiments.

## Methods

### Cultivation of strains

*E. coli* DH10β, HS996, and NEB10β strains were used for cloning the modified BGC. *E. coli* BL21 (DE3) was used for recombinant protein expression. Cultivation was performed in LB medium (10 gL^−1^ tryptone, 5 gL^−1^ NaCl, 5 gL^−1^ yeast extract, pH 7.6) at 37 °C or 30 °C (handling plasmids larger than 15 kb). Protein expression experiments were carried out at 37 °C and 16 °C after induction (see protein expression section). Ampicillin (100 µg mL^−1^), chloramphenicol (34 µg mL^−1^), kanamycin (50 µg mL^−1^), and oxytetracycline (10 µg mL^−1^) were used as selection markers. *Myxococcus xanthus* DK1622 was used as a heterologous expression host. *Cystobacter velatus* Cbv34 and *Myxococcus fulvus* SBMx122 are native cystobactamid producer strains and were used for the isolation of genomic DNA or controls in production screening experiments, respectively. Cultivation was done in CTT medium (10 g L^−1^ casitone, 1.21 gL^−1^ TRIS, 8 mM MgSO_4_, 1 mM KH_2_PO_4_, pH 7.6) to grow cells for genomic DNA isolation, transformations, or for starting cultures prior to production screening cultivations. M7/s4 medium (5 g L^−1^ soy flour, 5 g L^−1^ corn starch, 2 g L^−1^ glucose, 1 g L^−1^ yeast extract, 1 g L^−1^ MgSO_4_ × 7H_2_O, 1 g L^−1^ CaCl_2_ × 2H_2_O, 10 g L^−1^ HEPES, pH 7.4; supplemented with 0.1 mg L^−1^ of vitamin B_12_ and 5 mg L^−1^ of FeCl_3_ after autoclaving) was used for 50 mL screening cultures. M7/s4 pre cultures (without supplements) were inoculated from CTT agar starting cultures. Screening cultures were inoculated from 1 to 3 days old M7/s4 pre cultures (10% (v/v) inoculation volume) and cultivated for 5 days. Heterologous gene expression in *M. xanthus* was induced after 1 day by adding vanillate (1 mM final concentration). XAD16 absorber resin was added after 2 days (2% (v/v)). All liquid cultivations were performed in baffled Erlenmeyer flasks on an orbital shaker at 160 rpm at 30 °C. Kanamycin (50 µg mL^−1^) and oxytetracycline (10 µg mL^−1^) were used as selection markers when cultivating heterologous *M. xanthus* strains. *Saccharomyces cerevisiae* ATCC4004247 was used for TAR cloning. Cultivations were performed at 30 °C in YPAD medium (20 g L^−1^ glucose, 10 g L^−1^ peptones, 10 g L^−1^ yeast extract, 100 mg L^−1^ adenine-hemisulfate, pH 7.0). YNB medium (20 g L^−1^ glucose, 8 g L^−1^ YNB base w/o leucine, 2 g L^−1^ amino acid mix w/o leucine, 100 mg L^−1^ adenine-hemisulfate, pH 7.0) was used for the selection of transformants.

### In silico experiments and revision of the native BGC sequence

All in silico experiments, including the analysis and sequence revision of the native cystobactamid BGC from Cbv34 and the design of the modified BGC and the cloning and expression vector system pMYC, are described in detail in Supplementary Method 1–3. Resequencing of *cysK* was performed using Sanger sequencing. We deposited the sequences related to this manuscript in the GenBank database under the following accession numbers: revised native cystobactamid BGC (KP836244.2), modified cystobactamid BGC (MT572315), basic pMYC (MT572316), pMYC20 (MT572317), and pMYC21 (MT572318).

### DNA synthesis and BGC and pMYC assembly

The modified BGC and the cloning and expression vector system pMYC were synthesized in fragments (fragment description listed in Supplementary Table 3). DNA synthesis was carried out by ATG: biosynthetics GmbH. Sequence-verified DNA synthesis fragments were delivered in pGH standard vector harboring an *ampR* (*bla*) gene for selection on ampicillin. The assembly of the BGC and the vector system are described in more detail in Supplementary Method 2 and 3. Restriction endonuclease hydrolysis and DNA ligation were performed according to the manufacturer’s information protocols. *E. coli* transformation was carried out via electroporation in cuvettes with 1 mm electrode distance using 1300 Vcm^−1^, 10 µF, and 600 Ω as conditions. The transformed cells were resuspended in 1 mL LB medium and incubated for 90 min before selection on LB agar medium overnight (see also “Cultivation of strains”). Selected clones were cultivated in 5 mL of liquid LB medium for 16 h and plasmid DNA was subsequently isolated from 2 mL using alkaline lysis. The correctness of the isolated constructs was verified by restriction hydrolysis. pMYC20 and pMYC21 were generated from the DNA fragments pMYC and Mx8-tetR or Mx9-kanR by ligation after hydrolysis with *Pac*I and *Xma*JI, respectively. Both operons of the modified BGC (CysOp1 and CysOp2) were assembled in two separate TAR cloning reactions using pMYC20 or pMYC21, respectively. For TAR cloning *S. cerevisiae* ATCC4004247 was cultivated in liquid YPAD medium to OD_600_ 2.0 (~2 × 10^7^ cells mL^−1^). The culture was harvested at 4 °C and washed once in ice-cold ddH_2_O (1/2 of the cultivation volume). The cells were finally resuspended in ice-cold ddH_2_O (1/10 volume of the culture). One transformation mixture contained 500 µL of resuspended cells (~1 × 10^8^) and 360 µL linear DNA fragment mix (240 µL 50% (w/v) PEG3350, 50 µL sheared salmon sperm DNA (2 mg mL^−1^, boiled for 5 min at 99 °C), 36 µL lithium acetate (65.99 g L^−1^ in TE buffer) and 34 µL DNA fragments in ddH_2_O). For CysOp1 assembly, a total amount of 800 ng of DNA was used and for CysOp2 350 ng, whereas each DNA fragment was added in equimolar amount. The transformation mixture was incubated for 30 min at 30 °C prior to heat shock for 45 min at 42 °C. After centrifugation the supernatant was removed, the cells were resuspended in 100 µL ddH_2_O and cultivated on a solid YNB selection medium. After 4 days, selected clones were cultivated in liquid YNB medium and plasmid DNA was isolated using a modified alkaline lysis protocol: 2 mL of the YNB culture were harvested and resuspended in 250 µL zymolyase-containing resuspension buffer (20.21 g L^−1^ Na_2_HPO_4_, 3.39 g L^−1^ NaH_2_PO_4_, 218.60 g L^−1^ sorbitol, 5 U µL^−1^ zymolyase, pH 7.5). The suspension was incubated for 60 min at 37 °C and subsequently, we proceeded with the standard alkaline lysis protocol. The isolated plasmid DNA from *S. cerevisiae* ATCC4004247 was subsequently transformed into *E. coli* DH10β. Clones harboring the correct plasmids were verified by restriction hydrolysis of plasmid DNA isolation via standard alkaline lysis. Some DNA fragments were assembled in vitro to obtain larger fragments before TAR assembly. The *cysK* was replaced by a dummy sequence in order to minimize the risk of unspecific recombination during TAR assembly. The fragments of *cysK* harboring repetitive sequence segments were assembled separately in vitro by a three-step restriction/ligation cloning strategy using *Bsa*I (Supplementary Fig. 4) prior to its assembly with the rest of the cluster. Supplementary Fig. 5 schematically depicts all cloning steps performed to obtain the final expression construct pMYC20Cys_v2. Supplementary Table 6 summarizes all in vitro cloning steps performed in this work.

### Genetic manipulation of expression constructs

Red/ET recombineering^42^ in combination with restriction hydrolysis and re-ligation was used to delete (part of) genes from the plasmids. Amplification of *ampR* (*bla*) gene from pUC18 was done via PCR. Apart from the pUC18 binding site, primers contained *Bsa*I R-sites and 50 bp sequences that are homologous to the gene, which was deleted. Supplementary Data 3 summarizes all primers used for this experiment. For Red/ET recombineering 1.4 mL LB medium was inoculated with *E. coli* GB05-red and grown at 37 °C to OD_600_ 0.2. After addition of 40 µL 10% (w/v) l-arabinose, the cultivation was continued until OD_600_ 0.4 was reached. The cells were harvested, washed twice in ice-cold ddH_2_O, and finally resuspended in 30 µL of ddH_2_O. The *ampR* (*bla*) PCR product was transformed together with pMYC20Cys_v2 or pMYC20Cys_v2∆AMDH into *E. coli* GB05-red via electroporation (described above). Plasmid DNA from the fully overgrown selection plate (oxytetracycline and ampicillin) was isolated using alkaline lysis, followed by another transformation step of 100 ng of the isolated plasmid mixture into *E. coli* NEB10β. Again, we selected clones harboring the correct recombination products on ampicillin and oxytetracycline. After the plasmid isolation, we verified the clones by restriction analysis. Next, the recombination product was hydrolyzed with *Bsa*I and re-ligated to remove *ampR* (*bla*) from the construct. Clones, which lost their resistance towards ampicillin, were selected for plasmid isolation and restriction analysis. Supplementary Table 7 lists all manipulated plasmids that were generated in this study. Supplementary Data 1 and Supplementary Data 2 summarize all strains and plasmids generated during the cloning process, respectively.

### Transformation of *M. xanthus* DK1622 and verification by colony PCR

Expression constructs were transformed into *M. xanthus* DK1622 via electroporation. Therefore, 2 mL of a CTT overnight culture was harvested, washed twice with 1 mL ddH_2_O and resuspended in 35 µL ddH_2_O. Next, 10 µL of DNA solution (~1–3 µg) was added to the cell suspension. Transformation by electroporation was carried out in cuvettes with 1 mm electrode distance using 650 Vcm^−1^, 25 µF, and 400 Ω as conditions. The transformed cells were resuspended in 1 mL CTT medium and incubated for 6 h under constant shaking to prevent sinking of the cells. Afterward, the transformants were mixed with 3 mL of CTT soft agar (CTT with only 7.5 g L^−1^ agar) and poured onto a CTT agar plate. After hardening of the soft agar layer with embedded transformant cells, the plates were incubated at 30 °C for 5 days. Due to their embedded status in the soft agar layer, the growing transformant colonies do not spread over the agar surface allowing the isolation of single clones and transfer onto a fresh CTT agar plate. Integration of the expression constructs into the chromosome occurred by site-specific phage recombination in the Mx8 attachment site. Integration was verified by PCR using different combinations of the primers Mx8-attB-up2, Mx8-attB-down, Mx8-attP-up2 and Mx8-attP-down (Supplementary Data 3) giving PCR products of the following sizes: Mx8-attB-up2/Mx8-attPdown (427 bp) and Mx8-attP-up2/Mx8-attB-down (403 bp). The primer combination mx8-attB-up2/Mx8-attBdown (449 bp) served as negative control and only revealed a PCR product for wild-type *M. xanthus* DK1622, but not for the strains with integrated expression constructs. To obtain the template DNA required for PCR verification, 5 mm² of spread *M. xanthus* DK1622 transformants were scratched from a CTT agar plate and resuspended in 100 µL of ddH_2_O. The cells were lysed by incubation at 95 °C for 20 min prior to their use in a PCR verification. Supplementary Data 1 summarizes all expression strains generated in this work.

### Sample preparation and UPLC-ESI-HRMS analysis

Cells and XAD16 absorber resin of 50 mL screening cultures were harvested by centrifugation at 3200×*g* for 15 min at 4 °C. Extraction was done 2 × 60 min with 30 mL methanol under stirring at RT. Extracts were filtered using folded filter paper (8–12 µm pore size) and dried using a rotary evaporator. The dried extract was dissolved in 3 mL methanol and analyzed using UPLC-HRMS. An UltiMate 3000 LC System (Dionex) with an Acquity UPLC BEH C-18 column (1.7 μm, 100 × 2 mm; Waters), equipped with a VanGuard BEH C-18 (1.7 µm; Waters) guard column, was coupled to an Apollo II ESI source (Bruker) and hyphenated to maXis 4G ToF mass spectrometer (Bruker). The separation was performed at a flow rate of 0.6 mL min^−1^ (eluent A: deionized water + 0.1% formic acid (FA), eluent B: acetonitrile + 0.1% FA) at 45 °C using the following gradient: 5% B for 30 s, followed by a linear gradient up to 95% B in 18 min and a constant percentage of 95% B for further 2 min. Original conditions were adjusted with 5% B within 30 s and kept constant for 1.5 min. The LC flow was split to 75 µL min^−1^ before entering the mass spectrometer. Mass spectra were acquired in centroid mode ranging from 150 to 2500 *m/z* at a 2 Hz full scan rate. Mass spectrometry source parameters were set to 500 V as endplate offset, 4000 V as capillary voltage, 1 bar nebulizer gas pressure, 5 L min^−1^ dry gas flow, and 200 °C dry temperature. For MS^2^ experiments, CID (collision-induced dissociation) energy was ramped from 35 eV for 500 *m/z* to 45 eV for 1000 *m/z*. MS full scan acquisition rate was set to 2 Hz and MS/MS spectra acquisition rates were ramped from 1 to 4 Hz for precursor ion intensities of 10 kcts to 1000 kcts.

### Quantification of Cys919-1 production

Quantification of Cys919-1 in the heterologous producer *M. xanthus* DK1622 pMYC20Cys_v2 and native producer strain *M. fulvus* SBMx122 was done using an amaZon speed 3D ion trap MS system (Bruker) with an Apollo II ESI source. The LC system, column, and settings as well as the ESI source settings were as described above. We measured Cys919-1 standard solutions with concentrations of 0.001 mg mL^−1^, 0.005 mg mL^−1^, 0.01 mg mL^−1^, 0.05 mg mL^−1^, and 0.1 mg mL^−1^. Solutions for each concentration were prepared three times and measured two times. EIC *m/z* 920.3 [M + H]^+^ peak surface of Cys919-1 were integrated. The mean values of each measurement were used to construct a regression line that was used to calculate the quantity of Cys919-1 in the cultivation extracts.

### Protein overexpression and purification

Standard protocols were used for DNA amplification by PCR, cloning procedures, the transformation of *E. coli*, and plasmid DNA purification after standard alkaline lysis. Genomic DNA was extracted from *C. velatus* Cbv34 with Gentra Puregene DNA Purification Kit (Qiagen). DNA fragments encoding CysJ, CysH, CysH∆AMDH, CysB, CysA, the separate modules 1–4 of CysK and modules 5–6 of CysG were amplified by PCR using the gDNA of Cbv34 as a template and the following primers: CysJ for and CysJ rev, CysH for and CysH rev, CysB for and CysB rev, CysA for and CysA rev, CysK1 for and CysK1 rev 1, CysK2 for 0 and CysK2 rev 1/2, CysK3 for 1/2 and CysK3 rev 1, CysK4 for 1/2 and CysK4 rev, CysG5 for and CysG5 rev 1, CysG6 for 0 and CysG6 rev, respectively. CysH∆AMDH was amplified from pMYC20Cys_v2∆AMDH using CysH for and CysH rev primers.

The amplified DNA fragment encoding CysJ was hydrolyzed with *Nde*I and *Bam*HI and ligated into pET-28b (Novagen) with an N-terminal 6xHis tag. CysH and CysB encoding fragments were hydrolyzed with *Nco*I and *Bam*HI and ligated into pHisSUMOTEV^43^ with an N-terminal 6xHis tag, respectively. The CysA encoding fragment was hydrolyzed with *Nde*I and *Bgl*II and cloned into the second multiple cloning site (MCS) of pETduet-1 (Novagen) generating pETdeut-1-cysA. CysK-M1-4 and CysG-M5-6 encoding fragments were digested with *Bam*HI and *Hind*III and cloned into the first MCS of pETduet-1-cysA to yield N-terminal 6xHis tag and TEV protease site fusion constructs. All generated constructs were Sanger sequenced (LGC Genomics GmbH) to verify that no mutation had been introduced during PCR.

Recombinant protein expression was carried out in *E. coli* BL21 (DE3) grown in LB medium and supplemented with 50 μg mL^−1^ kanamycin or 100 μg mL^−1^ ampicillin. The culture was inoculated with 1/10 volume from a fully-grown overnight culture and cultivated at 37 °C until OD_600_ 0.6–0.9 was reached. The temperature was decreased to 16 °C and after 30 min at 16 °C 0.1 mM isopropyl-β, D-thiogalactopyranoside (IPTG) was supplemented to induce gene expression. The cells were harvested 16 h after induction by centrifugation at 8000×*g* for 10 min at 4 °C and resuspended in lysis buffer (25 mM TRIS (pH 7.5), 150 mM NaCl, 20 mM imidazole). The cells were lysed by the passage at 1500 bar at 4 °C using an M-110P Microfluidizer (microfluidics). The crude extract was centrifuged at 45,000 × *g* for 15 min at 4 °C. The supernatant was treated as a soluble protein fraction.

Affinity chromatography was performed on an Äkta Avant system and size exclusion chromatography (SEC) was performed on an Äkta Pure system. The soluble protein fraction was applied to a 5 mL Ni-NTA cartridge (GE) equilibrated with lysis buffer. The Ni-NTA column was washed with 10 CV (column volumes) lysis buffer prior to elution with elution buffer containing 250 mM imidazole. For CysJ the pooled fractions were concentrated to less than 5 mL and loaded on a HiLoad 16/600 Superdex 200 pg SEC column equilibrated in running buffer (25 mM TRIS (pH 7.5), 150 mM NaCl, 2 mM DTT). For CysH, CysB, CysK-M1-4, and CysG-M5-6 the pooled fractions were applied to a HiPrep 26/10 desalting column equilibrated in running buffer. The resulting fractions were pooled and incubated overnight at 4 °C with TEV protease (1 mg/20 mg protein). After 16 h incubation, 20 mM imidazole was added to the solution prior to loading on a 5 mL Ni-NTA cartridge (GE) equilibrated with lysis buffer. The Ni-NTA column was washed with 10 CV lysis buffer prior to elution with elution buffer containing 250 mM imidazole. The pooled fractions were concentrated to less than 5 ml and loaded on a HiLoad 16/600 Superdex 200 pg SEC column equilibrated in running buffer. Protein purity was determined by SDS-PAGE. Proteins were stored at −80 °C in 25% glycerol.

### Substrate loading experiments

All substrate loading experiments were performed in 50 µL scale using 25 mM TRIS (pH 7.5), 150 mM NaCl, 10 mM MgCl_2_ and 1 mM ATP. For CysH, CysH∆AMDH, CysK-M1-4, and CysG-M5-6, 5 µM protein and 1 mM amino acid were used to test loading of different substrates. Hydroxylation of CysH-bound l-asparagine by CysJ was tested by additionally supplementing 1 mM α-KG, 50 μM FeSO_4_, and 500 nM CysJ. To analyze the transfer of l-asparagine from CysH to CysB, 1 μM CysH was incubated with 1 mM l-asparagine and 5 μM CysB. The transfer of l-asparagine from CysB to CysK-M3 was tested by incubating 5 µM CysK-M3, 1 μM CysH, 500 nM CysB with 1 mM L-asparagine. All reactions were incubated for either 5 min or 2 h at RT, respectively. Direct intact protein UPLC-ESI-MS analysis was done as described below.

### Direct intact protein UPLC-ESI-MS analysis

Direct intact protein UPLC-ESI-MS analysis was performed using an UltiMate 3000 UPLC system coupled with a maXis4G Q-ToF mass spectrometer using an Apollo II ESI source in positive mode. The samples were separated using an Aeris Widepore XB-C8 column (3.6 µm, 150 × 2.1 mm; Phenomenex). The separation was performed at a flow rate of 0.3 mL min^−1^ (eluent A: deionized water + 0.1% FA, eluent B: acetonitrile + 0.1% FA) at 45 °C using the following gradient: 2% B for 30 s, followed by a linear gradient up to 75% B in 10 min and a constant percentage of 75% B for further 3 min. Original conditions were adjusted with 2% B within 30 s and kept constant for 3 min. The LC flow was split to 75 µL min^−1^ before entering the mass spectrometer. Mass spectra were acquired in centroid mode ranging from 150 to 2500 *m/z* at a 2 Hz full scan rate. Mass spectrometry source parameters were set to 500 V as endplate offset, 4000 V as capillary voltage, 1.1 bar nebulizer gas pressure, 6 L min^−1^ dry gas flow, and 180 °C dry temperature. Protein masses were deconvoluted by using the Maximum Entropy deconvolution algorithm in Compass DataAnalysis version 4.4.

### Cysteamine unloading assay and HPLC-MS analysis

To unload CysH-bound l-asparagine or β-hydroxy-l-asparagine, cysteamine was added to a final concentration of 100 mM and incubated at 30 °C for 1 h with slow shaking. The free amines of the unloaded substrate were derivatized with ethyl carbamates prior to HPLC-MS analysis by adding 45 μL ethanol:pyridine (4:1) solution and 5 μL ethyl chloroformate (ECF). After addition of 200 μL deionized water, the derivatized N,N-diethoxycarbonyl β-hydroxyasparaginyl dicysteamine was extracted twice with 300 μL ethyl acetate and 1% ECF. The collected organic layers were dried, dissolved in methanol, and analyzed through HPLC-MS. Chemical synthesis of the di(ethylcarbonyl)asparaginyl-dicysteamine references is described in Supplementary Information.

All measurements were performed using an UltiMate 3000 RSLC system coupled with an amaZon speed 3D ion trap mass spectrometer using an Apollo II ESI source in positive mode. The samples were separated using a BEH C18 column (1.7 μm, 100 × 2.1 mm; waters). The separation was performed at a flow rate of 0.6 mL min^−1^ (eluent A: deionized water + 0.1% FA, eluent B: methanol + 0.1% FA) at 45 °C using the following gradient: starting conditions 5% B for 30 s, linear gradient up to 20% B in 1 min, linear gradient up to 30% B in 13 min, linear gradient up to 95% B in 3 min, constant percentage of 95% B for 3 min, adjusting of original conditions (5% B) in 30 s. The LC flow was split to 75 µL min^−1^ before entering the mass spectrometer. Mass spectra were acquired in centroid mode ranging from 150 to 1500 *m/z*. Mass spectrometry source parameters were set to 500 V as endplate offset, 4000 V as capillary voltage, 1 bar nebulizer gas pressure, 5 L min^−1^ dry gas flow, and 200 °C dry temperature. MS spectra were interpreted using Compass DataAnalysis version 4.4.

### Software

Geneious version 10.1.3 (Biomatters Ltd.) was used to analyze the native cystobactamid BGC sequence and to design the modified BGC and the cloning and expression vector system pMYC in silico. Repetitive sequence segments in *cysK* were analyzed using dot-plot (EMBOSS *6.5.7* tool *dottup*: http://emboss.sourceforge.net/). Chromeleon version 6.80 was used for LC control. HyStar 3.2 Build 49.9 (Bruker) was used for MS scan control. Compass otofControl version 3.4 (Bruker) and trapControl version 8.0 (Bruker) were used for data acquisition of the maXis 4G ToF and the amaZon speed 3D ion trap mass spectrometers, respectively. We used Compass DataAnalysis version 4.4 (Bruker) to interpret MS data and MS Excel 2016 for the calculation of the production titer of Cys919-1. MS PowerPoint 2016 was used for image processing and ChemDraw Professional version 17.1.0.105 (19) was used to create images of chemical structures.

### Reporting summary

Further information on research design is available in the Nature Research Reporting Summary linked to this article.

## Supplementary information

Supplementary Information

Peer Review File

Description of Additional Supplementary Files

Supplementary Data 1–4

Reporting Summary

## Data Availability

Data supporting the findings of this work are available within the paper and its Supplementary Information files. A reporting summary for this article is available as a Supplementary Information file. The datasets and plant materials generated and analyzed during the current study are available from the corresponding author upon request. The sequences of the revised cystobactamid BGC originating from *Cystobacter velatus* Cbv34 (KP836244.2) and the sequences of the modified BGC (MT572315), and the cloning and expression vectors pMYC (MT572316), pMYC20 (MT572317) and pMYC21 (MT572318) have been deposited in the GenBank database. Source data are provided with this paper.

## Source data

Source Data
