# Author Correction: In vivo and in vitro reconstitution of unique key steps in cystobactamid antibiotic biosynthesis

**DOI:** 10.1038/s41467-021-25659-4

**Published:** 2021-09-09

**Authors:** Sebastian Groß, Bastien Schnell, Patrick A. Haack, David Auerbach, Rolf Müller

**Affiliations:** 1 Helmholtz Institute for Pharmaceutical Research Saarland (HIPS), Helmholtz Centre for Infection Research, Saarland University, Campus E8.1, 66123 Saarbrücken, Germany; 2 Department of Pharmacy, Saarland University, 66123 Saarbrücken, Germany; 3 DZIF - German Centre for Infection Research, Partnersite Hannover-Braunschweig, 38124 Braunschweig, Germany

**Keywords:** Expression systems, Multienzyme complexes, Natural products, Antibiotics

Correction to: *Nature Communications* 10.1038/s41467-021-21848-3, published online 16 March 2021.

In the original version of this Article, multiple labels were misplaced in Figure 5b-e and the associated legends. Specifically, “Linker: G” was mixed up with “Linker: F” in Figure 5b and 5c, and the peak label of “Cys889-1c” and “Cys889-2c” in Figure 5d and 5e should be written as “Cys889-1a” and “Cys889-2a”, respectively. Both Figure 5 and its legend have been updated in both the PDF and HTML versions of the Article.

In addition, in the second paragraph of the first section of the Results, Supplementary Figures supporting the statement “UPLC-HRMS analysis and MS^2^ experiments confirmed the heterologous production of 13 unknown and 9 known cystobactamids” were incorrectly cited as “Supplementary Figs. 6–8 and 4”. The correct citation should be “Supplementary Figs. 6–8 and 14”. This has been corrected in both the PDF and HTML versions of the Article.

Furthermore, in the fifth paragraph of the second section of the Results, the sentence, “In the presence of CysH (including the AMDH domain), CysJ, and CysQ, the major products were Cys919-1 and Cys919-2 harboring linkers A and E, respectively”, should be replaced by “In the presence of CysH (including the AMDH domain), CysJ, and CysQ, the major products were Cys919-1 and Cys919-2 harboring linkers E and A, respectively”. Also, in the same section and paragraph “Supplementary Information” supporting the statement “Notably, after deletion of cysJ or the AMDH domain, we also identified four minor unnatural cystobactamids for which we were not able to propose putative structures” should be specified as “Supplementary Method 5”. Thus, the sentence should read “Notably, after deletion of cysJ or the AMDH domain, we also identified four minor unnatural cystobactamids for which we were not able to propose putative structures (Supplementary Method 5; Supplementary Fig. 15)”. These have been corrected in both the PDF and HTML versions of the Article.

