# Supplementary material for: In vivo and in vitro reconstitution of unique key steps in cystobactamid antibiotic biosynthesis
Source: Nat Commun. 2021 Mar 16;12:1696. doi: 10.1038/s41467-021-21848-3 (PMC7966384; doi:10.1038/s41467-021-21848-3)
Supplement: Supplementary file 1 — Supplementary Information [file 41467_2021_21848_MOESM1_ESM.pdf]

In vivo and *in vitro* reconstitution of unique key steps in cystobactamid antibiotic biosynthesis

Groß *et al*.

Supplementary Method 1. ***In silico*** **analysis and revision of the native cystobactamid BGC sequence**

The previously published cystobactamid BGC sequence (GenBank accession number: KP836244) of *C. velatus* Cbv34 includes 52,081 bp with a GC content of 65 %. During the dotplot analysis (*EMBOSS 6.5.7* tool *dottup*: http://emboss.sourceforge.net/) of the BGC we found three repetitive sequence segments in *cysK* (27,422 bp to 41,059 bp) (Supplementary Figure 1). The sequence repeats were identified between modules 1 (C1, A1, T1) and 2 (C2, A2, T2), between modules 1 and 4 (C4, A4, T4), and between modules 2 and 4. Therefore, NRPS modules 1-4 of *cysK* were separately amplified by PCR and re-sequenced using Sanger sequencing (LGC Genomics GmbH). Primer binding sites were located in unique linker regions between the modules to avoid multiple binding in repetitive sequence segments. We used one to two forward and reverse primers for the amplification of the same sequence stretch (two separate PCR reactions per module). We used the primers CysK1 for, CysK1 rev 1, CysK 1 rev 1/2 to amplify module 1, CysK2 for 0, CysK2 rev 1, CysK2 rev 1/2 to amplify module 2, CysK3 for 0, CysK3 rev 1, CysK3 rev 1/2 to amplify module 3 and CysK4 for 0, CysK4 rev, CysK2 rev 1/2 to amplify module 4 (Supplementary Data 3). PCR products were ligated into pETDeut-1 after vector/insert hydrolysis using *Bam*HI and *Hind*III. We found 71 differences compared to the previously published sequence leading to the revision of the sequence. We deposited the revised sequence in GenBank database with accession number KP836244.2.

Supplementary Method 2. ***In silico*** **design of the modified BGC**

The unrevised Cbv34 BGC sequence (GenBank: KP836244) was initially used as template sequence to design the modified BGC. The modified BGC (GenBank accession number MT572315) was organized in two transcriptional units CysOp1 and CysOp2 (Supplementary Figure 2). CysOp1 contains native operon *cysA – N* (without native promoter, RBS of *cysA* and terminator). CysOp2 combines two native operons *cysO – T* and *Orf1 – Orf5*. *Orf1 – Orf5* was engineered downstream of *cysO – T* (native promoters, terminators and RBS of *cysO* excluded; RBS of *Orf5* included). Vanillate-inducible promoter system[1] (P_{van}) was used for inducible gene expression in *M. xanthus* DK1622. P_{van}, including *vanR* encoding repressor gene, was engineered upstream of CysOp1 and only P_{van} (without *vanR*) was added upstream of CysOp2. *tD1* terminator sequence from *M. xanthus* bacteriophage Mx8[2] was engineered downstream of both modified operons, respectively. For transformation-associated recombination (TAR)

assembly, we engineered *LEU2* gene encoding β-isopropylmalate dehydrogenase auxotrophy marker downstream and 100 bp sequences originating from *URA3* gene downstream and upstream of both operons, respectively. We added unique recognition sequences for restriction endonucleases (R-sites) for cloning purposes. Supplementary Table 1 lists all genetic elements including sequence origin and unique R-sites used for the design of CysOp1 and CysOp2. We removed R-sites (e.g. *Bsa*I) by synonymous codon substitutions while keeping the codon usage bias for a single amino acid as similar as possible to the native codon usage bias in the BGC (Supplementary Table 2). To reduce costs and turnaround time for DNA synthesis, we divided the modified BGC into twelve fragments (Supplementary Table 3). We added 100 bp homologous sequences to all adjacent cluster fragments used for TAR cloning. R-sites were engineered at the 5' and 3' ends of all fragments for DNA synthesis vector release or for step-wise assembly in cloning vectors. We flanked some fragments by splitter elements (SEs) if they were used for three-step assembly in cloning vectors.[3] SEs consist of unique 'conventional' type II R-sites flanked by two *Bsa*I recognition sequences extended with 5 bp sequences. *Bsa*I is a type IIS restriction endonuclease cutting outside of the recognition sequence. This allowed generation of variable and unique 5 bp sticky ends for ligation. Unique 'conventional' R-sites allowed stepwise cloning of several fragments into a cloning vector.

Supplementary Method 3. *In silico* design of the cloning and expression vector system pMYC

Three pMYC vector system fragments were designed for TAR assembly of the cystobactamid BGC in yeast, standard cloning procedures in *E. coli* and transformation and integration of the cluster into *M. xanthus* DK1622 genome. The basic pMYC building block contains *p15A* origin of replication (ori) for replication in *E. coli*, *cat* gene encoding chloramphenicol acetyl transferase (mediating resistance towards chloramphenicol), origin of transfer (*oriT*) and transfer gene (*traJ*) for conjugation (not used in this study), *CEN6*/*ARS4* ori for replication in yeast, and *URA3* encoding orotidine 5'-phosphate decarboxylase for counter-selection in yeast. A unique *Eco*RV R-site was located in *URA3* for vector linearization prior to TAR cloning. The flanking 100 bp sequences upstream and downstream of the *Eco*RV R-site are homologous to short sequences added to CysOp1 and CysOp2 to capture the marginal DNA synthesis fragments during TAR cloning. A second pMYC building block (Mx8-tetR) contained the genes *mx8 int* and *tetR*. *mx8 int* encodes *mx8* integrase for integration of plasmids via phage

attachment site (*attP*) in the genome of *M. xanthus* DK1622 or other strains harboring the appropriate *attB* site. The *tetR* gene encodes an efflux transporter mediating resistance towards (oxy)tetracycline. A third pMYC building block (Mx9-kanR) contained genes *mx9 int* and *aph(3')-Ia*. The *mx9 int* gene encodes *Mx9* phage integrase for *M. xanthus* DK1622 genome integration and *aph(3')-Ia* encodes aminoglycoside-3'-phosphotransferase-Ia mediating kanamycin resistance. All genetic elements including unique R-sites and their sequence origin are listed in Supplementary Table 4. Synonymous codon substitutions were performed to remove R-sites from pMYC building blocks (Supplementary Table 5). Supplementary Figure 3 shows vector maps of basic pMYC, pMYC20 (basic pMYC with Mx8-tetR) and pMYC21 (basic pMYC with Mx9-kanR). The sequences were deposited in GenBank database under the following accession numbers: pMYC (MT572316), pMYC20 (MT572317) and pMYC21 (MT572318).

Supplementary Method 4. Assembly of the modified BGC

At the time of sequence revision, we already ordered cluster fragments with sequences based on the previously published *cysK* sequence. Thus, fragments K1, K2 and K3 were completely (and 3-GHIJ5-K partly) synthesized again and named K1_v2, K2_v2, K3_v2 and 3-GHIJ5-K_flong_v2, respectively. 3-GHIJ5-K_flong_v2 contains 1,222 bp of the 3' end of 3-GHIJ5-K (without mutations) and 1,796 bp of pGH DNA synthesis vector, including 559 bp of *ampR* (*bl*a). We used unique R-sites to exchange the mutated part in 3-GHIJ5-K for the generation of 3-GHIJ5-K_v2. The DNA fragments hPvanABCDEF5-G, K123_v2, 3-KLNtD1LEU2h (all part of CysOp1) and ABC3-2345tD1LEU2h (CysOp2) were assembled using pSynbio1[3] as cloning vector. Fragments K123_v2 and 3-KLNtD1LEU2h contained SEs, which were removed by restriction hydrolysis with *Bsa*I and re-ligation (rejoining) using T4 ligase. After rejoining of the fragments, the generated construct did not contain additional R-sites (Supplementary Figure 4). Release of the final fragments from pSynbio1 was achieved by *Bsa*I hydrolysis. Generation of 3-GHIJ5-K_v2 was achieved by ligating 3-GHIJ5-K_flong_v2 into pGH-3-GHIJ5-K. Release of 3-GHIJ5-K_v2, G, hPvanOPQRS and T-ABC15-2 from pGH was achieved by *Bsa*I hydrolysis.

KDummy was generated via PCR using K-rpsLF and K-rpsLR primers pairs (Supplementary Data 3) and pSW1b plasmid as template (unpublished data). pSW1b contains the *rpsL* counter selection marker (mediating streptomycin sensitivity), flanked by *Bsa*I R-sites and 50 bp sequences of *cysK*, which are homologous to the 3' end of 3-GHIJ5-K_v2 and the 5' end of 3-KLNtD1LEU2h. To remove a single point mutation (that we observed after sequence revision) from KDummy, we cloned KDummy into pJET1.2blunt vector (Thermo Fisher Scientific). We used two overlapping, complementary, mutagenic primers KrpsLF_v2_SDM and KrpsLR_v2_SDM to generate pJET1.2KDummy_v2 from pJET1.2KDummy via PCR. Template DNA was hydrolysed with *Dpn*I. KDummy_v2 was released from pJET1.2 using *Xba*I/*Xho*I hydrolysis.

TAR cloning was used to assemble pMYC20preCysOp1_v2 and pMYC21CorOp2. For pMYC20preCysOp1_v2 assembly, DNA fragments hPvanABCDEF5-G, G, 3-GHIJ5-K_v2, KDummy_v2, 3-KLNtD1LEU2h and *Eco*RV-linearized pMYC20 were used. KDummy_v2 was used instead of *cysK* fragments (K123_v2) to avoid unspecific recombination caused by repetitive sequence segments. pMYC21CysOp2 was cloned from fragments hPvanOPQRS, T-ABC15-2, ABC3-2345tD1LEU2h and *Eco*RV-linearized pMYC21. During TAR assembly, the counter selection marker *URA3* in the vectors was disrupted whereas *LEU2* was introduced into the plasmid together with the cluster fragments. Transformation of *S. cerevisiae* ATCC4004247 was done based on the *standard high-efficiency transformation protocol I* described by Agatep *et al*.[4] as described in the method section of the manuscript. Constructed plasmids were isolated as decribed in the method section of the manuscript and transformed into *E. coli* DH10β. Clones harboring the correct construct were verified by restriction analysis after plasmid DNA isolation via standard alkaline lysis.

KDummy_v2 was replaced by K123_v2 in pMYC20preCysOp1_v2 via restriction/ligation to generate pMYC20CysOp1_v2. To generate final expression construct pMYC20Cys_v2, we ligated CysOp2 into pMYC20CysOp1_v2. Supplementary Figure 5 schematically depicts all cloning steps performed to obtain the final expression construct pMYC20Cys_v2. Supplementary Table 6 lists all conventional cloning steps for the generation of pMYC20Cys_v2. We verified all generated constructs by restriction analysis. Additionally, the Illumina paired-end technology on a MiSeq PE300 platform (in-house) was used to verify the

sequences of pMYC20CysOp1_v2 (2,244-fold mean sequencing coverage), pMYC21CysOp2 (2,856-fold) and pMYC20Cys_v2 (1,108-fold). Supplementary Data 1 and Supplementary Data 2 summarize all strains and plasmids generated during cloning process, respectively.

Supplementary Methods 5. Identification of the new cystobactamid derivatives

We identified 22 new cystobactamid derivatives (13 natural and 9 unnatural) based on high-resolution masses and the MS^2 fragmentation patterns. The fragment ions that we used to identify the structural differences of the new derivatives, including the respective calculated fragment masses, are shown in Supplementary Figure 6 and Supplementary Data 4. We only focused on the three fragments a, b and c, because fragment a only contains the N-terminal cystobactamid part (*p*NBA_1-*p*ABA_2), whereas fragment b contains the N-terminal part plus the linker and one additional *p*ABA (*p*NBA_1-*p*ABA_2-linker-*p*ABA_3). Fragment c contains one additional (tailored) *p*ABA (*p*NBA_1-*p*ABA_2-linker-*p*ABA_3-*p*ABA_4) compared to fragment b. Those three fragments were also described previously by Baumann and coworkers (in the SI).[5] We decided to look at those three fragments, because it enabled us to find structural differences in the N-terminal part, e.g. after deletion of *cysR* (*m/z* 269.0562 → 239.0821 $[M+H]^+$; NO_2 → NH_2), in the linker, e.g. after deletion of *cysQ* (*m/z* 532.1468 → 518.1312 $[M+H]^+$; methoxy-L-(iso)Asn → hydroxy-L-(iso)Asn), and in the C-terminal part, e.g. *m/z* 725.2207 → 711.2051 $[M+H]^+$; isopropoxyl-pABA_4 → ethoxy-pABA_4).

The differences between experimental masses and the calculated masses of the new derivatives (and their corresponding fragments) are listed in Supplementary Data 4. Based on the high-resolution mass of the new derivatives and their fragmentation pattern we were able to propose the structures (Supplementary Figure 7) using known cystobactamids as template (example depicted in Supplementary Figure 14).

E.g. after the deletion of *cysQ*, the gene encoding the *O*-methyltransferase that acts on the linker, and heterologous expression of the deletion construct in *M. xanthus* DK1622, we did not find the two major cystobactamid peaks (Cys919-1 and Cys919-2) in the EIC with *m/z* 920.3 $[M+H]^+$. Instead we found two new masses with *m/z* 906.3004 (Cys905-1c) and 906.3007 (Cys905-2c) $[M+H]^+$ (red box in Supplementary Figure 14). The fragmentation pattern shows that fragment a (blue box), which is the N-terminal cystobactamid part (pNBA_1-pABA_2) of

Cys905-1c/2c is the same as for Cys919-1/2. However, fragment b (green box) differs when comparing Cys919 with Cys905, as there is a mass shift of 14 Da (*m/z* 532.1468 → 518.1312 $[M+H]^+$), indicating that a methyl group is missing in Cys905 (structural difference shown in red). In fragment c (yellow box) and in the mass of the molecular ion (red box) the mass shift of 14 Da is still present, which means that no further structural difference is found between Cys919 and Cys905. Assignment of the linker moieties with the same masses (e.g. linker G and F for Cys905-1c and Cys905-2c, respectively) was done based on the different retention times that were also observed for previously reported cystobactamids, in which derivatives harboring L-isoasparagine linkers eluted between 1.4 and 1.6 min earlier than derivatives harboring L-asparagine linkers.

However, for four unnatural cystobactamids, we were not able to propose a putative structure: Cys905-2d, Cys905-2e, Cys919-2b_1 and Cys919-2b_2. In *M. xanthus* DK1622 pMYC20Cys_v4Δ*cysJ* and *M. xanthus* DK1622 pMYC20Cys_v4Δ*cysJ*ΔAMDH we found the two new minor Cys905 derivatives, Cys905-2d and Cys905-2e (Supplementary Figure 15). The retention times are different to the one observed for Cys905-1c/2c (major products in the extract of *M. xanthus* DK1622 pMYC20Cys_v4Δ*cysQ*), but surprisingly, the fragmentation pattern is the same (see Supplementary Data 4). Since we found Cys905-2d and Cys905-2e in the extracts of the *cysJ* and the *cysJ*-AMDH deletion strains, we speculate that the linker is an L-asparagine rather than an L-isoasparagine derivative, because L-isoasparagine linkers only occur if the AMDH domain is present. In the *cysJ* deletion strains we expected the production of exclusively desmethoxylated linker derivatives (fragment b: *m/z* 502.1363 $[M+H]^+$). However, the fragment ion b has an *m/z* of 518.1312 $[M+H]^+$, which means that fragment b potentially contains an additional hydroxyl group even though *cysJ* was deleted in the producer strains. Since the retention times are different compared to Cys905-1c/2c, we can only hypothesize that the hydroxylation occurs in a different position and that it might be catalyzed by a different enzyme encoded in the genome of *M. xanthus* DK1622. However, this enzyme must be active also in the other strains harboring different construct. Thus, we searched for cystobactamid derivatives with double-hydroxylated fragment b ions, but we did not find any of them. Finally, purification and subsequent NMR analysis would be required to understand where the hydroxylation in Cys905-2d/e occurs and why those two derivatives have a different retention time despite the same potential linker moiety. However, due to the very low production titer, this is not possible at this time.

Furthermore, we found two new Cys919 derivatives, Cys919-2b$_1$ and Cys919-2b$_2$ (Supplementary Figure 15), in the extract of the AMDH deletion strain. Both derivatives show the same fragmentation pattern compared to each other, but they have a different fragmentation pattern compared to Cys919-2 (see Supplementary Data 4). The fragmentation pattern shows that the linker is desmethylated (fragment b: *m/z* 518 $[M+H]^+$ compared to *m/z* 532 $[M+H]^+$ for Cys919-2), because CysQ is not able to operate in case the AMDH domain is deleted. Since fragment c has a difference of 14 Da compared to fragment c of Cys919-2, the additional methyl group has to be located in the terminal pABA$_5$ moiety. We suggest that R$_2$ (see Supplementary Figure 6 and Supplementary Data 4) is a 1-methylpropoxy moiety as this moiety has already been described in native cystobactamids. However, since we identified two derivatives with only slightly different retention times (0.22 min), the methyl group might also be located elsewhere or at different positions in Cys919-2b$_1$ and Cys919-2b$_2$. We also cannot exclude the possibility that there is another structural difference elsewhere in the molecule, which we cannot explain at this time and only based on MS^2 data. As for Cys905-2d/e, we would also have to isolate those derivatives, which is not possible due to the very low production titers.

Supplementary Method 6. Analysis of CysJ activity on free L-asparagine using TLC

To test if free L-asparagine is hydroxylated by CysJ, 500 nM CysJ was incubated with 1 mM α-KG and 1 mM L-asparagine and analyzed using thin-layer chromatography (TLC) with ninhydrine (Supplementary Figure 16 lane 5). We observed different colors for L-aspartate (lane 2; control) and L-isoasparagine (lane 3; control), respectively, and a different retention factor of L-isoasparagine compared to L-asparagine (lane 1; control). The incubation of L-asparagine with CysJ and α-KG (lane 5) prior to TLC analysis did not result in visible turnover or changing retention factors compared to the L-asparagine control, L-asparagine incubated with CysJ without α-KG (lane 4), or L-asparagine incubated with denaturated CysJ and α-KG (lane 6).

Supplementary Method 7. Synthesis of the di(ethylcarbonyl)asparaginyl-dicysteamine references

Supplementary Figure 17 depicts all below mentioned reaction steps for the synthesis of the di(ethylcarbonyl)asparaginyl-dicysteamine references. 55.6 mg Trityl chloride (0.2 mmol, 2eq.), 19 µL acetic anhydride (0.2 mmol, 2eq.) and 6.1 µL concentrated H_2SO_4 (0.115 mmol,

1.15eq.) are added to 500 µL glacial acetic acid until dissolution. 35.4 mg L-isoasparagine (0.1 mmol, 1eq.) and 50 µL DMF (dimethylformamide) are added and left to react overnight at 60°C in an oil bath. The solution was slowly poured on 2 mL ice cold H_2O, the pH was adjusted to 6.0 by addition of 10 M NaOH and left on ice for 1 h. The precipitate was filtrated using silica gel, washed with water and subsequently dissolved in DCM (dichloromethane). 37.4 mg of Trityl-L-isoasparagine (100 µmol, 1eq.) and 27.9 µL TEA (trimethylamine; 200 µmol, 2eq.) were dissolved in 500 µL THF (tetrahydrofuran) and 34.5 µL di-tert-butyl dicarbonate were added and left to react at room temperature for 2 h. Upon completion the reaction was quenched in water, the pH was acidified by addition of 1 M HCl and the boc-L-isoasparagine(trt)-OH was extracted with DCM. 50 mg boc-L-asparagine (trt)-OH or boc-L-isoasparagine (trt)-OH (105 µmol, 1eq.), 35.2 µL DIPEA (N,N-diisopropylethylamine; 367 µmol, 3.5 eq.) and 60.1 mg PyBop (115 µmol, 1.1 eq.) were dissolved in 2 mL DCM and stirred at room temperature for 30 min. 29.8 mg cysteamine hydrochloride (262 µmol, 2.5eq.) and 1 mL DMSO were added and incubated overnight at room temperature. The reaction was quenched in H_2O and the product was extracted twice with DCM. The organic fractions were dried, and the white precipitate was washed with pentane. The obtained powder was left to react at room temperature overnight in 2 mL of an 88 % TFA, 5 % phenol, 5 % H_2O, 2 % TIPS (triisopropylsilane) solution with addition of 2 eq. of cysteamine to prevent thiol disulfide exchange. The reaction was quenched in water, washed three times with DCM and the aqueous layer was lyophilized to yield pure asparaginyl-dicysteamine. The compound was dissolved in ethanol and left to react with 5% ECF before HPLC-MS analysis.

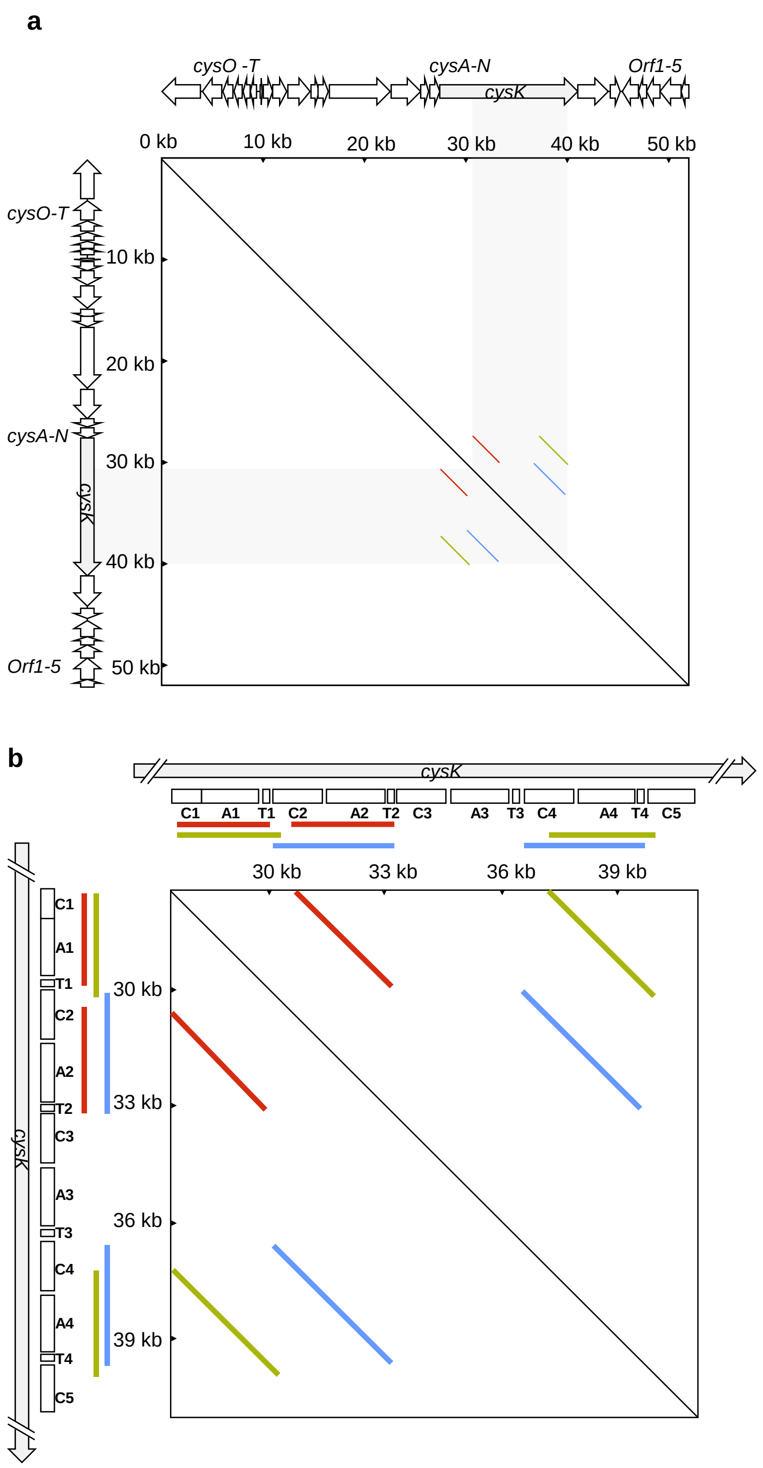

Supplementary Figure 1. Dotplots of the entire cystobactamid gene cluster and of *cysK*. a: Dotplot of the entire cystobactamid BGC sequence. Three repetitive sequence segments (red, blue and green lines) span from about 30 kb to 40 kb (highlighted in light grey) in *cysK*. **b:** Dotplot of *cysK*. Magnification of the grey area in a. C: condensation domain, A: adenylation domain, T: thiolation domain. The Dotplot was created using the *EMBOSS 6.5.7 tool dottup* in Geneious. Source data are provided as a Source Data file.

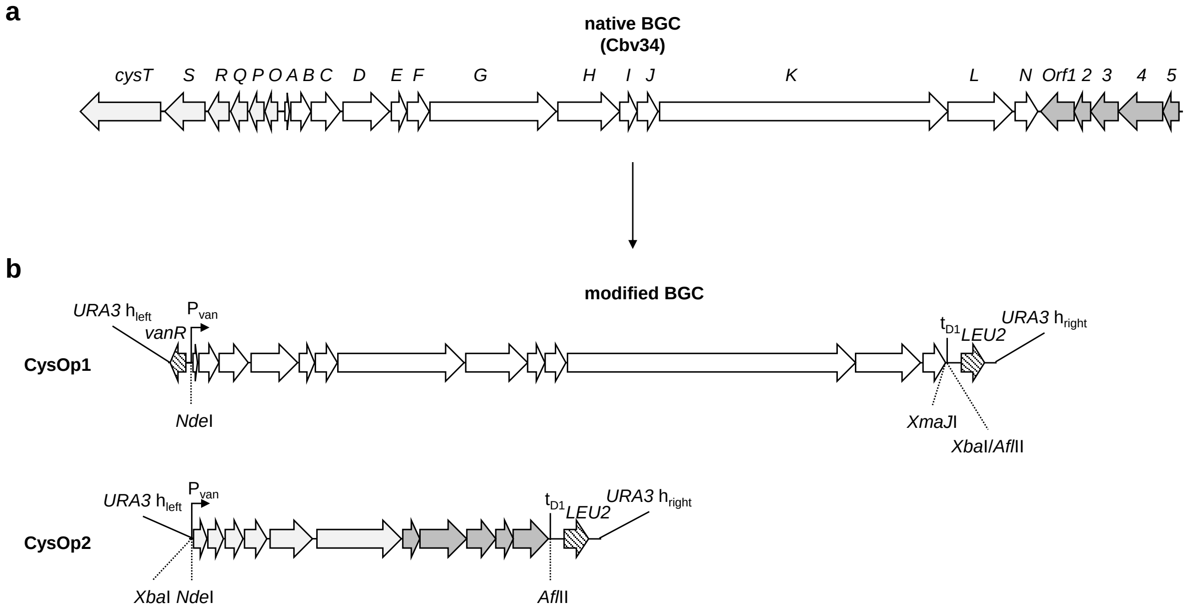

Supplementary Figure 2. Design of the modified BGC based on the native BGC. The modified BGC (**b**) was designed based on the native BGC (**a**) consists of two operons: CysOp1: *URA3* homology left for TAR assembly, *vanR* repressor, vanillate inducible promoter Pvan, biosynthetic genes *cysA–N*, tD1 terminator, *LEU2* auxotrophy marker and *URA3* homology right for TAR assembly. CysOp2: *URA3* homology left, Pvan, biosynthetic genes *cysO–T* and *Orf1-5*, tD1, *LEU2* and *URA3* homology right. Location of important R-sites are shown by dashed lines.

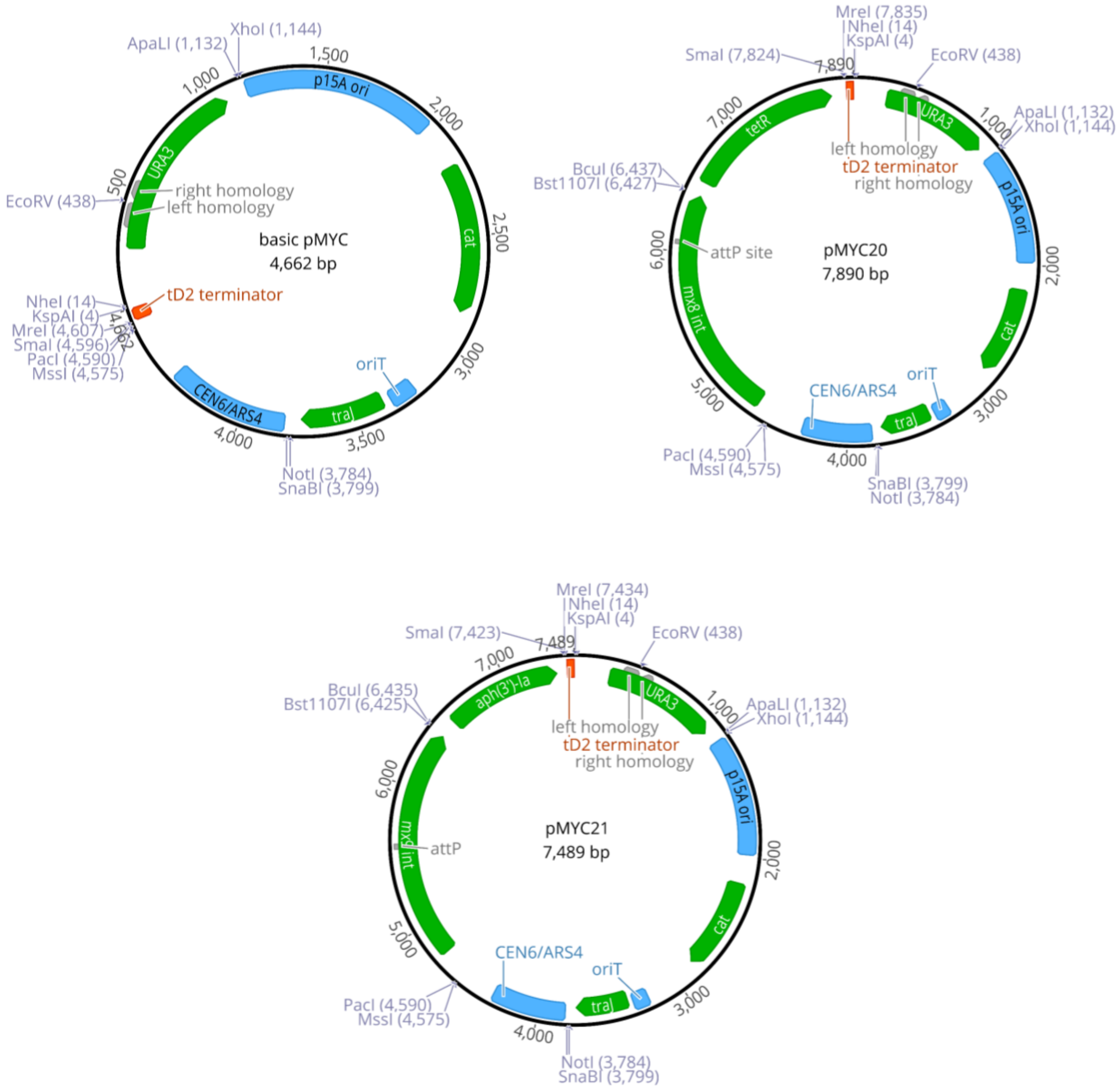

Supplementary Figure 3. Plasmid maps of basic pMYC, pMYC20 and pMYC21. Mx8-tetR building block engineered into basic pMYC results in pMYC20. Likewise, Mx9-kanR building block and basic pMYC generate pMYC21. Genes are annotated using green arrows. Origins of replication or transfer are annotated using blue boxes. Terminator sequence is labeled using red box and homology sequences for TAR cloning are shown in grey. Important unique R-sites are shown in dark blue. Vector maps were generated using *Geneious* v10.1.3. Source data are provided as a Source Data file.

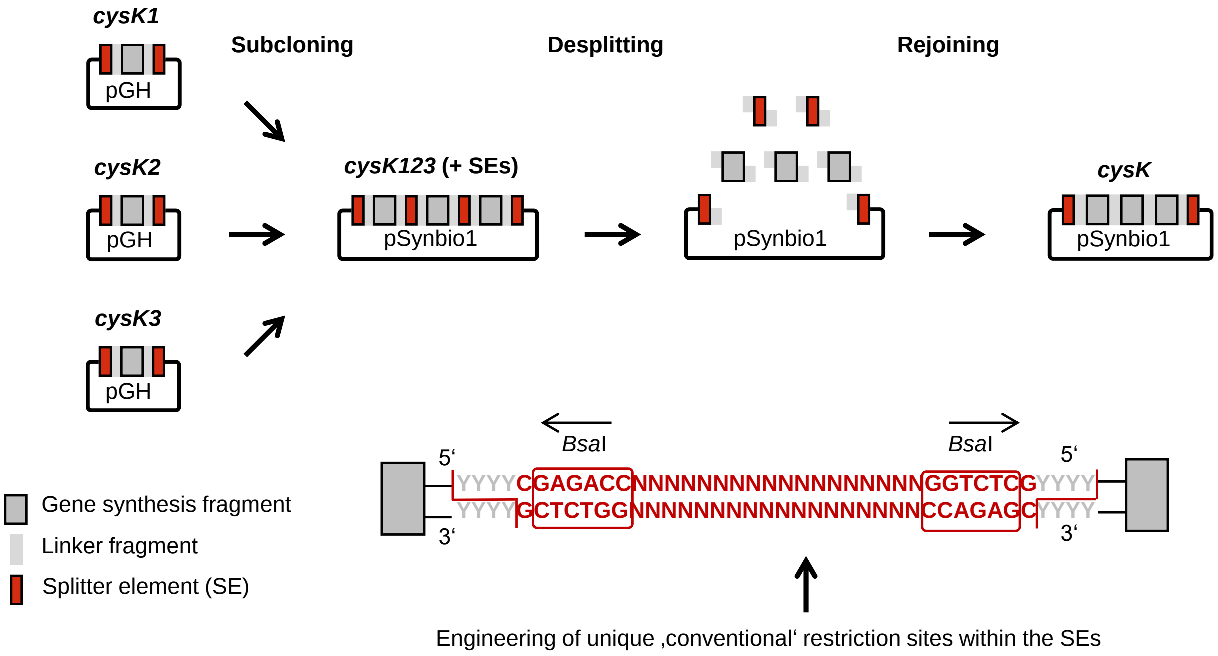

Supplementary Figure 4. Scheme for the three-step assembly strategy using splitter elements (SEs) on the example of *cysK*. a: Gene synthesis fragments were released from the synthesis vectors and stepwise ligated in the cloning vector pSynbio1 (subcloning). Desplitting was performed using *Bsa*I type IIS restriction enzyme. Rejoining of the fragments occurred after removal of splitter elements (SEs). Grey box: gene synthesis fragment; light-grey box: linker fragment; red box: SE. **b:** organization of a SE.

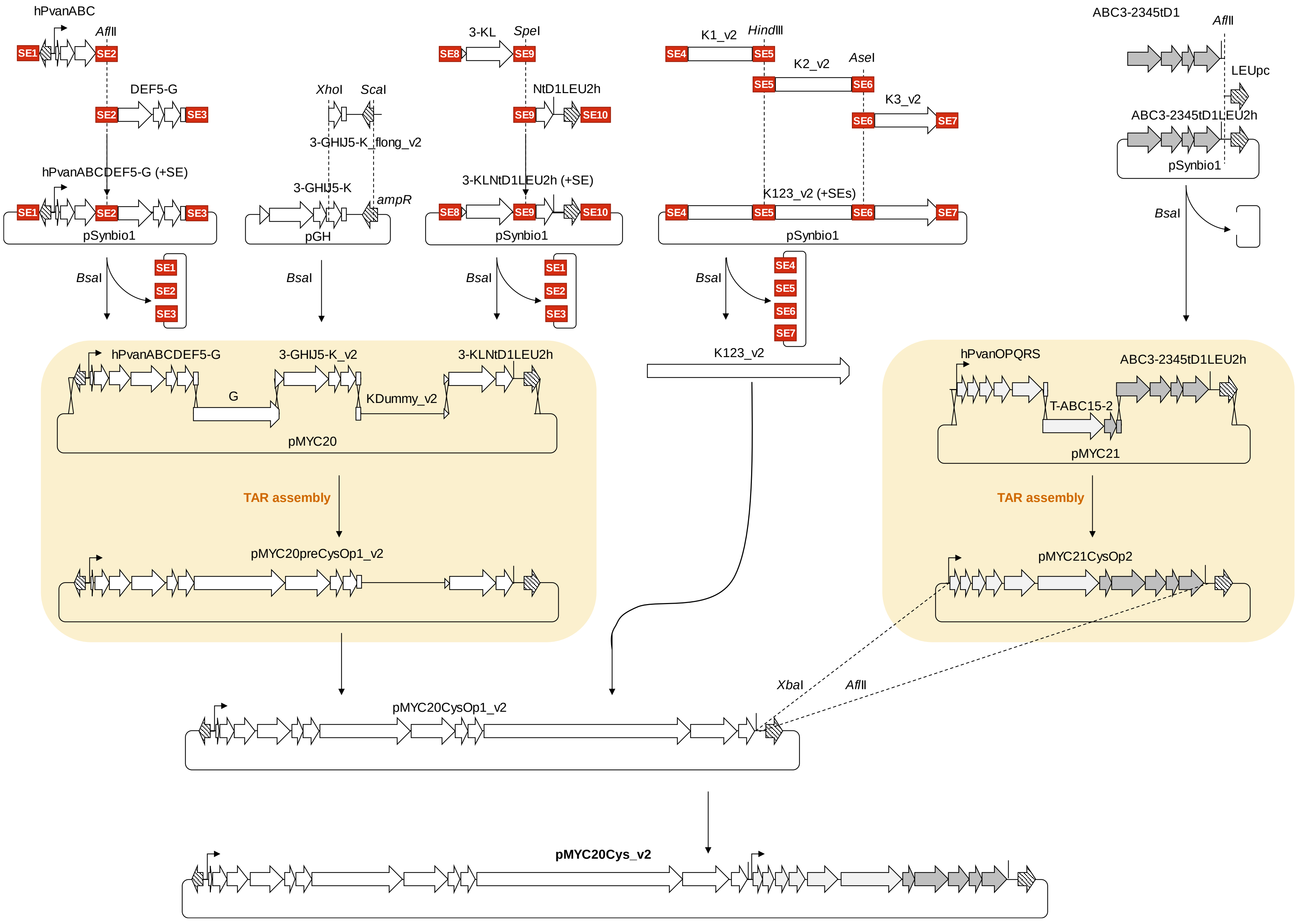
hPvanABC
AflII
SE1
SE2
DEF5-G
SE2
SE3
hPvanABCDEF5-G (+SE)
SE1
SE2
SE3
pSynbio1
XhoI
ScaI
3-GHIJ5-K_flong_v2
3-GHIJ5-K
ampR
pGH
3-KL
SpeI
SE8
SE9
NtD1LEU2h
SE9
SE10
3-KLNtD1LEU2h (+SE)
SE8
SE9
SE10
pSynbio1
K1_v2
HindIII
SE4
SE5
K2_v2
AseI
SE5
SE6
K3_v2
SE6
SE7
K123_v2 (+SEs)
SE4
SE5
SE6
SE7
pSynbio1
ABC3-2345tD1
AflII
LEUpc
ABC3-2345tD1LEU2h
pSynbio1
BsaI
BsaI
BsaI
BsaI
BsaI
SE1
SE2
SE3
SE1
SE2
SE3
SE4
SE5
SE6
SE7
hPvanABCDEF5-G
3-GHIJ5-K_v2
3-KLNtD1LEU2h
G
KDummy_v2
pMYC20
TAR assembly
pMYC20preCysOp1_v2
K123_v2
hPvanOPQRS
ABC3-2345tD1LEU2h
T-ABC15-2
pMYC21
TAR assembly
pMYC21CysOp2
XbaI
AflII
pMYC20CysOp1_v2
pMYC20Cys_v2

Supplementary Figure 5. Assembly strategy of the modified gene cluster. Both operons of the modified gene cluster are shown on top (see also Supplementary Figure 2). Conventional restriction/ligation-based cloning steps were used to assemble hPvanABCDEF5-G, 3-GHIJ5-K_v2, 3-KLNtD1LEU2h, K123 (CysOp1) and ABC3-2345tD1LEU2h (CysOp2) from synthesized DNA fragments. Splitter elements (SEs; shown in red) were used for stepwise cloning into pSynbio1 (depicted in Supplementary Figure 4). TAR cloning (highlighted in beige) was used to assemble fragments hPvanABCDEF5-G, G, 3-GHIJ5-K_v2, KDummy, 3-KLNtD1LEU2h and pMYC20 to generate pMYC20preCysOp1_v2. Likewise, pMYC21CysOp2 was assembled via TAR from hPvanOPQRS, T-ABC15-2, ABC3-2345tD1LEU2h and pMYC21. KDummy was replaced by K123 in a restriction/ligation-based cloning step to generate pMYC20CysOp1_v2. Final expression construct pMYC20Cys_v2 was generated by cloning CysOp2 downstream of CysOp1 in pMYC20CysOp1_v2. Supplementary Table 6 lists all restriction/ligation-based cloning steps.

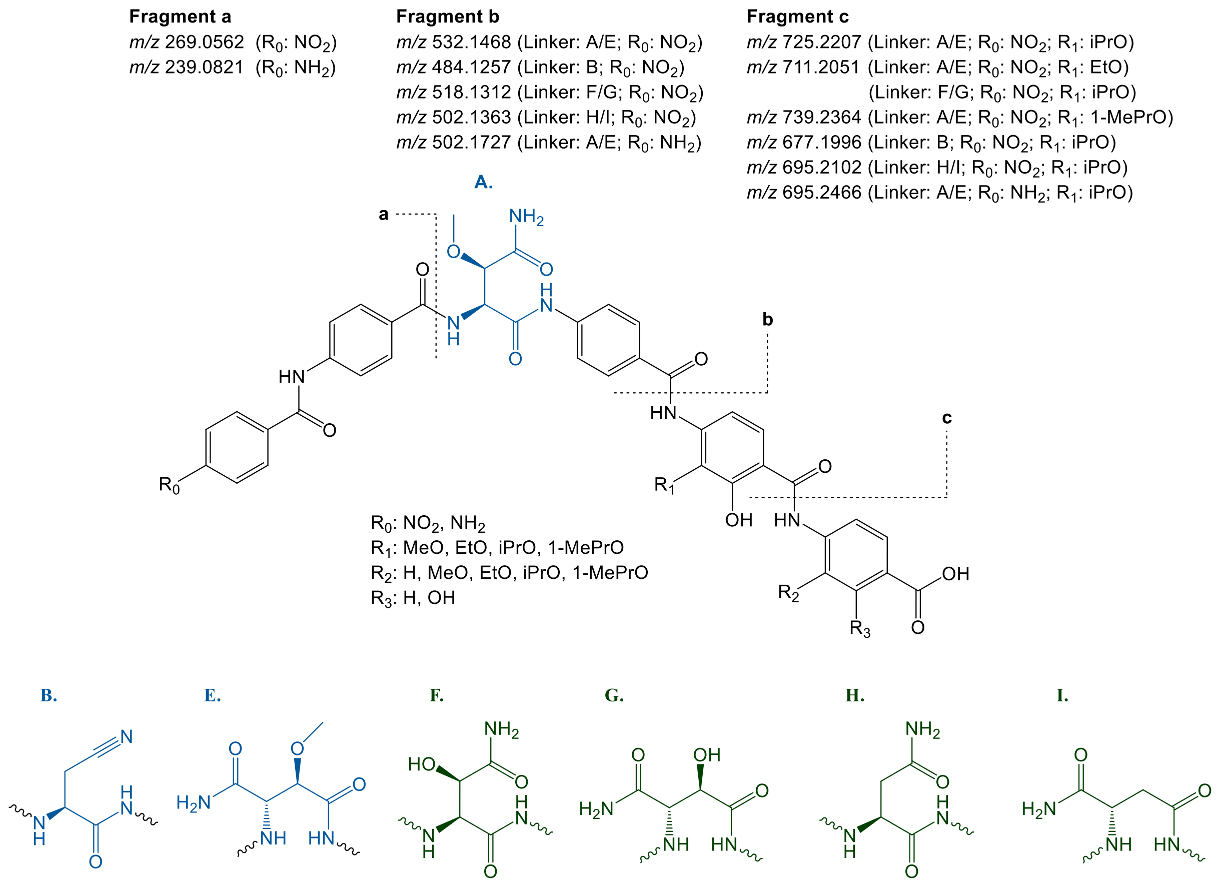

Supplementary Figure 6. Fragmentation scheme of natural and unnatural cystobactamids. Fragments a, b and c were used in MS^2 fragmentation experiments to identify novel natural and unnatural cystobactamids (Table 1 and Supplementary Data 4). The calculated m/z $[M+H]^+$ values of the fragments depend on tailoring of $R_{0\text{-}3}$ and linker moiety.

a

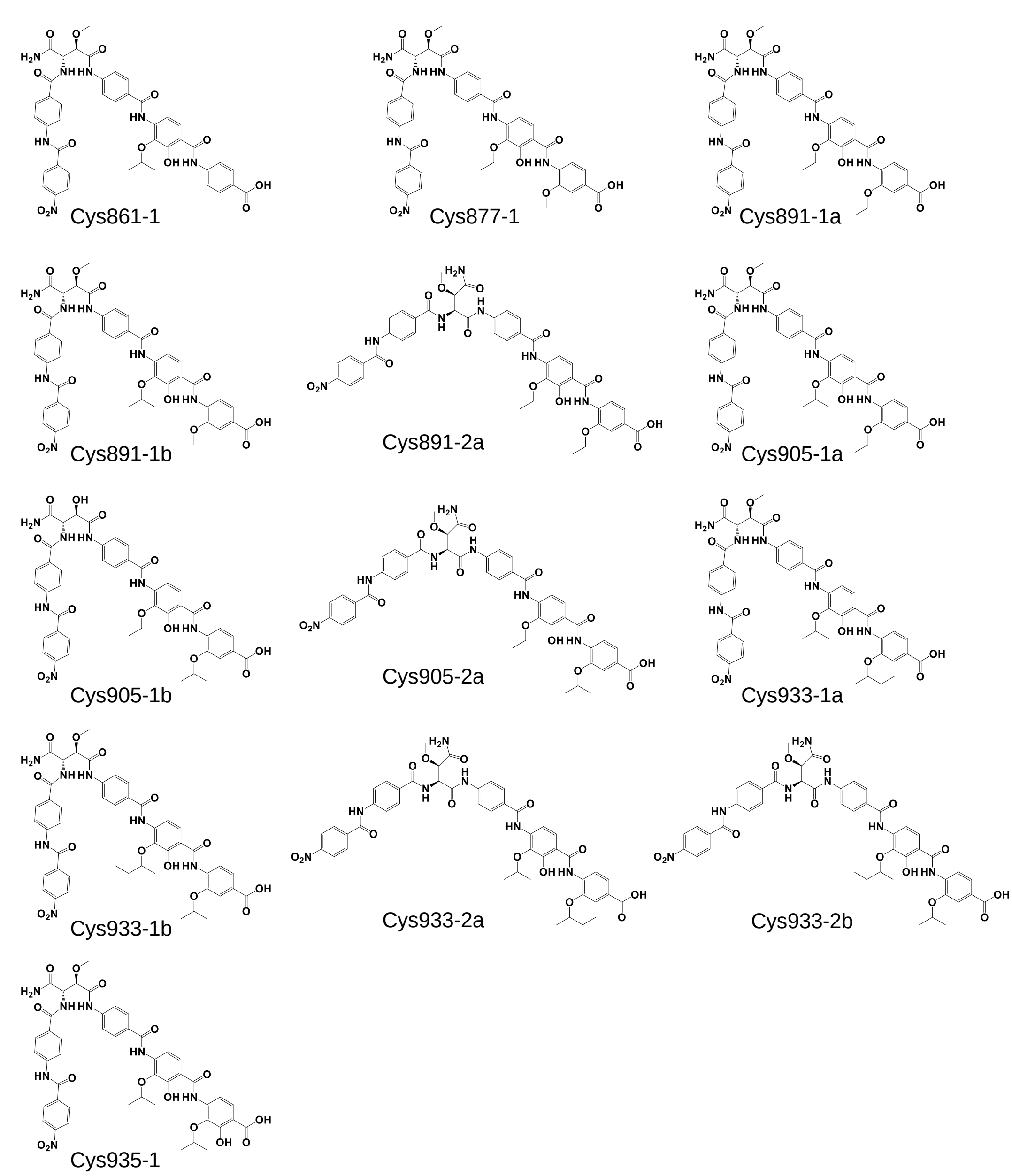

b

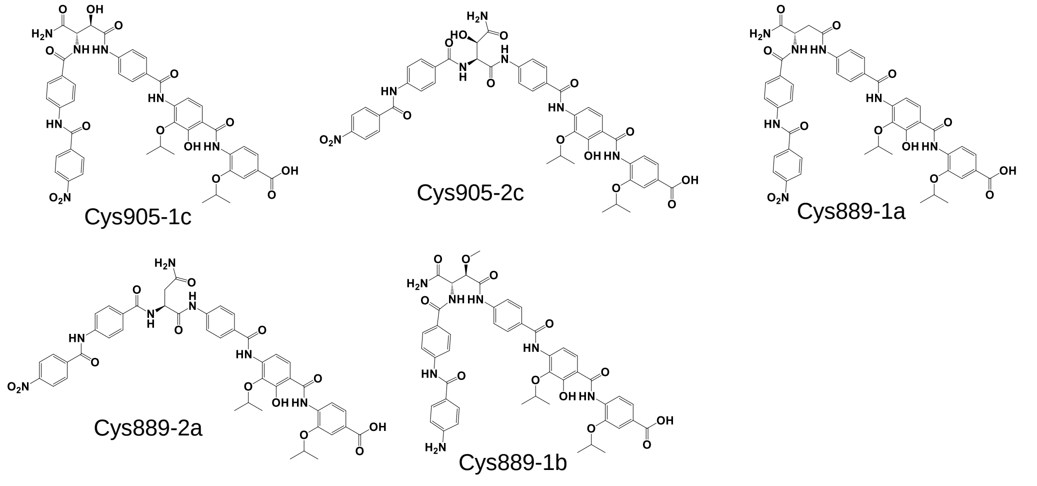

Supplementary Figure 7. Proposed structures of novel natural and unnatural cystobactamids. Natural (**a**) and unnatural (**b**) cystobactamid structures were proposed based on exact mass from HRMS analysis and MS^2 fragmentation.

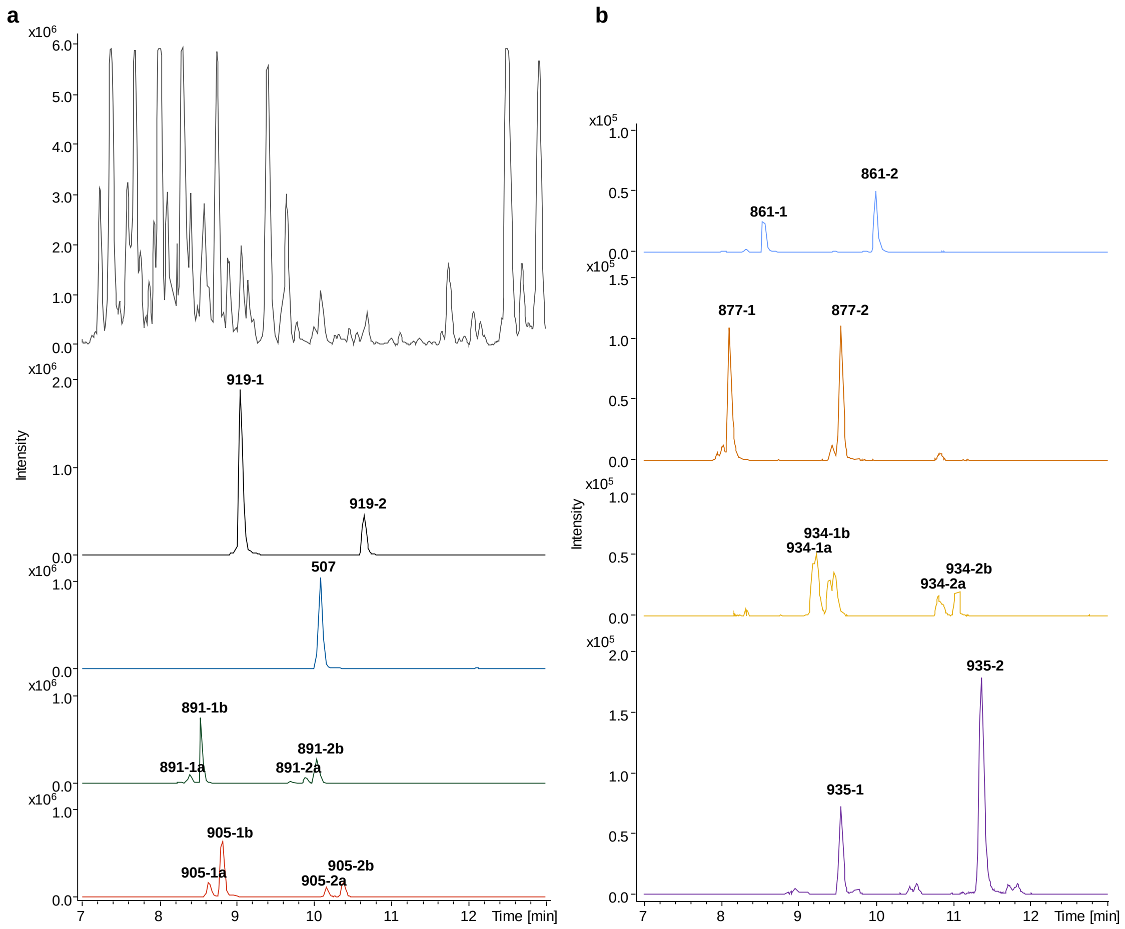

Supplementary Figure 8. HPLC-MS analysis of major and minor cystobactamid derivatives produced by *M. xanthus* DK1622 pMYC20Cys_v2. a: BPC (dark grey), EIC 920.30 (black), EIC 508.20 (dark blue), EIC 892.27 (dark green) and EIC 906.29 (red) (all EICs $[M+H]^+$ ± 0.02 Da) show existence of Cys919-1/2, Cys507, Cys891-1a/1b/2a/2b and Cys9051a/1b/2a/2b. **b:** BPC and EICs of minor native cystobactamid derivatives in methanolic *M. xanthus* DK1622 pMYC20Cys_v2 extracts. EIC 862.26 (blue), EIC 878.26 (dark orange), EIC 935.30 (red) and EIC 936.30 (purple) (all EICs $[M+H]^+$ ± 0.02 Da) show existence of Cys861-1/2, Cys877-1/2, Cys934-1a/1b/2a/2b and Cys935-1/2. Corresponding MS^2 data are listed in Supplementary Data 4.

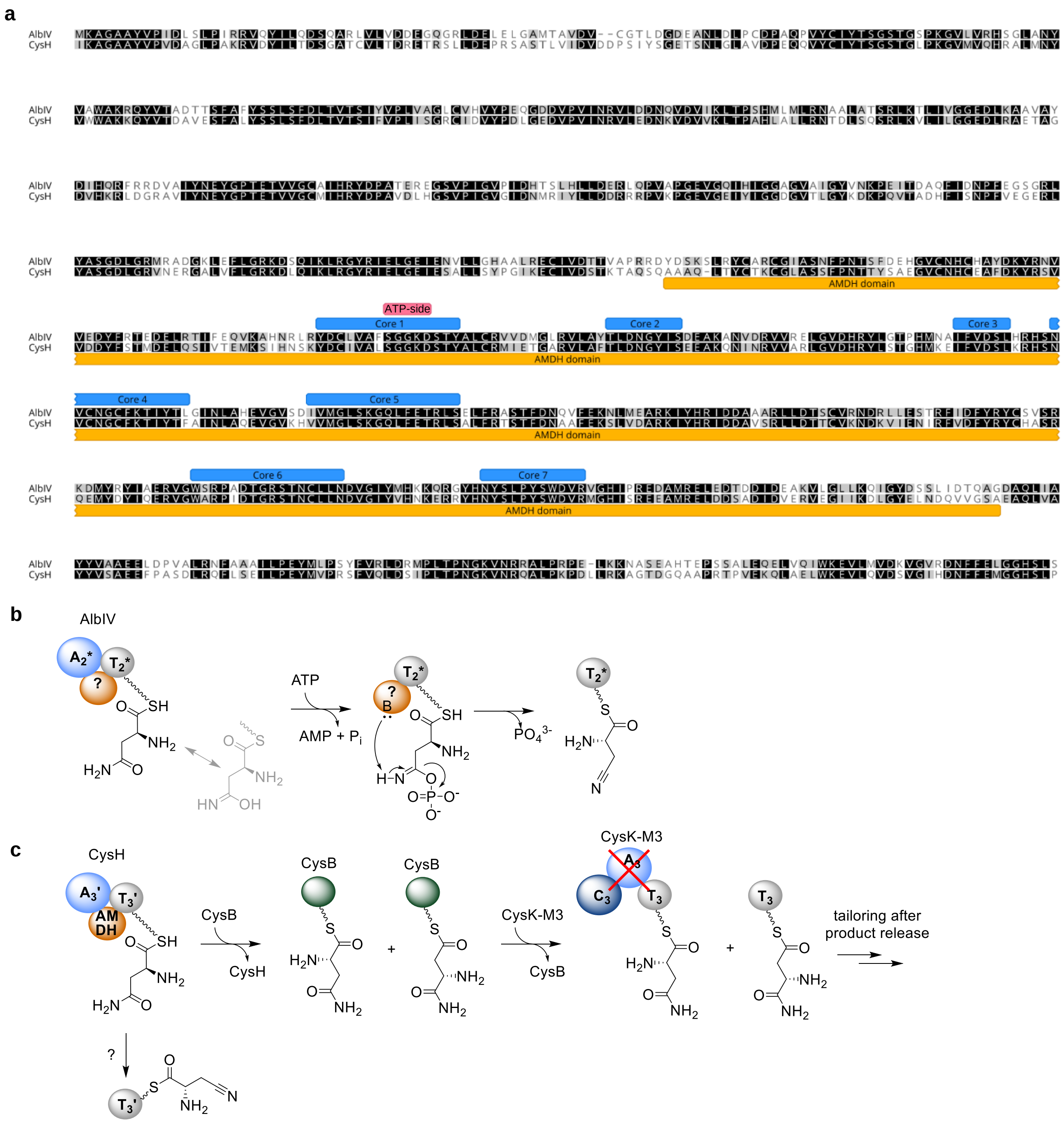

Supplementary Figure 9. Alignment of AlbIV and CysH and previously proposed linker biosynthesis pathways. a: MUSCLE[6] (version 3.8.425) alignment of AlbIV and CysH protein sequences (created using *Geneious* version 2020.0). The AMDH domain is labelled in orange, the ATP-binding motif (SGGKD) is colored in pink and putative core motifs identified by comparison with 25 closest homologs (see Supplementary Figure 10) are labelled in blue. **b:** Proposed reaction mechanism in albicidin linker biosynthesis for the generation of β-cyano-L-alanine (modified from [7]). **c:** Proposed cystobactamid linker biosynthesis for the generation of L-asparagine and L-isoasparagine. No biosynthesis pathway for β-cyano-L-alanine was proposed in cystobactamid linker biosynthesis so far. Source data underlying Supplementary Figure 9a are provided as a Source Data file.

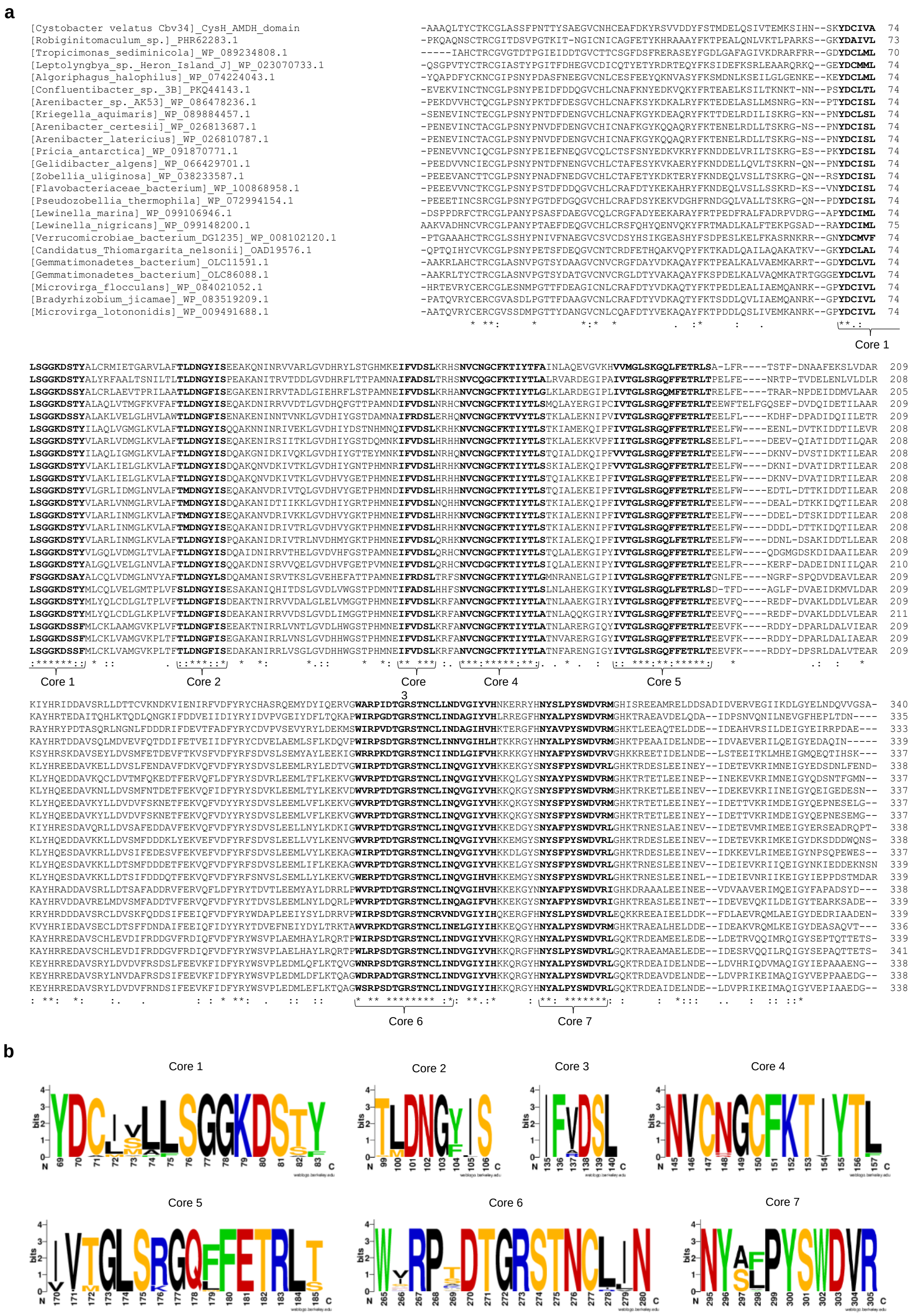

Supplementary Figure 10. Comparison of the AMDH domain with 25 closest homologs. a: Sequence alignment of the AMDH domain with 25 closest BLAST homologs. **b:** Sequence logo of the seven core motifs (generated using https://weblogo.berkeley.edu/)[8]. Core motif 1 harbors a ATP-binding motig (SGGKD) that was also found in the unusual domain in AlbIV.[7] Source data underlying Supplementary Figure 10a are provided as a Source Data file.

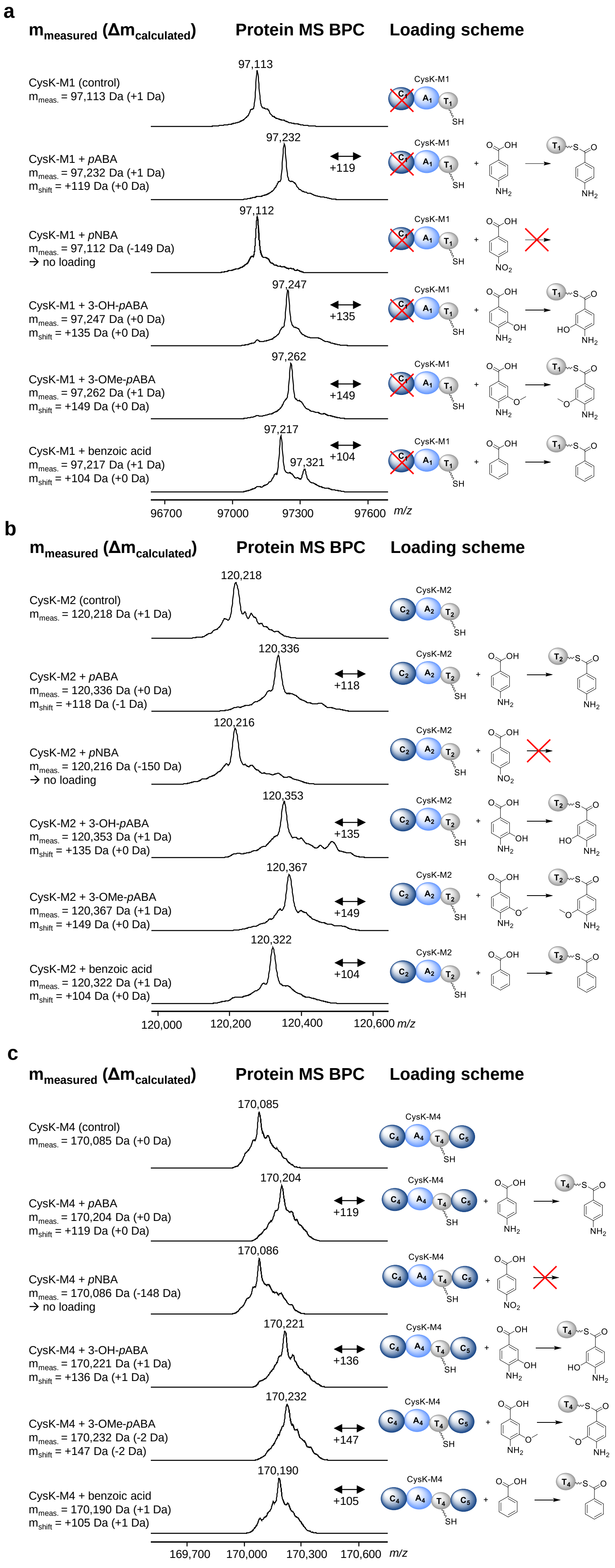
a
mmeasured (Δmcalculated)
Protein MS BPC
Loading scheme
CysK-M1 (control)
mmeas. = 97,113 Da (+1 Da)
97,113
CysK-M1 + pABA
mmeas. = 97,232 Da (+1 Da)
mshift = +119 Da (+0 Da)
97,232
+119
CysK-M1 + pNBA
mmeas. = 97,112 Da (-149 Da)
→ no loading
97,112
CysK-M1 + 3-OH-pABA
mmeas. = 97,247 Da (+0 Da)
mshift = +135 Da (+0 Da)
97,247
+135
CysK-M1 + 3-OMe-pABA
mmeas. = 97,262 Da (+1 Da)
mshift = +149 Da (+0 Da)
97,262
+149
CysK-M1 + benzoic acid
mmeas. = 97,217 Da (+1 Da)
mshift = +104 Da (+0 Da)
97,217
97,321
+104
96700
97000
97300
97600
m/z
b
mmeasured (Δmcalculated)
Protein MS BPC
Loading scheme
CysK-M2 (control)
mmeas. = 120,218 Da (+1 Da)
120,218
CysK-M2 + pABA
mmeas. = 120,336 Da (+0 Da)
mshift = +118 Da (-1 Da)
120,336
+118
CysK-M2 + pNBA
mmeas. = 120,216 Da (-150 Da)
→ no loading
120,216
CysK-M2 + 3-OH-pABA
mmeas. = 120,353 Da (+1 Da)
mshift = +135 Da (+0 Da)
120,353
+135
CysK-M2 + 3-OMe-pABA
mmeas. = 120,367 Da (+1 Da)
mshift = +149 Da (+0 Da)
120,367
+149
CysK-M2 + benzoic acid
mmeas. = 120,322 Da (+1 Da)
mshift = +104 Da (+0 Da)
120,322
+104
120,000
120,200
120,400
120,600
m/z
c
mmeasured (Δmcalculated)
Protein MS BPC
Loading scheme
CysK-M4 (control)
mmeas. = 170,085 Da (+0 Da)
170,085
CysK-M4 + pABA
mmeas. = 170,204 Da (+0 Da)
mshift = +119 Da (+0 Da)
170,204
+119
CysK-M4 + pNBA
mmeas. = 170,086 Da (-148 Da)
→ no loading
170,086
CysK-M4 + 3-OH-pABA
mmeas. = 170,221 Da (+1 Da)
mshift = +136 Da (+1 Da)
170,221
+136
CysK-M4 + 3-OMe-pABA
mmeas. = 170,232 Da (-2 Da)
mshift = +147 Da (-2 Da)
170,232
+147
CysK-M4 + benzoic acid
mmeas. = 170,190 Da (+1 Da)
mshift = +105 Da (+1 Da)
170,190
+105
169,700
170,000
170,300
170,600
m/z
CysK-M1
CysK-M2
CysK-M4

Supplementary Figure 11. Substrate specificity of CysK-M1, CysK-M2 and CysK-M4. Observed mass shifts in deconvoluted protein MS BPCs reveal loading of *p*ABA, 3-OH-*p*ABA, 3-OMe-*p*ABA and benzoic acid but not of *p*NBA by CysK modules 1 (**a**), 2 (**b**) and 4 (**c**), respectively. Blue sphere: adenylation domain; grey sphere: thiolation domain; red cross: no substrate loading observed.

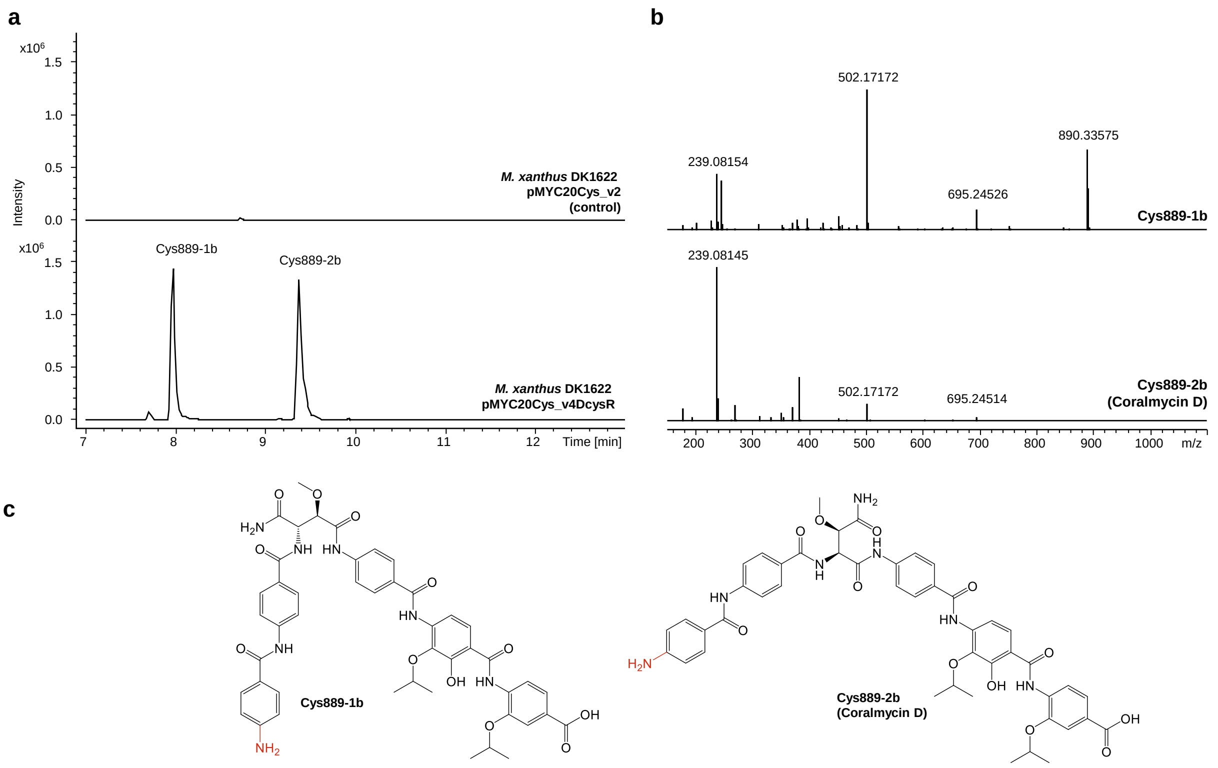

Supplementary Figure 12. UPLC-HRMS data and structures of Cys889-1b/2b produced in *M. xanthus* DK1622 pMYC20Cys_v4ΔcysR. a: EIC *m/z* 890.29 $[M+H]^+$. **b:** MS^2 spectra of Cys889-1b/2b. Fragment with *m/z 239* Da $[M+H]^+$ (30 Da mass shift to natural cystobactamids) confirms that nitro group is replaced by amide group in N-terminal *p*ABA. **c:** structure proposal of Cys889-1b and Cys889-2b (Coralmycin D)[9].

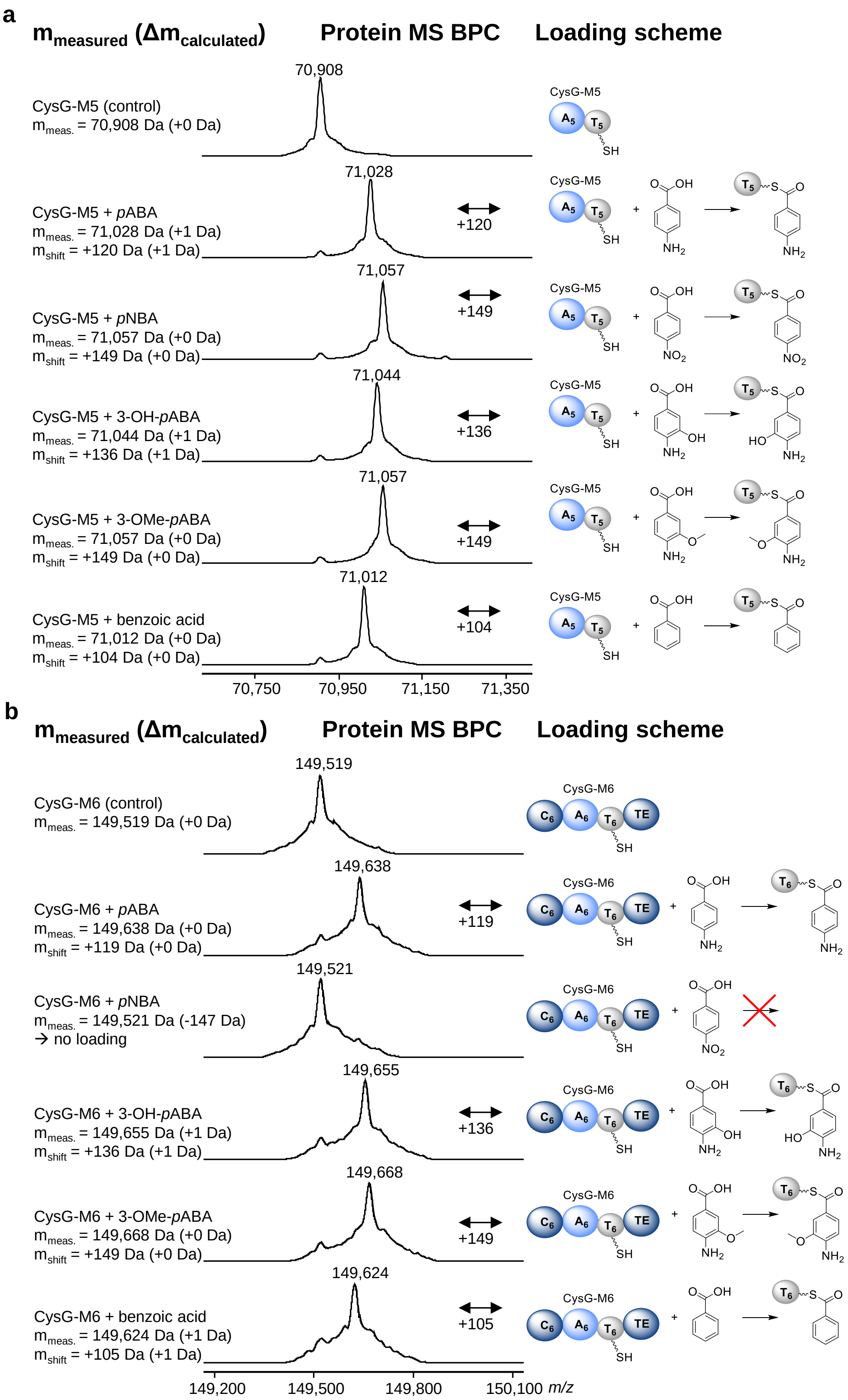

Supplementary Figure 13. Substrate specificity of CysG-M5 and CysG-M6. Observed mass shifts in deconvoluted protein MS BPCs reveal loading of *p*ABA, *p*NBA, 3-OH-*p*ABA, 3-OMe-*p*ABA and benzoic acid by CysG module 5 (**a**). The same substrate loading, except for *p*NBA, was observed for CysG module 6 (**b**). Blue sphere: adenylation domain; grey sphere: thiolation domain; red cross: no substrate loading observed.

Compound	Linker	R₁	R₂	R₃	calculated *m/z* [M+H]⁺	measured *m/z* [M+H]⁺	ret. Time [min]	Fragment a *m/z* calculated	Fragment a *m/z* measured	Fragment b *m/z* calculated	Fragment b *m/z* measured	Fragment c *m/z* calculated	Fragment c *m/z* measured
natural cystobactamid derivatives													
Cys919-1	E	*i*PrO	*i*PrO	H	920.3097	920.3090	9.06		269.0552		532.1452	725.2207	725.2190
Cys919-2	A	*i*PrO	*i*PrO	H		920.3097	10.67		269.0553		532.1451		725.2185

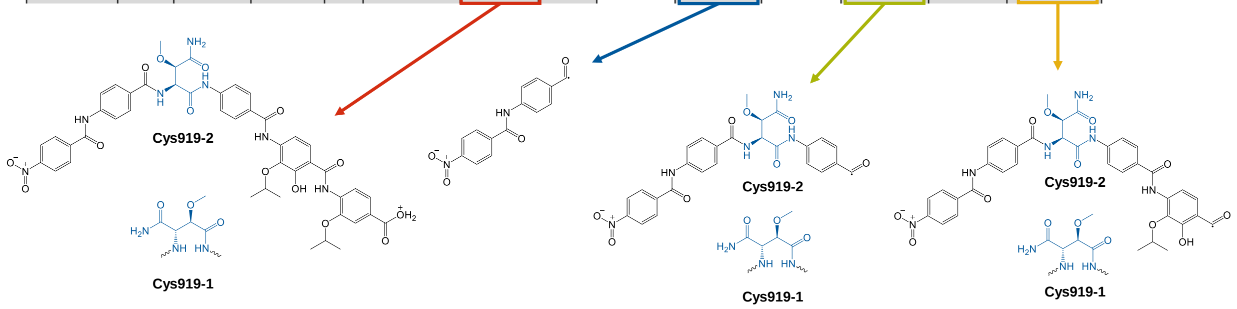

Compound	Linker	R₁	R₂	R₃	calculated *m/z* [M+H]⁺	measured *m/z* [M+H]⁺	ret. Time [min]	Fragment a *m/z* calculated	Fragment a *m/z* measured	Fragment b *m/z* calculated	Fragment b *m/z* measured	Fragment c *m/z* calculated	Fragment c *m/z* measured
unnatural cystobactamid derivatives													
Cys905-1c	G	*i*PrO	*i*PrO	H	906.2941	906.3004	8.77	269.0562	269.0578	518.1312	518.1337	711.2051	711.2092
Cys905-2c	F	*i*PrO	*i*PrO	H		906.3007	10.30		269.0576		518.1342		711.2090

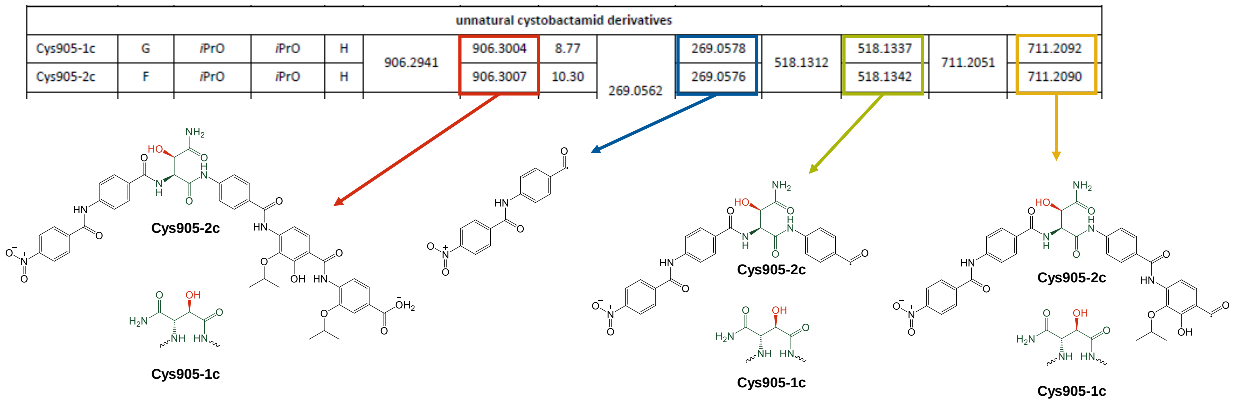

Supplementary Figure 14. Example of how structures were assigned to new cystobactamid derivatives. On top a cropped part of Supplementary Data 4 with the example Cys919-1 and Cys919-2 is shown. The measured high-resolution mass of the molecular ions is shown in the red box. The corresponding structures are shown below. The fragment ion masses are shown in the blue, green and yellow boxes with the corresponding fragment ions below. After deletion of cysQ, Cys905-1c and Cys905-2c were produced instead of Cys919-1 and Cys919-2. The measured high-resolution masses of the molecular ion and the fragment ions are shown in the red, blue, green and yellow boxes. The corresponding structures are shown below. The difference of 14 Da in the molecular ion and fragments b and c is due to a loss of a methyl group in the linker, which led to a hydroxyl group (labelled in red) instead of a methoxy group. The linker type was assigned based on the retention time difference, whereas derivatives with L-isoasparagine linkers eluted 1.4–1.6 min earlier than derivatives with L-asparagine linkers.

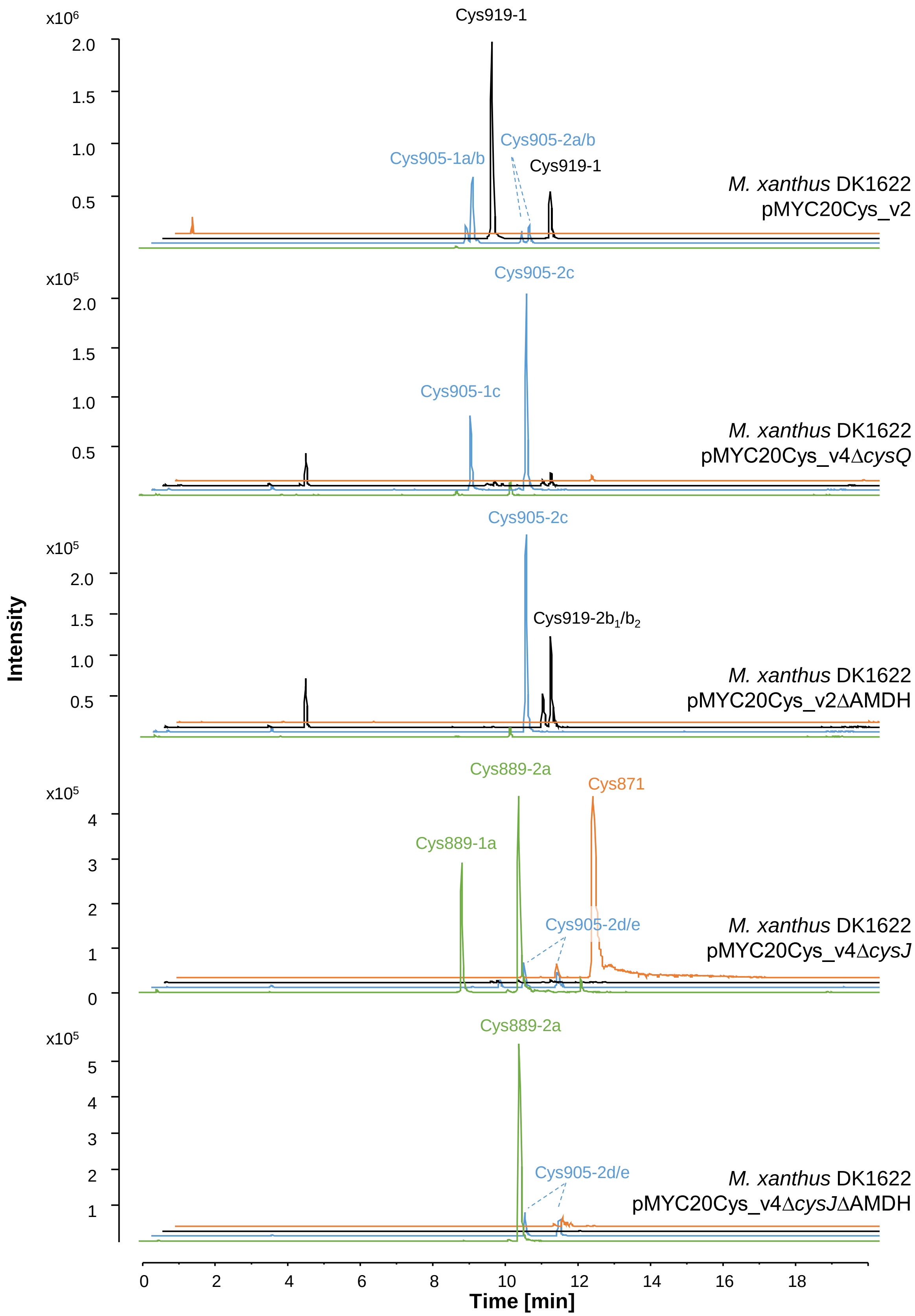

Supplementary Figure 15. Full HPLC-ESI-MS EIC traces of methanolic extracts from different heterologous strains. Stacked/overlayed view of the EICs *m/z* 920.3 $[M+H]^+$ (black), 906.3 (blue), 890.3 (green) and 872.3 (orange).

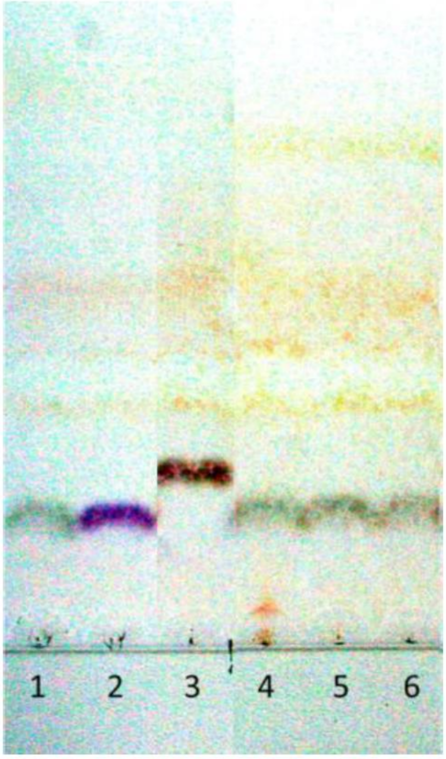

Supplementary Figure 16. TLC analysis of CysJ activity on free L-asparagine. Lane 1: L-asparagine without CysJ (control). **2:** L-aspartate without CysJ (control). **3:** L-isoasparagine without CysJ (control). **4:** L-asparagine with CysJ. **5:** L-asparagine with CysJ and α-KG. **6:** L-asparagine with denaturated CysJ and α-KG. This figure was built from different TLC plates and depicts only relevant lanes showing that CysJ has no activity on free L-Asn.

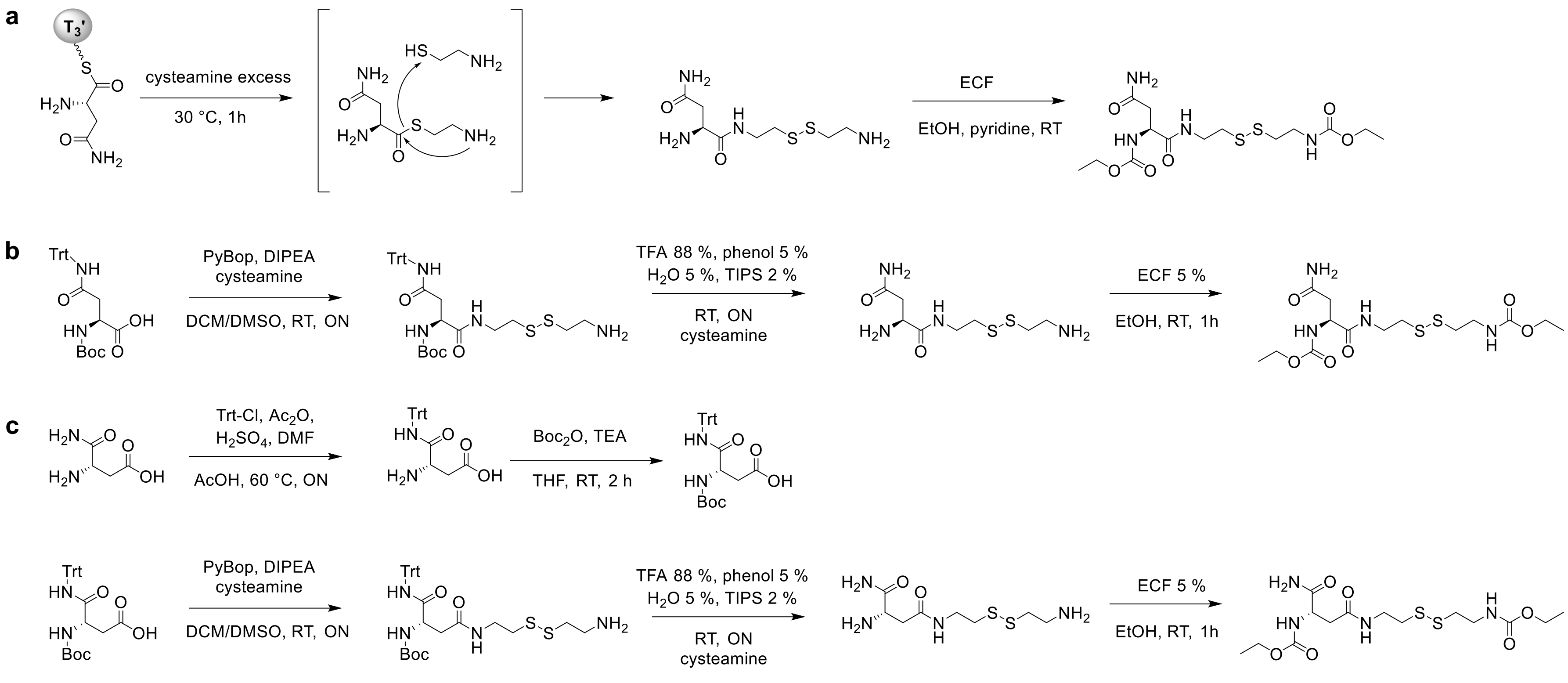

Supplementary Figure 17. Derivatization of unloaded L-asparagine and organic synthesis of di(ethylcarbonyl)asparaginyl-dicysteamine references. a: Derivatization of cysteamine unloaded L-asparagine. **b:** Organic synthesis of di(ethylcarbonyl)asparaginyl-dicysteamine. **c**: Organic synthesis of di(ethylcarbonyl) isoasparaginyl-dicysteamine.

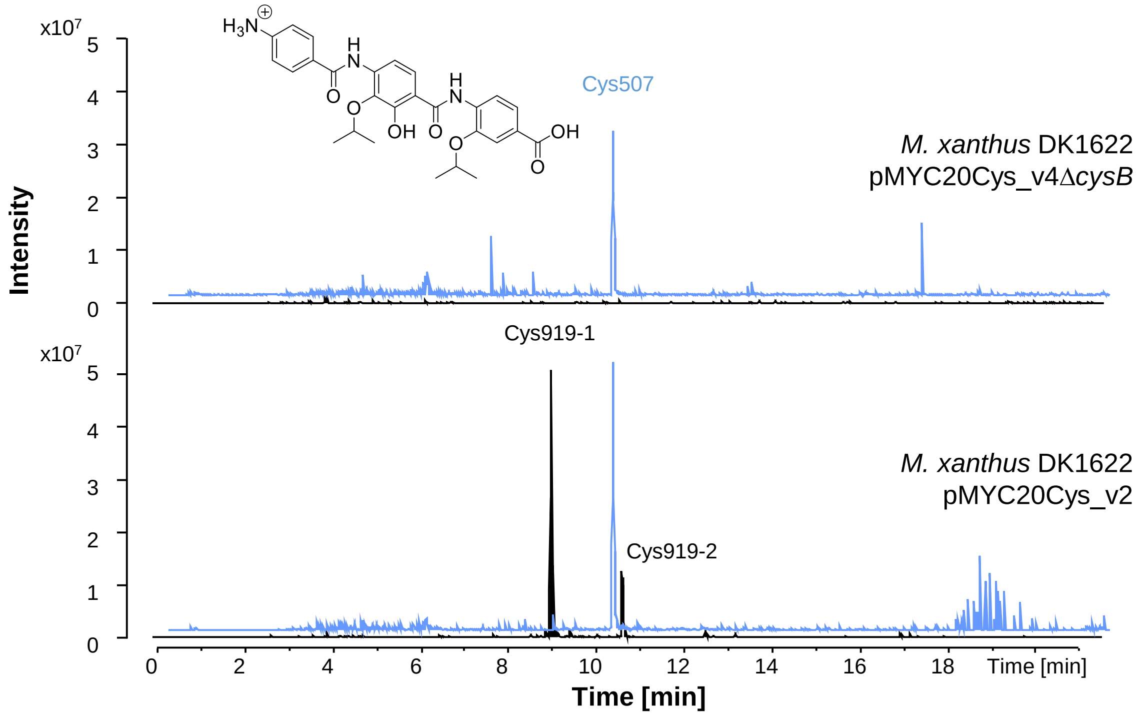

Supplementary Figure 18. HPLC-ESI-MS EIC traces of methanolic extracts from *M. xanthus* DK1622 pMYC20Cys_v4Δ*cysB*. Stacked view of the EICs *m/z* 920.3 $[M+H]^+$ (black) and 507.3 (blue). The structure of Cys507 is depicted on top.

Supplementary Table 1. Genetic elements used in the design of the modified gene cluster. Nucleotide position numbering in modified cluster refers to CysOp1 and CysOp2 separately. Numbering in brackets refers to the entire modified BGC (GenBank accession number: MT572315). Genetic elements required for TAR assembly are highlighted in ligh-grey.

Genetic element	Nucleotide position in modified cluster	Sequence origin (GenBank accession)	Nucleotide position in original sequence
**CysOp1**			
URA3 homology left	0 – 100	pRS416 (U03450)	606 - 705
Pvan (+*vanR*)	101 – 1,180	pMR3679[1]	1862 – 2941
cysA - *cysN*	1,181 – 36,816	Cbv34 gene cluster (KP836244)	9,687 - 45,325
*Xma*JI	36,817 – 36,822	-	-
tD1 terminator	36,823 – 36,871	*Myxococcus xanthus* phage Mx8[2]	-
*Xba*I, spacer, *Afl*II	36,872 – 36,889	-	-
LEU2	36,890 – 39,124	pRS415 (U03449)	3498 - 5732
URA3 homology right	39,125 – 39,224	pRS416 (U03450)	506 - 605
**CysOp2**			
URA3 homology left	0 – 100	pRS416 (U03450)	606 - 705
*Xba*I	101 – 106 (36,872 – 36,889)	-	-
Pvan	107 – 236 (36,878 – 37,007)	pMR3679[1]	2,812 – 2,941
cysO - *cysT*	237 – 9,583 (37,008 – 46,354)	Cbv34 gene cluster (KP836244)	9,347 - 0
Orf5 – *Orf1*	9,584 – 16,171 (46,355 – 52,942)	Cbv34 gene cluster (KP836244)	52,049 - 45,462
tD1 terminator	16,172 – 16,220 (52,943 – 52,991)	*Myxococcus xanthus* phage Mx8[2]	-
*Afl*II	16,221 – 16,226	-	-
LEU2	16,227 – 18,461	pRS415 (U03449)	3498 - 5732
URA3 homology right	18,462 – 18,561	pRS416 (U03450)	506 - 605

Supplementary Table 2. Removal of restriction endonuclease recognition sites (R-sites) from the modified BGC by synonymous codon substitutions. Recognition sequence of restriction endonucleases are underlined. Silent point mutations (labelled red) were made to remove R-sites inside coding sequences. Random point mutations were made to remove R-sites in intergenic regions. Nucleotide position numbering refers to original sequence. Numbering in brackets refers to revised gene cluster sequence. Sequences which do not contain blanks are non-coding sequences.

R-site removed	R-site position in original sequence	Sequence before	Sequence after
**Pvan (+*vanR*) (from pMR3679[1])**			
*Mre*I	1,900 – 1,907	CGC GCC GGC GCT	CGC GCC CGC GCT
*Bsa*I	1,998 – 2,003	CGA GAC CGC	CGA GAC GGC
*Bsa*I	2,282 – 2,287	GGT CTC	CGT CTC
**Cbv34 BGC (KP836244.2)**			
*Bsa*I	2,390 - 2,395	GGA GAC CGT	GGA GAC GGT
*Bsa*I	4,903 - 4,908	GGT CTC	CGT CTC
*Bsa*I	5,057 - 5,062	CGA GAC CGG	CGA GAC GGG
*Bsa*I	6,047 - 6,052	GGTCTC	GGTCTG
*Bsa*I	6,769 - 6,774	GGT CTC	CGT CTC
*Bsa*I	7,872 - 7,877	GGT CTC	CGT CTC
*Bsa*I	8,793 - 8,798	GGT CTC	CGT CTC
*Bsa*I	13,177 - 13,182	GAG ACC	GAG ACG
*Bsa*I	13,618 - 13,623	GAG ACC	GAG ACG
*Bsa*I	14,650 - 14,655	GAGACC	GAGACG
*Bsa*I	16,384 - 16,389	GAG ACC	GAG ACG
*Bsa*I	17,038 - 17,043	GAG ACC	GAG ACG
*Bsa*I	18,364 - 18,369	GAG ACC	GAG ACG
*Bsa*I	18,628 - 18,233	GAG ACC	GAG ACG
*Bsa*I	20,418 - 20,423	GCG GTC TCG	GCG GTC AGC
*Bsa*I	20,674 - 20,679	GAG ACC	GAG ACG
*Bsa*I	21,829 - 21,834	GAG ACC	GAG ACG
*Bsa*I	22,885 - 22,890	CTG GTC TCA	CTG GTC AGT
*Bsa*I	23,090 - 23,095	GAG ACC	GAG ACG
*Bsa*I	23,573 - 23,578	GAG ACC	GAG ACG
*Bsa*I	26,441 - 26,446	TGG GTC TCA	TGG GTC AGT
*Bsa*I	27,176 - 27,181	AAG GTC TCG	AAG GTG TCC
*Bsa*I	33,118 - 33,123 (33,115 – 33,120)	GTG GTC TCA	GTC GTG TCA
*Bsa*I	41,424 - 41,429 (41,421 – 41,426)	CTG GTC TCT	CTC GTG TCT
*Bsa*I	42,270 - 42,275 (42,267 - 42,272)	CTG AGA CCG	CTC CGA CCG
*Bsa*I	44,504 - 44,509 (44,501 - 44,506)	GAG ACC	GAG ACG
*Bsa*I	46,013 - 46,018 (44,501 - 44,506)	GGA GAC CTG	GGT GAC CTG
*Bsa*I	49,164 - 49,169 (49,164 - 49,169)	CGG GTC TCG	CGC GTC TCG
*Nde*I	9,345 – 9,350	CATATG	CATCGT
***LEU2* (U03449)**			
*Ksp*AI	5,483 – 5,488	GTTAAC	GTTAGC
*Afl*II	4,749 – 4,754	TCT TAA GTT	TCT CAA GTT

Supplementary Table 3. Name, size and restriction sites or splitter elements (SEs) of the thirteen gene synthesis cluster fragments. Spacer sequences (sp) were introduced between SE restriction sites.

**Cluster fragment**	**Description**	**Size [bp]**	**Flanking restriction sites or SEs**
hPvanABC	*URA3* homology, *vanR*, Pvan, *cysA*, *cysB*, *cysC*	3,878	SE1 (5'): *Kpn*I-sp(CGTTAA)-*Nhe*I-*Bsa*I<br>SE2 (3'): *Bsa*I-*Afl*II-sp(GATTGC)-*Pme*I
DEF5-G	*cysD*, *cysE*, *cysF*, (5') 150 bp of *cysG*	4,413	SE2 (5'): *Afl*II-*Bsa*I<br>SE3 (3'): *Bsa*I-*Avr*II-sp(AGCCTA)-*Pme*I-3'
G	*cysG*	5,872	5'-*Bsa*I<br>3'-*Bsa*I
3-GHIJ5-K	(3') 173 bp of *cysG*, *cysH*, *cysI*, *cysJ*, (5') 250 bp of *cysK*	5,330	5'-*Bsa*I<br>3'-*Bsa*I
3-GHIJ5-K_flong_v2	(3') 1,222 bp of 3-GHIJ5-K fragment, 1,796 bp of pGH cloning vector backbone	3,018	-
K1_v2	*cysK*	2,542	SE4 (5'): *Kpn*I-sp(GTTACG)-*Pac*I-*Bsa*I<br>SE5 (3'):*Bsa*I-*Hind*III-sp(GACCTA)-*Pme*I
K2_v2	*cysK*	5,499	SE5 (5'): *Kpn*I-sp(CCAGCT)-*Hind*III-*Bsa*I<br>SE6 (3'): *Bsa*I-*Ase*I-sp(AGCCAT)-*Pme*I
K3_v2	*cysK*	5,563	SE6 (5'): *Kpn*I-sp(TATCCG)-*Ase*I-*Bsa*I<br>SE7 (3'): *Bsa*I-*Eco*RI-sp(AGCCAT)-*Pme*I
3-KL	(3') 150 bp of *cysK*, *cysL*	3,322	SE8 (5'): *Kpn*I-sp(AGGCGT)-*Mre*I-*Bsa*I<br>SE9 (3'): *Bsa*I-*Spe*I-sp(GACTCC)-*Pme*I
NtD1LEU2h	*cysN*, tD1, *LEU2*, *URA3* homology	3,602	SE9 (5'): *Spe*I-*Bsa*I<br>SE10 (3'): *Bsa*I-*Not*I-sp(GCAGTC)-*Pme*I
hPvanOPQRS	*URA3* homology, Pvan, *cysO*, *cysP*, *cysQ*, *cysR*, *cysS*	5,687	5'-*Bsa*I<br>3'-*Bsa*I
T-ABC15-2	*cysT*, *ABC1* (*Orf5*), (5') 827 bp of *ABC2* (*Orf4*)	5,666	5'-*Bsa*I<br>3'-*Bsa*I
ABC3-2345tD1	(3') 1379 bp of *ABC2* (*Orf4*), *ABC3* (*Orf3*), *ABC4* (*Orf2*), *ABC5* (*Orf1*), tD1	5,139	SE11 (5'): *Swa*I-*Bsa*I<br>SE12 (3'): *Afl*II-^GTTT^C-*Bsa*I-*Pme*I
LEU2pc	*URA3* homology, *LEU2*, *URA3* homology	2,457	5'- H_L-*Afl*II<br>3'-H_R -^GTTT^C-*Bsa*I-3'

Supplementary Table 4. Genetic elements used in the design of the pMYC vector system.

Genetic element	Nucleotide position in pMYC building block	Sequence origin (GenBank accession)	Nucleotide position in original sequence
**basic pMYC (MT572316)**			
*Ksp*AI, spacer, *Nhe*I	0 – 18	-	-
URA3	19 – 1,130	pRS416(U03450)[1]	187 – 1,298
*Apa*LI, spacer, *Xho*I	1,131 – 1.148	-	-
p15A ori	1,149 – 1,981	pACYC177 (X06402)[10]	766 - 1,598
cmR (*cat*)	1,982 – 3,041	pACYC184 (X06403)[11]	3,605 - 419
IR2	3,042 – 3,090	pFNLTP16 H3 (DQ236098)	4,277 - 4,325
oriT/*traJ*	3,091 – 3,781	*Pseudomonas aeruginosa* plasmid Birmingham IncP-alpha (L27758)	50,687 - 51,377
*Not*I, spacer, *Sna*BI	3,782 – 3,801	-	-
CEN6/*ARS4*	3,802 - 4,570	pRS415 (U03449)	2,729 - 3,497
*Mss*I, spacer, *Pac*I, *Sma*I, spacer, *Mre*I	4,571 – 4,612	-	-
tD2 terminator	4,613 - 4,662	*Myxococcus xanthus* bacteriophage Mx8[2]	-
**Mx8-tetR**			
*Mss*I, spacer, *Pac*I	0 – 22	-	-
mx8 integrase	23 - 1,853	Bacteriophage Mx8 imm (BMU64984)	4,979 - 6,809
*Bst*1107I, spacer, *Spe*I	1,854 - 1,871	-	-
tetR	1,872 – 3,250	pALTER1(R) (X65334)	451 - 1,829
*Sma*I, spacer, *Mre*I	3,251 – 3,270	-	-
**Mx9-kanR**			
*Mss*I, spacer, *Pac*I	0 – 22	-	-
mx9 integrase	23 - 1,851	*mx9* sequence (AY247757)	430 – 2,255
*Bst*1107I, spacer, *Spe*I	1,852 – 1,869	-	-
kanR (*aph(3')-Ia*)	1,870 – 2,849	pACYC177 (X06402)[10]	1,816 - 2,797
*Sma*I, spacer, *Mre*I	2,850 – 2,869	-	-

Supplementary Table 5. Removal of restriction sites (R-sites) from pMYC building blocks by synonymous codon substitutions. Recognition sequence of restriction endonucleases are underlined. Silent point mutations (labelled red) were made to remove R-sites inside coding sequences. Random point mutations were made to remove R-sites in intergenic regions. Nucleotide position numbering refers to original sequence. Sequences which do not contain blanks are non-coding sequences.

R-site removed	R-site position in original sequence	Sequence before	Sequence after
	**p15A (X06402)**		
*Bst*1107I	1,577 - 1582	GTATAC	GCATAC
*Nhe*I	1,588 – 1,593	GCTAGC	GCTACC
	***oriT/traJ* (L27758)**		
*Mre*I	51,117 – 51,124	CGCCGGCG	CCCCGGCG
	**CEN6/ARS4 (U03449)**		
*Apa*LI	3,488 – 3,493	GTGCAC	GTGGAC
	**URA3 (U03450)**		
*Nde*I	329 - 334	CATATG	CATATC
*Bst*1107I	741 - 746	GTA TAC	GTC TAC
*Bsa*I	1,085 – 1,090	GTG GTC TCT	GTT GTC TCT
*Bst*1107I	1,246 – 1,251	GTA TAC	GTC TAC
	**tnp (DQ236098)**		
*Ksp*AI	981 – 986	AGT TAA CAG	AGT TAA CAG
*Eco*RV	1,119 – 1,124	TGA TAT CTT	ACT TAT CTT
*Nde*I	1,409 – 1,414	CATATG	CATATC
	***ampR* (*bla*) (X06402)**		
*Apa*LI	8,342 – 8,347	GGT GCA CGA	GGT GCC CGA
*Bsa*I	8,942 – 8,947	GGG TCT CGC	GGG TCA CGC
	***mx8* (BMU64984)**		
*Bsa*I	5,937 – 5,942	GAG ACC	GAG ACG
*Not*I	6,711 – 6,718	GCG GCC GCC	GCC GCG GCC
	***tetR* (X65334)**		
*Eco*RV	640 – 645	GAT ATC	GAC ATT
*Nhe*I	684 – 689	CTG CTA GCG	CTG CTT GCG
	***mx9* (AY247757)**		
*Nde*I	985 - 990	CAT ATG	CAC ATG
*Bst*1107I	2,057 – 2,062	GGT ATA CCG	GGG ATA CCG
*Mre*I	2,139 – 2,146	GCC GCC GGC GTC	GCC GCC GGT GTC
	***kanR* (*aph(3')-Ia*) (X06402)**		
*Xho*I	1,952 – 1,957	TGC TCG AGG	TGC TCA AGG
*Sma*I	2,226 – 2,231	CCC GGG	CCA GGG

Supplementary Table 6. Source of vector and insert DNA and restriction endonucleases used for construction of plasmids in this work. [a] SEs were removed afterwards during desplitting with *Bsa*I, [b] blunt end ligation since pSynbio1 was linearized using only *Pme*I.

**Product generated**	**Vector**	**Insert**	**Insert source**	**Restriction enzymes**
pSynbio1hPvanABC	pSynbio1	hPvanABC	Gene synthesis fragment	*Kpn*I/*Pme*I
pSynbio1hPvanABCDEF5-G (+SE)	pSynbio1hPvanABC	DEF5-G	Gene synthesis fragment	*AflII*/*Pme*I
pSynbio1hPvanABCDEF5-G	pSynbio1hPvanABCDEF5-G (+SE)	-	-	*Bsa*I
pGH-GHIJ5-K_v2	pGH-3-GHIJ5-K	3-GHIJ5-K_flong_v2	Gene synthesis fragment	*Xho*I/*Sca*I
pSynbio1K1_v2	pSynbio1	K1	Gene synthesis fragment	*Kpn*I/*Pme*I
pSynbio1K12_v2	pSynbio1K1	K2	Gene synthesis fragment	*Hind*III/*Pme*I
pSynbio1K123_v2 (+SE)[a]	pSynbio1K12	K3	Gene synthesis fragment	*Ase*I/*Pme*I
pSynbio1K123_v2	pSynbio1K123 (+SE)	-	-	*Bsa*I
pSynbio13-KL	pSynbio1	3-KL	Gene synthesis fragment	*Kpn*I/*Pme*I
pSynbio13-KLNtD1LEU2h (+SE)[a]	pSynbio13-KL	NtD1LEU2h	Gene synthesis fragment	*Spe*I/*Pme*I
pSynbio13-KLNtD1LEU2h	pSynbio13-KLNtD1LEU2h (+SE)	-	-	*Bsa*I
pSynbio1ABC3-2345tD1	pSynbio1	ABC3-2345tD1	Gene synthesis fragment	*SwaI/Pme*I[b]
pSynbio1ABC3-2345tD1LEU2h	pSynbio1ABC3-2345tD1	LEU2pc	Gene synthesis fragment	*Afl*II/*Bsa*I
pMYC20	pMYC	TetR-mx8	Gene synthesis fragment	*Sma*I/*Pac*I
pMYC21	pMYC	KanR-mx9	Gene synthesis fragment	*Sma*I/*Pac*I
pMYC20CysOp1_v2	pMYC20preCysOp1	K123_v2	pSynbio1K123_v2	*Bsa*I
pMYC20Cys_v2	pMYC20CysOp1_v2	CysOp2	pMYC21CysOp2	*Xba*I/*Afl*II

Supplementary Table 7. Manipulated plasmids generated via Red/ET recombineering and restriction hydrolysis/re-ligation.

**Product generated**	**Description**	**Vector used for Red/ET**	**Red/ET product**
pMYC20Cys_v2ΔAMΔH	Deletion of X domain in *cysH*	pMYC20Cys_v2	pMYC20Cys_v2-XampR
pMYC20Cys_v4ΔcysQ	Deletion of *cysQ*	pMYC20Cys_v2	pMYC20Cys_v2-QampR
pMYC20Cys_v4ΔcysJ	Deletion of *cysJ*	pMYC20Cys_v2	pMYC20Cys_v2-JampR
pMYC20Cys_v4ΔcysJΔAMDH	Deletion of *cysJ* and X domain in *cysH*	pMYC20Cys_v4-J	pMYC20Cys_v4-J-XampR
pMYC20Cys_v4ΔcysB	Deletion of *cysB*	pMYC20Cys_v2	pMYC20Cys_v2-BampR
pMYC20Cys_v4ΔcysR	Deletion of *cysR*	pMYC20Cys_v2	pMYC20Cys_v2-RampR
pMYC20Cys_v4ΔcysC	Deletion of *cysC*	pMYC20Cys_v2	pMYC20Cys_v2-CampR

Supplementary References

1. Iniesta, A. A., García-Heras, F., Abellón-Ruiz, J., Gallego-García, A. & Elías-Arnanz, M. Two systems for conditional gene expression in *Myxococcus xanthus* inducible by isopropyl-ß-D-thiogalactopyranoside or vanillate. *J. Bacteriol.* **194,** 5875–5885 (2012).

2. Magrini, V., Creighton, C. & Youderian, P. Site-specific recombination of temperate *Myxococcus xanthus* phage Mx8: genetic elements required for integration. *J. Bacteriol.* **181,** 4050–4061 (1999).

3. Yan, F. *et al.* Synthetic biology approaches and combinatorial biosynthesis towards heterologous lipopeptide production. *Chem. Sci.* **9,** 7510–7519 (2018).

4. Agatep, R., Kirkpatrick, R. D., Parchaliuk, D. L., Woods, R. A. & Gietz, R. D. Transformation of Saccharomyces cerevisiae by the lithium acetate/single-stranded carrier DNA/polyethylene glycol protocol. *Technical Tips Online* **3,** 133–137 (1998).

5. Baumann, S. *et al.* Cystobactamids: myxobacterial topoisomerase inhibitors exhibiting potent antibacterial activity. *Angew. Chem. Int. Ed.* **53,** 14605–14609 (2014).

6. Edgar, R. C. MUSCLE: multiple sequence alignment with high accuracy and high throughput. *Nucleic Acids Res.* **32,** 1792–1797 (2004).

7. Cociancich, S. *et al.* The gyrase inhibitor albicidin consists of *p*-aminobenzoic acids and cyanoalanine. *Nat. Chem. Biol.* **11,** 195–197 (2015).

8. Crooks, G. E., Hon, G., Chandonia, J. M. & Brenner, S. E. WebLogo: a sequence logo generator. *Genome Res.* **14,** 1188–1190 (2004).

9. Kim, B.-M., van Minh, N., Choi, H.-Y. & Kim, W.-G. Coralmycin derivatives with potent anti-gram negative activity produced by the Myxobacteria *Corallococcus coralloides* M23. *Molecules* **24,** 10.3390/molecules24071390 (2019).

10. Rose, R. E. The nucleotide sequence of pACYC177. *Nucleic Acids Res* **16,** 356 (1988).

11. Rose, R. E. The nucleotide sequence of pACYC184. *Nucleic Acids Res* **16,** 355 (1988).
