# Supplementary material for: In vivo and in vitro reconstitution of unique key steps in cystobactamid antibiotic biosynthesis
Source: Nat Commun. 2021 Mar 16;12:1696. doi: 10.1038/s41467-021-21848-3 (PMC7966384; doi:10.1038/s41467-021-21848-3)
Supplement: Supplementary file 3 — Description of Additional Supplementary Files [file 41467_2021_21848_MOESM3_ESM.pdf]

Description of additional supplementary files

Title: Supplementary Data 1

Description: Strains used and generated in this work.

Title: Supplementary Data 2

Description: Plasmids used and generated in this work.

Title: Supplementary Data 3

Description: Oligonucleotides used in this work.

Title: Supplementary Data 4

Description: UPLC-HRMS and MS2 data of natural and unnatural cystobactamids produced in the heterologous *M. xanthus* DK1622 strains. Linker and R1, R2, R3 classification shown in Figure 1. Fragmentation pattern shown in Supplementary Figure 6. BPC and EIC of minor and major natural cystobactamid derivatives shown in Supplementary Figure 8. Previously described derivatives are marked in grey.
