# Supplementary material for: In vivo and in vitro reconstitution of unique key steps in cystobactamid antibiotic biosynthesis
Source: Nat Commun. 2021 Mar 16;12:1696. doi: 10.1038/s41467-021-21848-3 (PMC7966384; doi:10.1038/s41467-021-21848-3)
Supplement: Supplementary file 5 — Reporting Summary [file 41467_2021_21848_MOESM5_ESM.pdf]

nature research

Corresponding author(s): Rolf Müller

Last updated by author(s): Jan 29, 2021

Reporting Summary

Nature Research wishes to improve the reproducibility of the work that we publish. This form provides structure for consistency and transparency in reporting. For further information on Nature Research policies, see our Editorial Policies and the Editorial Policy Checklist.

Statistics

For all statistical analyses, confirm that the following items are present in the figure legend, table legend, main text, or Methods section.

n/a	Confirmed	
☐	☒	The exact sample size (n) for each experimental group/condition, given as a discrete number and unit of measurement
☐	☒	A statement on whether measurements were taken from distinct samples or whether the same sample was measured repeatedly
☒	☐	The statistical test(s) used AND whether they are one- or two-sided<br>*Only common tests should be described solely by name; describe more complex techniques in the Methods section.*
☒	☐	A description of all covariates tested
☒	☐	A description of any assumptions or corrections, such as tests of normality and adjustment for multiple comparisons
☒	☐	A full description of the statistical parameters including central tendency (e.g. means) or other basic estimates (e.g. regression coefficient) AND variation (e.g. standard deviation) or associated estimates of uncertainty (e.g. confidence intervals)
☒	☐	For null hypothesis testing, the test statistic (e.g. F, t, r) with confidence intervals, effect sizes, degrees of freedom and P value noted<br>*Give P values as exact values whenever suitable.*
☒	☐	For Bayesian analysis, information on the choice of priors and Markov chain Monte Carlo settings
☒	☐	For hierarchical and complex designs, identification of the appropriate level for tests and full reporting of outcomes
☒	☐	Estimates of effect sizes (e.g. Cohen's d, Pearson's r), indicating how they were calculated

Our web collection on statistics for biologists contains articles on many of the points above.

Software and code

Policy information about availability of computer code

Data collection	Bruker Daltonics HyStar 3.2 (Build 49.9) and Bruker Compass otofControl version 3.4 for scan control and data acquisition (ToF-MS) or Bruker trapControl version 8.0 (3D ion trap)<br>Chromeleon version 6.80 for LC control
Data analysis	Bruker DataAnalysis version 4.4 for MS analysis<br>MS Powerpoint 2016 for image processing<br>ChemDraw Professional version 17.1.0.105 (19) for image processing and creation of chemical structures images.<br>MS Excel 2016 for calculation of production titers<br>Geneious version 10.1.3 (Biomatters Ltd.) for DNA sequence analysis<br>EMBOSS 6.5.7 dottup to create dotplots

For manuscripts utilizing custom algorithms or software that are central to the research but not yet described in published literature, software must be made available to editors and reviewers. We strongly encourage code deposition in a community repository (e.g. GitHub). See the Nature Research guidelines for submitting code & software for further information.

Data

Policy information about availability of data

All manuscripts must include a data availability statement. This statement should provide the following information, where applicable:

- Accession codes, unique identifiers, or web links for publicly available datasets
- A list of figures that have associated raw data
- A description of any restrictions on data availability

The sequences described within the manuscript were deposited in the GenBank database under the following accession numbers:
- revised native cystobactamid BGC (KP836244.2)
- modified cystobactamid BGC (MT572315)
- basic pMYC (MT572316)
- pMYC20 (MT572317)
- pMYC21 (MT572318)

All data generated or analyzed during this work are available in this article and the Supplementary Information files as well as from the corresponding author on reasonable request.

Field-specific reporting

Please select the one below that is the best fit for your research. If you are not sure, read the appropriate sections before making your selection.

- [x] Life sciences
- [ ] Behavioural & social sciences
- [ ] Ecological, evolutionary & environmental sciences

For a reference copy of the document with all sections, see nature.com/documents/nr-reporting-summary-flat.pdf

Life sciences study design

All studies must disclose on these points even when the disclosure is negative.

Sample size	Our study design did not include taking a distinct number of samples from a larger pool of samples. For the intact protein LC-MS experiments, each protein was purified once and used for the biochemical assays. For heterologous production experiments 1-3 verified clones per transformed construct were chosen for cultivation.
Data exclusions	No data were excluded.
Replication	To check robustness, we repeated experiments at least three times and ensured reported results were reproducible. All attempts of replication were successful.
Randomization	Sample measurements were not randomized, because we did not compare groups which start at the same conditions and are then exposed to different treatments. Here we compared samples with different starting conditions, so the experiment outcome is not biased by the experimenter.
Blinding	Blinding was not a requirement for our study, because this study did not include any kind of participants which could be influenced by a non-blinded study design. The data types collected herein (e.g. LC-MS chromatograms) are not significantly impacted by investigator prior knowledge.

Reporting for specific materials, systems and methods

We require information from authors about some types of materials, experimental systems and methods used in many studies. Here, indicate whether each material, system or method listed is relevant to your study. If you are not sure if a list item applies to your research, read the appropriate section before selecting a response.

Materials & experimental systems

n/a	Involved in the study
[x]	[ ] Antibodies
[x]	[ ] Eukaryotic cell lines
[x]	[ ] Palaeontology and archaeology
[x]	[ ] Animals and other organisms
[x]	[ ] Human research participants
[x]	[ ] Clinical data
[x]	[ ] Dual use research of concern

Methods

n/a	Involved in the study
[x]	[ ] ChIP-seq
[x]	[ ] Flow cytometry
[x]	[ ] MRI-based neuroimaging
