# Supplementary material for: In vivo and in vitro reconstitution of unique key steps in cystobactamid antibiotic biosynthesis
Source: Nat Commun. 2021 Mar 16;12:1696. doi: 10.1038/s41467-021-21848-3 (PMC7966384; doi:10.1038/s41467-021-21848-3)
Supplement: Supplementary file 2 — Peer Review File [file 41467_2021_21848_MOESM2_ESM.pdf]

Reviewer #1 (Remarks to the Author):

In this manuscript, Grob et al. describe characterization of several important enzymes involved in synthesis of a family of cystobactamid (Cys) compounds. The authors designed and assembled a synthetic construct of a Cys biosynthetic gene cluster for heterologous expression in M. xanthus. The authors also created a number of deletion mutations to this construct. Additionally, the authors built a number of single gene expression constructs for heterologous expression in E. coli, purification, and in vitro characterization. Combinatorically and systematically testing the function of these constructs, the authors contributed tremendously to improved understanding of the mechanisms Cys synthesis. Of particular interest, the authors identified an AMDH domain that can catalyze an aminomutase- or an amide dehydratase-type reaction depending on the presence of active CysJ.

The work by Grob et al. is of high quality; their discoveries are very interesting and significantly advance the natural product research field. I heartily recommend the manuscript be accepted for publication in Nature Communications. However, a few things could be improved, as listed below.

Major Improvements Needed
1. In the MS analyses, I see –5 to +3 Da variations among proteins between calculated and measured masses in Figures 2, 3, and 6. I am not familiar with protein MS analysis, but the variations seem large compared with most other MS-based methods. It would be helpful for the authors to provide possible explanations for the variations.
2. To validate the results, the authors need to provide supplementary information on how they identified the structures of the 18 new Cys derivatives that were found through the studies.
3. For Figures 4 and 5, the authors should provide full LC-MS EIC traces in order to fully validate the results (i.e., 427.1 for CysH + L-Asn [or isoAsn] and 411.1 for CysH + CysJ + L-Asn [or isoAsn] for Figure 4, and EIC 920.3, 906.3, 890.3, and 872.3 traces for all experiments).
4. The authors can elaborate the explanation how the product selectivity toward Cys905-2c over Cys889-1c and Cys871 is decided. Is it kinetically driven? Is the presence of CysJ important even if it is inactive?
5. I understand that that the authors' attempt to crystallize the AMDH domain failed. Alternatively, the authors are recommended to mutate some of the conserved motifs listed in Supplementary Figure 10 and see how AMDH activity changes.

Minor Improvements Needed
1. Pg 3, Figure 1. Because R1 and R2 only refer to MeO and OH, respectively, the authors could simply incorporate them as part of the Albicidin structure.
2. Pg 3, Line 43. It would be helpful if the authors refer to Table 1.
3. Pg 9, Table. Is this part of Table 1 or Table 2? The titles of the first two columns are different from those of Table 1.
4. Pg 9, Table. Should Cyc899-1a and Cys899-2a be Cys899-1c and Cys899-2c?
5. Pg 13, Line 199. Please specify which supplementary information is referred to (I assumed it is Supplementary Table 14).
6. Pg 13, Line 210. Please specify which supplementary information is referred to (I assumed it is Supplementary Table 15).
7. The labels in Supplementary Figure 3 is too small.

Reviewer #2 (Remarks to the Author):

The paper by Groß et al desribes details in the biosynthesis of the potent antibiotic cystobactamid and includes compound identification of several novel derivatives, in vitro reconstitution of important steps and branching points in the biosynthesis and describes the biochemical function of a new domain in

NRPS that has aminomutase and amide dehydratase activity.
This is a complex but excellent paper with lots of new details that shows how far one can go even with complex natural products with respect to analysis and biochemistry and it must be published.
However, I have a few suggestions that might help to even improve it:
- For NRPS introduction, please also cite Mainz and Süßmuth, the latest and most comprehensive review in the field
- For all MS analysis data: Did the authors also perform Papst ejection assays?
- Fig 3g: delta in protein name
- Fig. 3: How ist the isomerization in f (last step) confirmed?
- Line 200: be more specific where to find the data in the supplementary information
- Fig. 6: In order to show shuttling the arrows should be directional not bidirectional as some of them are?
- Line 342: the sentence with the reversible reaction is unclear to me. Isn't in Fig. 6b twice the same result just with a different pH? Please explain in more detail.
- The data for the shortened derivative Cys507 is missing and should be added to the SI
- Line 437 and elsewhere: Since the authors have some of the main compounds isolated (also from previous experiments), it should be possible to give the amounts based as MS quantification with the major compound as standard.
- Structures in SI Fig 7 are too small
- SI Fig 9: no bend triple bond in bottom molecule

Reviewer #3 (Remarks to the Author):

Please see attached a separate PDF file for comments.

Reviewer #4 (Remarks to the Author):

The paper entitled « In vivo and in vitro reconstitution of unique key steps in cystobactamid antibiotic biosynthesis" by S. Gross et al depicts a quite impressive piece of work aiming, at the first place, at correcting the previously published biosynthetic genes cluster (BGC) involved in the biosynthesis of cystobactamids. Also described is the transfer of the whole BGC into a heterologous host using a state-of-the-art process. But the largest part of the paper allows the authors to propose updated detailed processes for the biosynthesis of the linker part of the molecules (with a special focus on the AMDH domain), as well as to describe the action of several genes involved in hydroxylations and O-methylations of the linker and of the pABA units. Finally, this long paper investigates the shuttling mechanism allowing the transfer of the linker part from a stand-alone NRPS module to the main assembly chain.

Overall, the paper is very well written and one can read it in an easy and pleasant manner. Apart from a few exceptions listed below, extensive information is given, either in the main paper or in the supplementary information, regarding experimental conditions and results. Nevertheless, I have some major and minor remarks (see below) that I think, should the authors take them into account, would improve the actual manuscript.

Major Remark 1:
It is obvious that a manuscript centered on cystobactamids requires full comparison with their close neighbors from the albicidin family, considering the many similarities in the structures and in the biosynthetic schemes of the molecules from both families. Thus, several times throughout the paper, the authors compare their results with the corresponding findings in the albicidins family. However, the statements which concern albicidins are often incomplete, to such an extent that they appear almost wrong. I am wondering whether the authors have read the recent papers on albicidins (a small

dozen since 2014). I would urge them to carefully read them, and to correct some of their statements regarding albicidins (see two examples below).

Example 1: page 3, legend Figure 1, last sentence (see also page 4, lines 63-64): these statements correspond to the beta-albicidin. However, at least nine other natural analogues have been identified and fully structurally described (see von Eckardstein et al, 2017, Chemistry, a European Journal 23, 15316-15321), some of which do not display beta-cyanoalanine as linker but rather beta-O-methyl-asparagine (two derivatives) or beta-O-methyl-cyanoalanine (two derivatives).

Example 2: page 24, lines 403-408: The authors speculate that, in the albicidin biosynthesis, the domain similar to AMDH "is exclusively dehydrating L-asparagine because no essential interaction partner (like CysJ in cystobactamid biosynthesis) catalyzes hydroxylation of the substrate prior to a potential isomerization". This is rather presumptuous since the cited paper from the Cociancich group describes only the first-ever isolated member of the albicidin family (namely the beta-albicidin). It appears that this molecule displays a beta-cyanoalanine as linker, and as such, the authors proposed the action of the AMDH-like domain to transform asparagine in beta-cyanoalanine. At no point, the authors wrote that the AMDH domain might not have any other function. Moreover, there is also, in the albicidin BGC, a homolog of CysJ (Alb08) which might as well play the role of the interaction partner. Also, I do not understand the last sentence of the same paragraph (page 24, lines 411-413): I do not see the point of expressing heterologously CysJ and CysQ in the native albicidin producer or the heterologous producer since both of them do possess the corresponding homologs (i.e. Alb08 and Alb06). As a conclusion for this example, this paragraph contains many incomplete or false statements, and this leads me to recommend that the authors either correct their statements or delete the whole paragraph.

The requested corrections in this major remark 1 will also require some additional references (at least von Eckardstein et al., 2017 which describe many of the natural analogues from the albicidin family). I am also wondering why the authors do not mention the coralmycins, which are also strongly resembling cystobactamids and albicidins, although there is less literature available about this family.

Major remark 2:
Page 10/11, Figure 2 and corresponding legend (Substrate specificity of CysH): I would have been interested in knowing the respective ratios of incorporation of all the tested substrates. The authors indicate a yes-or-no incorporation for a given substrate, but to which extent is a yes meaning? Is there any preferred substrate for CysH? The same question applies for other experiments (page 21, lines 352-354 and Suppl. Fig 11, as well as lines 366-369 and Suppl. Fig. 13).
Also, the authors state several times that cystobactamids exhibiting L-asparagine as linker display a more potent antibacterial activity. I would like to know which is/are the targeted bacteria. Also, I think it would be of great importance to know the MIC or IC50 (or any other unit for antibacterial activity) for all the mutants of the cystobactamid BGC in the heterologous host (compared to the WT) (for instance, the CysQ, CysJ and AMDH mutants, respectively). This would complement the information gathered by mass spectrometry (see figure 5, for instance).
On this aspect, the authors ask (page 26, lines 450 and following) why Nature established a complex biosynthesis route (trans-acting AMDH and shuttling via CysB) to produce cystobactamids which are less biologically active. This question arises because they observe that, when targeting specific bacteria (and again, I would like to know which ones), cystobactamids with an asparagine as linker is more active than a cystobactamid with a beta-cyanoalanine for instance. I think that really depends on the target. Especially, the authors should remember that cystobactamids are naturally produced by a soil bacterium. Who knows which other bacteria (probably much different from the standard lab strains) they have to leave with, and eventually to fight using cystobactamids. I am convinced that Nature would not, by any means, keep such an energy-demanding complex process for nothing, and therefore I am sure that the answer lies in the natural environment of the cystobactamids-producing myxobacteria.

Minor remarks:

1. page 5, lines 74/75: some domains do indeed catalyze several reactions (dual condensation / epimerization domains, for instance).

2. page 11, lines 179-181: This sentence is not clear. It must be either better explained or removed.

3. page 11, lines 181-186 and fig. 3c: The result depicted here is remarkably interesting (substrate dehydration observed after a prolonged incubation). But there are some discrepancies between what is said here about the incubation time ("longer than 5 min") and what is mentioned elsewhere in the paper (page 38, lines 738-739, Materials and methods indicates 2h). Please make it clear.

4. page 14, line 220: Add a space after "dicysteamine".

5. page 14, line 227: Add a point after "M"

6. page 17, line 265: "leads to a much less abundant product": How much less abundant?

7. page 23, line 378, last sentence of the figure legend: "Release of the final product by the TE domain". This is not depicted on fig 7, page 22.

8. page 34, line 650: Replace "Supplementary Table 10 » by "Supplementary Table 11"

9. page 34, line 653: Write "M. xanthus" in italics

10. page 3, line 685: Replace "and 0.01 mg mL-1" by "and 0.1 mg mL-1"

11. Suppl. Info. page 8, paragraph "Synthesis of ...", line 5: "and 50 µl DMF are added": this step does not appear in Suppl. Fig 15c.

12. Suppl. Info. page 17, Suppl. Fig. 7: Drawings of the molecules are too small. One does not see the structural differences

13. Suppl. Info. page 18, legend Suppl. Fig. 8: Many errors lie in legend of Suppl. Fig. 8b:
* Remove "BPC and"
* "EIC 890.29 (green)" does not appear in the figure
* Add "(purple)" after "EIC 936.30"
* "show the existence of Cys919 1/2, Cys507, ..." does not correspond to part b of the figure. It is a copy and paste from part a. Please correct.

14. Suppl. Info. page 19, legend Suppl. Fig. 9: Replace "see Suppl. Fig. 12" by "see Suppl. Fig. 10"

15. Suppl. Info. page 40, Suppl. Table 10:
* line 2: Replace "see Suppl. Fig. 1" by "see Suppl. Fig. 6"
* Line 3: "see Suppl. Fig. 6" by "see Suppl. Fig. 8"

I recommend this paper to be accepted after revision according to the above review

Authors response labelled in red. The page and line indications refer to the initially submitted manuscript.

Reviewer #1 (Remarks to the Author):

In this manuscript, Grob et al. describe characterization of several important enzymes involved in synthesis of a family of cystobactamid (Cys) compounds. The authors designed and assembled a synthetic construct of a Cys biosynthetic gene cluster for heterologous expression in M. xanthus. The authors also created a number of deletion mutations to this construct. Additionally, the authors built a number of single gene expression constructs for heterologous expression in E. coli, purification, and in vitro characterization. Combinatorically and systematically testing the function of these constructs, the authors contributed tremendously to improved understanding of the mechanisms Cys synthesis. Of particular interest, the authors identified an AMDH domain that can catalyze an aminomutase- or an amide dehydratase-type reaction depending on the presence of active CysJ.

The work by Grob et al. is of high quality; their discoveries are very interesting and significantly advance the natural product research field. I heartily recommend the manuscript be accepted for publication in Nature Communications. However, a few things could be improved, as listed below.

Major Improvements Needed

In the MS analyses, I see –5 to +3 Da variations among proteins between calculated and measured masses in Figures 2, 3, and 6. I am not familiar with protein MS analysis, but the variations seem large compared with most other MS-based methods. It would be helpful for the authors to provide possible explanations for the variations.

We provide a potential reason for the variations below. We would like to emphasize that the differences occur in theoretical versus measured mass of the protein species itself and NOT in the mass differences that derive from the chosen biochemical experimental conditions (mass shifts caused by changes introduced by the biochemical reactions under study). The latter are decisive to interpret the experiment. Due to the known potential reasons for these mass differences, we did not add a corresponding section into the manuscript/SI yet, but we are happy to do so if required.

Large molecules like proteins are measured as multiply charged complexes when ionized with electrospray ionization. These charge states in combination with an abundance of isotopic peaks result in highly complex mass spectra that need to be deconvoluted in order to calculate the average neutral mass of the protein. The protein spectra presented in the manuscript were deconvoluted using the Compass DataAnalysis Maximum Entropy algorithm (Bruker). This algorithm is based on image

processing techniques that use Bayesian probability methods to give the most probable molecular mass distribution based on the underlying mass spectra. The resulting values are therefore affected by the parameters, such as resolving power, entered into the algorithm as well as the “region” of the peak that is to be deconvoluted as shown in Figure R1 and Figure R2 below.

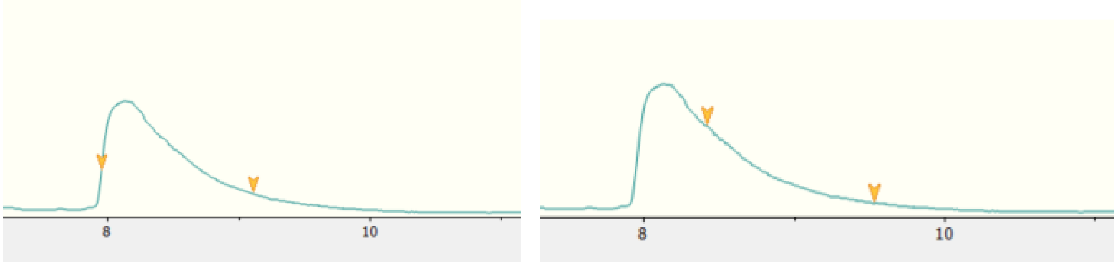

Figure R1|Example for different ranges of the protein peak chosen for deconvolution. Left: 7.98 – 9.15 min. Right: 8.40 – 9.54 min.

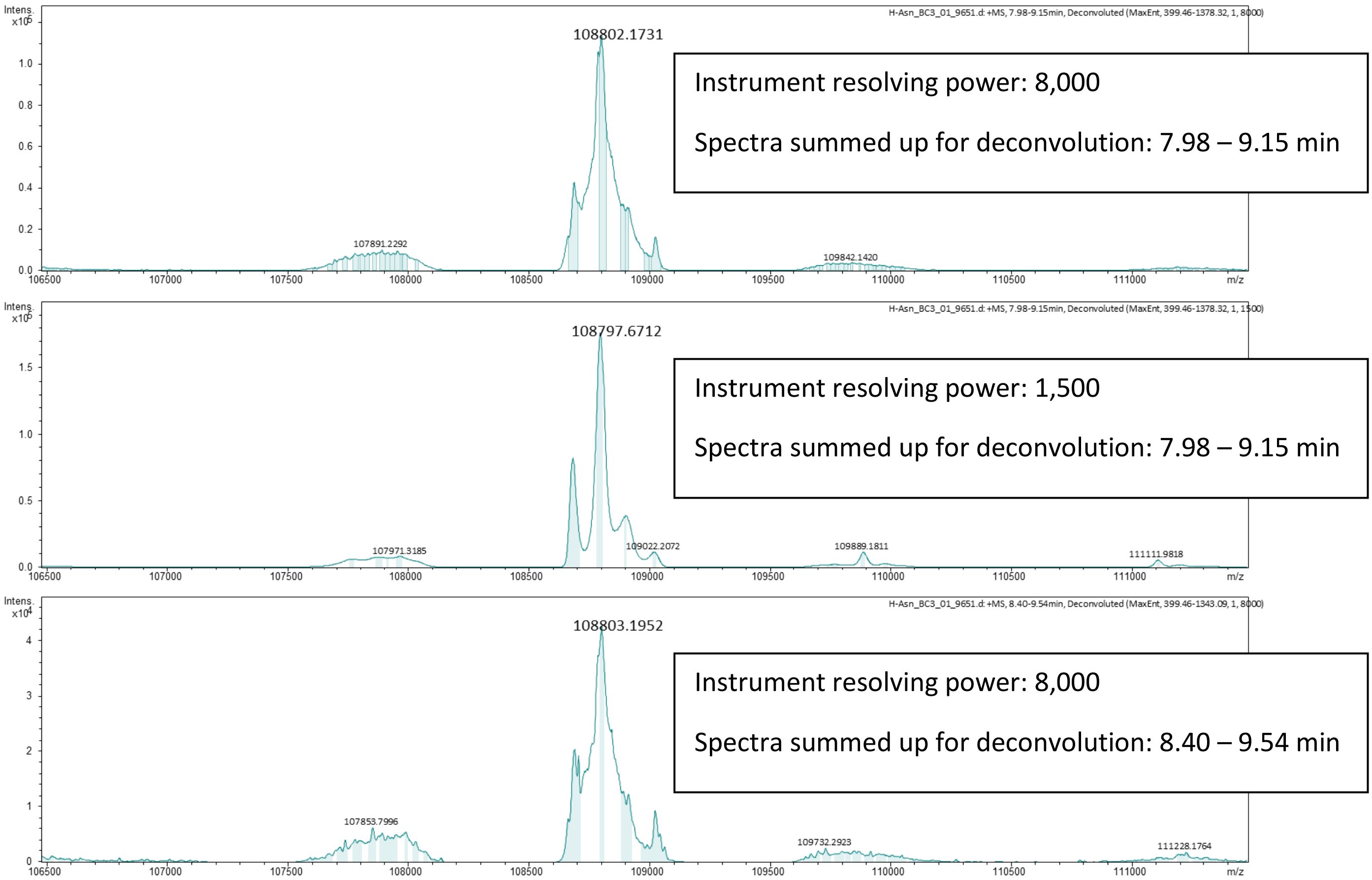

Figure R2|Deconvolution results of sample CysH + L-Asn as seen in Figure 2C (in the manuscript) with different deconvolution parameters and different regions of the original peak chosen for deconvolution.

The accuracy of a measurement is additionally affected by the tool used for calculation of the theoretical average mass of a protein. As isotope ratios vary globally, every tool uses slightly different values with which protein masses are calculated. This can have an effect of about 1 Da / 1 kDa protein as shown in Figure R3 and Figure R4.

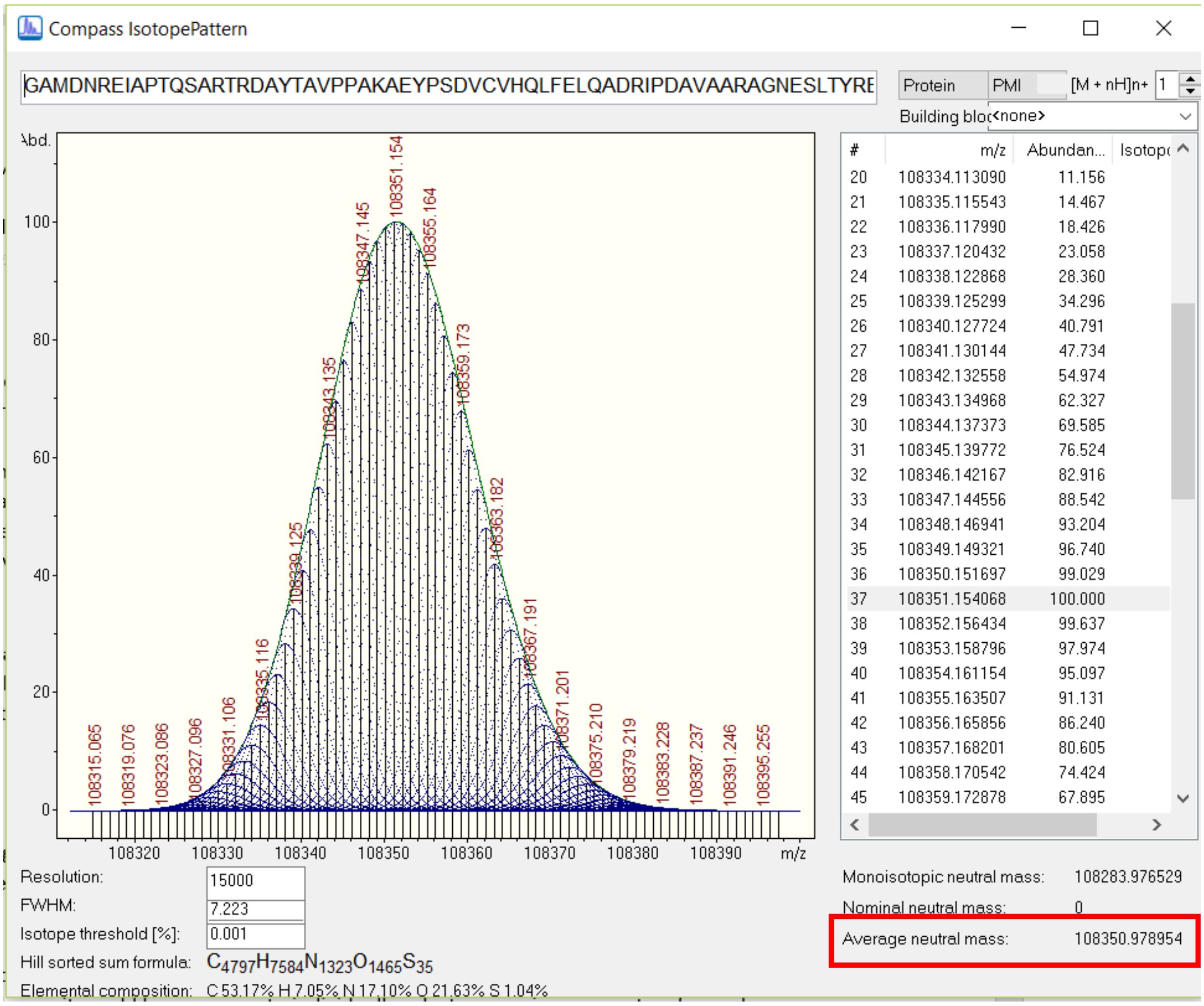

Figure R3|Calculation of the average molecular weight of the *apo* form of CysH with the Compass IsotopePattern tool.

ExPASy Bioinformatics Resource Portal

Compute pI/Mw

Theoretical pI/Mw (average) for the user-entered sequence:

```
        10         20         30         40         50         60
GAMDNREIAP TQSARTRDAY TAVPPAKAEY PSDVCVHQLF ELQADRIPDA VAARAGNESL

        70         80         90        100        110        120
TYRELNFRAN QLARYLVAKG VVPRGSVAVL MNRTPACLVS LLAIIKAGAA YVPVDAGLPA

       130        140        150        160        170        180
KRVDYILTDS GATCVLTDRE TRSLLDEPRS ASTLVIDVDD PSIYSGETSN LGLAVDPEQQ

       190        200        210        220        230        240
VYCIYTSGST GLPKGVMVQH RALMNYVWWA KKQYVTDAVE SFALYSSLSF DLTVTSIFVP

       250        260        270        280        290        300
LISGRCIDVY PDLGEDVPVI NRVLEDNKVD VVKLTPAHLA LLRNTDLSQS RLKVLILGGE

       310        320        330        340        350        360
DLRAETAGDV HKRLDGRAVI YNEYGPTETV VGCMIHRYDP AVDLHGSVPI GVGIDNMRIY

       370        380        390        400        410        420
LLDDRRRPVK PGEVGEIYIG GDGVTLGYKD KPQVTADHFI SNPFVEGERL YASGDLGRVN

       430        440        450        460        470        480
ERGALVFLGR KDLQIKLRGY RIELGEIESA LLSYPGIKEC IVDSTKTAQS QAAAQLTYCT

       490        500        510        520        530        540
KCGLASSFPN TTYSAEGVCN HCEAFDKYRS VVDDYFSTMD ELQSIVTEMK SIHNSKYDCI

       550        560        570        580        590        600
VALSGGKDST YALCRMIETG ARVLAFTLDN GYISEEAKQN INRVVARLGV DHRYLSTGHM

       610        620        630        640        650        660
KEIFVDSLKR HSNVCNGCFK TIYTFAINLA QEVGVKHVVM GLSKGQLFET RLSALFRTST

       670        680        690        700        710        720
FDNAAFEKSL VDARKIYHRI DDAVSRLLDT TCVKNDKVIE NIRFVDFYRY CHASRQEMYD

       730        740        750        760        770        780
YIQERVGWAR PIDTGRSTNC LLNDVGIYVH NKERRYHNYS LPYSWDVRMG HISREEAMRE

       790        800        810        820        830        840
LDDSADIDVE RVEGIIKDLG YELNDQVVGS AEAQLVAYYV SAEEFPASDL RQFLSEILPE

       850        860        870        880        890        900
YMVPRSFVQL DSIPLTPNGK VNRQALPKPD LLRKAGTDGQ AAPRTPVEKQ LAELWKEVLQ

       910        920        930        940        950        960
VDSVGIHDNF FEMGGHSLPA LMLLYKIDSQ FHKTISIQEF SKVPTISALA AHLGSDTEAV

       970
PPGLGEVVDQ SAPAYRG
```

Theoretical pI/Mw: 5.53 / 108352.06

Figure R4|Calculation of the average molecular weight of the *apo* form of CysH with the ExPASy tool.

In some of the analyses the mass delta might also be a result of unspecific reactions on the protein. These result in several protein species in masses so close to the main peak that the algorithm is not able to resolve them properly (or not at all) depending on the value entered for resolving power (Figure 2). These extra protein species likely also affect the measured mass of the target protein by 1-2 Da.

We believe a combination of these effects is responsible for the delta between measured and theoretical mass. In order to mitigate these problems and to minimize this delta, it would be necessary to individually select processing parameters, region of deconvolution and theoretical mass calculation tools for each measurement based on the desired outcome. To avoid biasing our analyses towards what we want to measure instead of what we actually measured, we chose parameters that gave good results on average and decided to keep them consistent across all measurements.

To validate the results, the authors need to provide supplementary information on how they identified the structures of the 18 new Cys derivatives that were found through the studies.

We added another section to the SI, named "Identification of the new cystobactamid derivatives." with the following explanation, one example and a Supplementary Figure:

"We identified a total of 22 new cystobactamid derivatives (13 natural and 9 unnatural) based on high-resolution masses and the MS_2 fragmentation patterns. The fragment ions that we used to identify the structural differences of the new derivatives, including the respective calculated fragment masses, are shown in Supplementary Figure 6 and Supplementary Table 10. We only focused on the three fragments a, b and c, because fragment a only contains the N-terminal cystobactamid part (*p*NBA_1-*p*ABA_2), whereas fragment b contains the N terminal part plus the linker and one additional *p*ABA (*p*NBA_1-*p*ABA_2-linker-*p*ABA_3). Fragment c one additional (tailored) *p*ABA (*p*NBA_1-*p*ABA_2-linker-*p*ABA_3-*p*ABA_4) compared to fragment b. Those three fragments were also described previously by Baumann and coworkers (in the SI).[6] We decided to look at those three fragments, because it enabled us to find structural differences in the N-terminal part, e.g. after deletion of *cysR* (*m/z* 269.0562 → 239.0821 $[M+H]^+$; NO_2 → NH_2), in the linker, e.g. after deletion of *cysQ* (*m/z* 532.1468 → 518.1312 $[M+H]^+$; methoxy-L-(iso)Asn → hydroxy-L-(iso)Asn), and in the C-terminal part, e.g. *m/z* 725.2207 → 711.2051 $[M+H]^+$; isopropoxyl-*p*ABA_4 → ethoxy-*p*ABA_4).

The differences between experimental masses and the calculated masses of the new derivatives (and their corresponding fragments) are listed in Supplementary Table 10. Based on the high-resolution mass of the new derivatives and their fragmentation pattern we were able to propose the structures (Supplementary Figure 7) using known cystobactamids as template (example depicted in Supplementary Figure 14).

E.g. after the deletion of *cysQ*, the gene encoding the *O*-methyltransferase that acts on the linker, and heterologous expression of the deletion construct in *M. xanthus* DK1622, we did not find the two major cystobactamid peaks (Cys919-1 and Cys919-2) in the EIC with *m/z* 920.3 $[M+H]^+$. Instead we found two new masses with *m/z* 906.3004 (Cys905-1c) and 906.3007 (Cys905-2c) [M+H]+ (red box in Supplementary Figure 14). The fragmentation pattern shows that fragment a (blue box), which is the N-terminal cystobactamid part (pNBA$_1$-pABA$_2$) of Cys905-1c/2c is the same as for Cys919-1/2. However, fragment b (green box) differs when comparing Cys919 with Cys905, as there is a mass shift of 14 Da (*m/z* 532.1468 → 518.1312 $[M+H]^+$), indicating that a methyl group is missing in Cys905 (structural difference shown in red). In fragment c (yellow box) and in the mass of the molecular ion (red box) the mass shift of 14 Da is still present, which means that no further structural difference is found between Cys919 and Cys905. Assignment of the linker moieties with the same masses (e.g. linker G and F for Cys905-1c and Cys905-2c, respectively) was done based on the different retention times that were also observed for previously reported cystobactamids, in which derivatives harboring L-isoasparagine linkers eluted between 1.4 and 1.6 min earlier than derivatives harboring L-asparagine linkers.

However, for four unnatural cystobactamids, we were not able to propose a putative structure: Cys905-2d, Cys905-2e, Cys919-2b1 and Cys919-2b2. In *M. xanthus* DK1622 pMYC20Cys_v4Δ*cysJ* and *M. xanthus* DK1622 pMYC20Cys_v4Δ*cysJ*ΔAMDH we found the two new minor Cys905 derivatives, Cys905-2d and Cys905-2e (Supplementary Figure 15). The retention times are different to the one observed for Cys905-1c/2c (major products in the extract of M. xanthus DK1622 pMYC20Cys_v4Δ*cysQ*), but surprisingly, the fragmentation pattern is the same (Supplementary Table 10). Since we found Cys905-2d and Cys905-2e in the extracts of the cysJ and the cysJ-AMDH deletion strains, we speculate that the linker is an L-asparagine rather than an L-isoasparagine derivative, because L-isoasparagine linkers only occur if the AMDH domain is present. In the *cysJ* deletion strains we expected the production of exclusively desmethoxylated linker derivatives (fragment b: *m/z* 502.1363 [M+H]+).

However, the fragment ion b has an *m/z* of 518.1312 [M+H]+, which means that fragment b potentially contains an additional hydroxyl group even though *cysJ* was deleted in the producer strains. Since the retention times are different compared to Cys905-1c/2c, we can only hypothesize that the hydroxylation occurs in a different position and that it might be catalyzed by a different enzyme encoded in the genome of *M. xanthus* DK1622. However, this enzyme must be active also in the other strains harboring different construct. Thus, we searched for cystobactamid derivatives with double hydroxylated fragment b ions, but we did not find any of them. Finally, purification and subsequent NMR analysis would be required to understand where the hydroxylation in Cys905-2d/e occurs and why those two derivatives have a different retention time despite the same potential linker moiety. However, due to the very low production titer, this is not possible at this time.

Furthermore, we found two new Cys919 derivatives, Cys919-2b1 and Cys919-2b2 (Supplementary Figure 15), in the extract of the AMDH deletion strain. Both derivatives show the same fragmentation pattern compared to each other, but they have a different fragmentation pattern compared to Cys919-2 (Supplementary Table 10). The fragmentation pattern shows that the linker is desmethylated (fragment b: *m/z* 518 [M+H]+ compared to *m/z* 532 [M+H]+ for Cys919-2), because CysQ is not able to operate in case the AMDH domain is deleted. Since fragment c has a difference of 14 Da compared to fragment c of Cys919-2, the additional methyl group has to be located in the terminal pABA5 moiety. We suggest that R2 (see Supplementary Figure 6 and Supplementary Table 10) is a 1-methylpropoxy moiety as this moiety has already been described in native cystobactamids. However, since we identified two derivatives with only slightly different retention times (0.22 min), the methyl group might also be located elsewhere or at different positions in Cys919-2b1 and Cys919-2b2. We also cannot exclude the possibility that there is another structural difference elsewhere in the molecule, which we cannot explain at this time and only based on MS2 data. As for Cys905-2d/e, we would also have to isolate those derivatives, which is not possible due to the very low production titers.”

Compound	Linker	R_1	R_2	R_3	calculated *m/z* $[M+H]^+$	measured *m/z* $[M+H]^+$	ret. Time [min]	Fragment a *m/z* calculated	Fragment a *m/z* measured	Fragment b *m/z* calculated	Fragment b *m/z* measured	Fragment c *m/z* calculated	Fragment c *m/z* measured
natural cystobactamid derivatives													
Cys919-1	E	*i*PrO	*i*PrO	H	920.3097	920.3090	9.06		269.0552		532.1452	725.2207	725.2190
Cys919-2	A	*i*PrO	*i*PrO	H		920.3097	10.67		269.0553		532.1451		725.2185

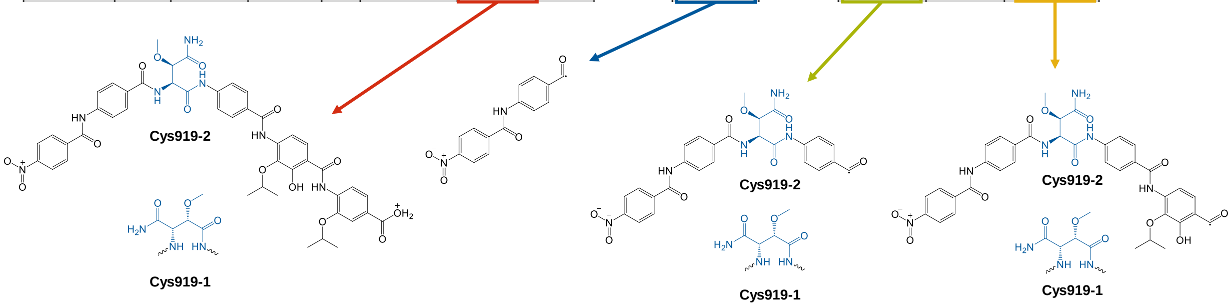

unnatural cystobactamid derivatives													
Cys905-1c	G	*i*PrO	*i*PrO	H	906.2941	906.3004	8.77	269.0562	269.0578	518.1312	518.1337	711.2051	711.2092
Cys905-2c	F	*i*PrO	*i*PrO	H		906.3007	10.30		269.0576		518.1342		711.2090

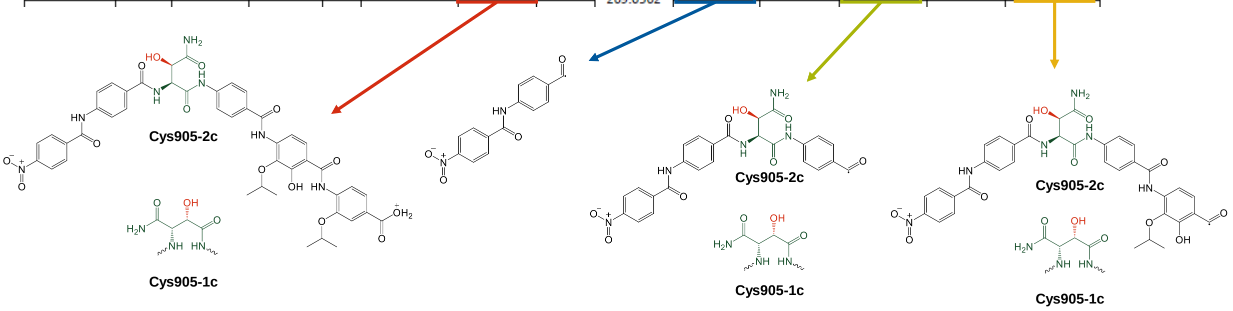

Supplementary Figure 14|Example of how structures were assigned to new cystobactamid derivatives. On top a cropped part of Supplementary Table 10 with the example Cys919-1 and Cys919-2 is shown. The measured high-resolution mass of the molecular ions is shown in the red box. The corresponding structures are shown below. The fragment ion masses are shown in blue, green and yellow boxes with the corresponding fragment ions below, correspondingly. After deletion of cysQ, Cys905-1c and Cys905-2c were produced instead of Cys919-1 and Cys919-2. The measured high-resolution masses of the molecular ion and the fragment ions are shown in the red, blue, green and yellow boxes. The corresponding structures are shown below. The difference of 14 Da in the molecular ion and fragments b and c is due to a loss of a methyl group in the linker, which led to the assignment of a hydroxyl group (labelled in red) instead of a methoxy group. The linker type was assigned based on the retention time difference, whereas derivatives with L-isoasparagine linkers eluted 1.4–1.6 min earlier than derivatives with L-asparagine linkers.

For Figures 4 and 5, the authors should provide full LC-MS EIC traces in order to fully validate the results (i.e., 427.1 for CysH + L-Asn [or isoAsn] and 411.1 for CysH + CysJ + L-Asn [or isoAsn] for Figure 4, and EIC 920.3, 906.3, 890.3, and 872.3 traces for all experiments).

We provided full LC-MS EIC traces in Figure 4 (shown below).

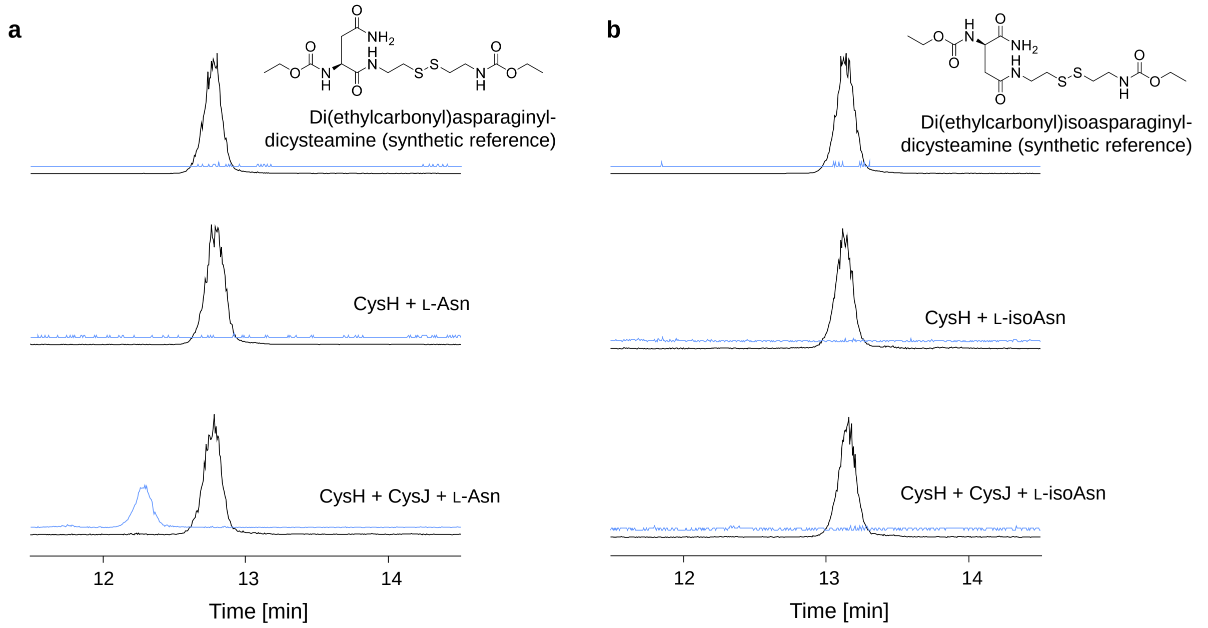

Figure 4|Hydroxylation of CysH-bound L-asparagine by CysJ. HPLC-MS analysis of cysteamine-unloaded and derivatized substrate from the CysH protein.

For Figure 5, we searched for all those EICs in every extract and we identified another four unnatural cystobactamids, which we have not previously seen. In *M. xanthus* DK1622 pMYC20Cys_v4Δ*cysJ* and *M. xanthus* DK1622 pMYC20Cys_v4Δ*cysJ*ΔAMDH we found two new minor Cys905 derivatives (with very low peak intensity), which we named Cys905-2d and Cys905-2e (see modified Figure 5 below; labelled with #).

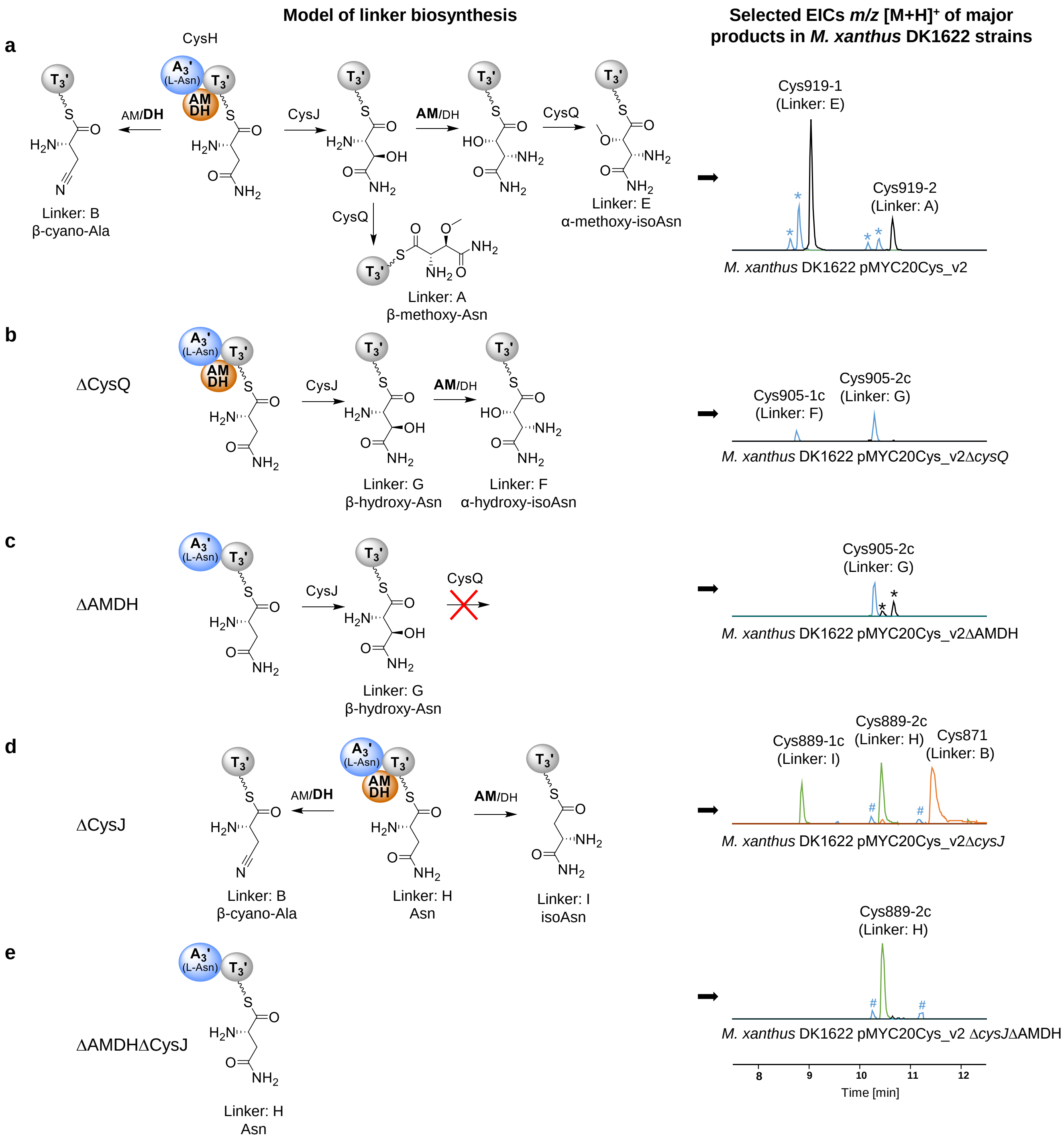

Figure 5 | Summary of biosynthesis pathways for cystobactamid linkers.

The retention times of Cys905-2d and Cys905-2e are different to the one observed for Cys905-1c/2c (major products in the *M. xanthus* DK1622 pMYC20Cys_v4Δ*cysQ* strain), but surprisingly, the fragmentation pattern is the same (see modified Supplementary Table 10 in SI and see below).

Producer	Compound	Linker	R_1	R_2	R_3	calculated m/z [M+H]$^+$	measured m/z [M+H]$^+$	ret. Time [min]	Fragment a m/z calculated	Fragment a m/z measured	Fragment b m/z calculated	Fragment b m/z measured	Fragment c m/z calculated	Fragment c m/z measured
	**natural cystobactamid derivatives**													
produced in *M. xanthus* DK1622 with entire cluster	Cys905-1a	E	*i*PrO	EtO	H	906.2941	906.2935	8.65		269.0556	[illegible]	532.1453	[illegible]	725.2192
	Cys905-1b	E	EtO	*i*PrO	H		906.2943	8.83		269.0555		532.1455	711.2051	711.2033
	Cys905-2a	A	EtO	*i*PrO	H		906.2923	10.18		269.0551		532.1439		711.2028
	Cys905-2b	A	*i*PrO	EtO	H		906.2932	10.40		269.0553		532.1451		725.2182
	**unnatural cystobactamid derivatives**													
M. xanthus DK1622 pMYC20Cys_v4Δ*cysQ*	Cys905-1c	G	*i*PrO	*i*PrO	H	906.2941	906.3004	8.77	269.0562	269.0578	518.1312	518.1337	711.2051	711.2092
	Cys905-2c	F	*i*PrO	*i*PrO	H		906.3007	10.30		269.0576		518.1342		711.2090
M. xanthus DK1622 pMYC20Cys_v4Δ*cysJ*	Cys905-2d	?	*i*PrO	*i*PrO	H		906.2928	10.24		269.0555		518.1298		711.2025
	Cys905-2e	?	*i*PrO	*i*PrO	H		906.2917	11.15						

Thus, we conclude that Cys905-2d and Cys905-2e are structural isomers of Cys905-2c. Since we found Cys905-2d and Cys905-2e in the extracts of the *cysJ* and the *cysJ*-AMDH deletion strains, we speculate that the linker is an L-asparagine rather than an L-isoasparagine derivative, because L-isoasparagine linkers only occur if the AMDH domain is present. In the *cysJ* deletion strains we expected the production of exclusively desmethoxylated linker derivatives (fragment b: m/z 502.1363 [M+H]$^+$). However, the fragment ion b has an m/z of 518.1312 [M+H]$^+$, which means that fragment b potentially contains an additional hydroxyl group even though *cysJ* was deleted in the producer strains. Since the retention times are different compared to Cys905-1c/2c, we can only hypothesize that the hydroxylation occurs in a different position and that it might be catalyzed by a different hydroxylase or P450 enzyme, of which plenty are encoded in the genome of *M. xanthus* DK1622. However, this enzyme must be active also in the other strains harboring different construct. Thus, we searched for cystobactamid derivatives with double-hydroxylated fragment b ions, but we did not find any of them. Based on these findings, we speculate that this hydroxylase is only active when the primary hydroxylation by CysJ does not occur. Finally, purification and subsequent NMR analysis would be required to understand where the hydroxylation in Cys905-2d/e occurs and why those two derivatives have a different retention time despite the same potential linker moiety. However, due to the very low production titer, this is not possible at this time.

Furthermore, we found two new Cys919 derivatives, Cys919-$2b_1$ and Cys919-$2b_2$ (labelled with black asterisks in panel **c**), in the extract of the AMDH deletion strain. Both derivatives show the same fragmentation pattern compared to each other again arguing for them being isomers, but they have a different fragmentation pattern compared to Cys919-2 (see below).

Producer	Compound	Linker	R_1	R_2	R_3	calculated m/z [M+H]$^+$	measured m/z [M+H]$^+$	ret. Time [min]	Fragment a m/z calculated	Fragment a m/z measured	Fragment b m/z calculated	Fragment b m/z measured	Fragment c m/z calculated	Fragment c m/z measured
	**natural cystobactamid derivatives**													
M. xanthus DK1622 entire cluster	Cys919-2	A	*i*PrO	*i*PrO	H	[illegible]	920.3097	10.67		269.0553		532.1451	[illegible]	725.2185
	**unnatural cystobactamid derivatives**													
M. xanthus DK1622 pMYC20Cys_v2ΔAMDH	Cys919-$2b_1$	A	*i*PrO	1-MePrO	H	920.3058	920.3083	10.45	[illegible]	269.0555		518.1310		711.2025
	Cys919-$2b_2$	A	*i*PrO	1-MePrO	H	920.3058	920.3086	10.67		269.0557		518.1294		711.2036

The fragmentation pattern shows that the linker is desmethylated (fragment b: m/z 518 [M+H]$^+$ compared to m/z 532 [M+H]$^+$ for Cys919-2), because CysQ is not able to operate in case the AMDH domain is deleted. Since fragment c has a difference of 14 Da compared to fragment c of Cys919-2, the additional methyl group has to be located in the terminal $pABA_5$ moiety. We suggest that R_2 (see

Supplementary Figure 6 or Supplementary Table 10) is a 1-methylpropoxy moiety as this moiety has already been described in native cystobactamids. However, since we identified two derivatives with slightly different retention times (0.22 min), the methyl group might also be located elsewhere or at different positions in Cys919-2b$_1$ and Cys919-2b$_2$. We also cannot exclude the possibility that there is another structural difference elsewhere in the molecule, which we cannot explain at this time and the assignment is only based on MS^2 data. As for Cys905-2d/e, we would also have to isolate those derivatives, which is not possible due to the very low production titers.

In our opinion Figure 5 looks overloaded if we show all EICs in the "overlaid" mode and the figure description will be very complex. E.g. we found Cys905 derivatives (Cys905-1a, Cys905-1b, Cys905-2a and Cys905-2b) in the extract of *M. xanthus* DK1622 pMYC20Cys_v2 (entire gene cluster without any gene deletions; four blue peaks shown in panel **a**) as minor derivatives. Those derivatives have different fragmentation patterns and retention times compared to the major Cys905-1c/2c derivatives produced in the *cysQ* deletion strain (panel **b**). The same is for the newly identified Cys905-2d and Cys905-2e derivatives produced in the *cysJ* and *cysJ*/AMDH deletion strains (discussed above). We would have to explain the appearance of all those minor derivatives, but we think the figure is already complex enough and this would hinder the understanding of the whole story. Therefore, we suggest only showing the major derivatives in manuscript Figure 5 (as it has been shown before) and show (+ discuss) the full LC-MS EIC traces in the SI. Thus, we added respective texts in the manuscript (see below) and in the SI section "Identification of the new cystobactamid derivatives". Additionally, we added Supplementary Figure 15, which is shown below.

Manuscript text:

"Notably, after deletion of *cysJ* or the AMDH domain we also identified four minor unnatural cystobactamids for which we were not able to propose putative structures (see Supplementary Information; Supplementary Figure 15)."

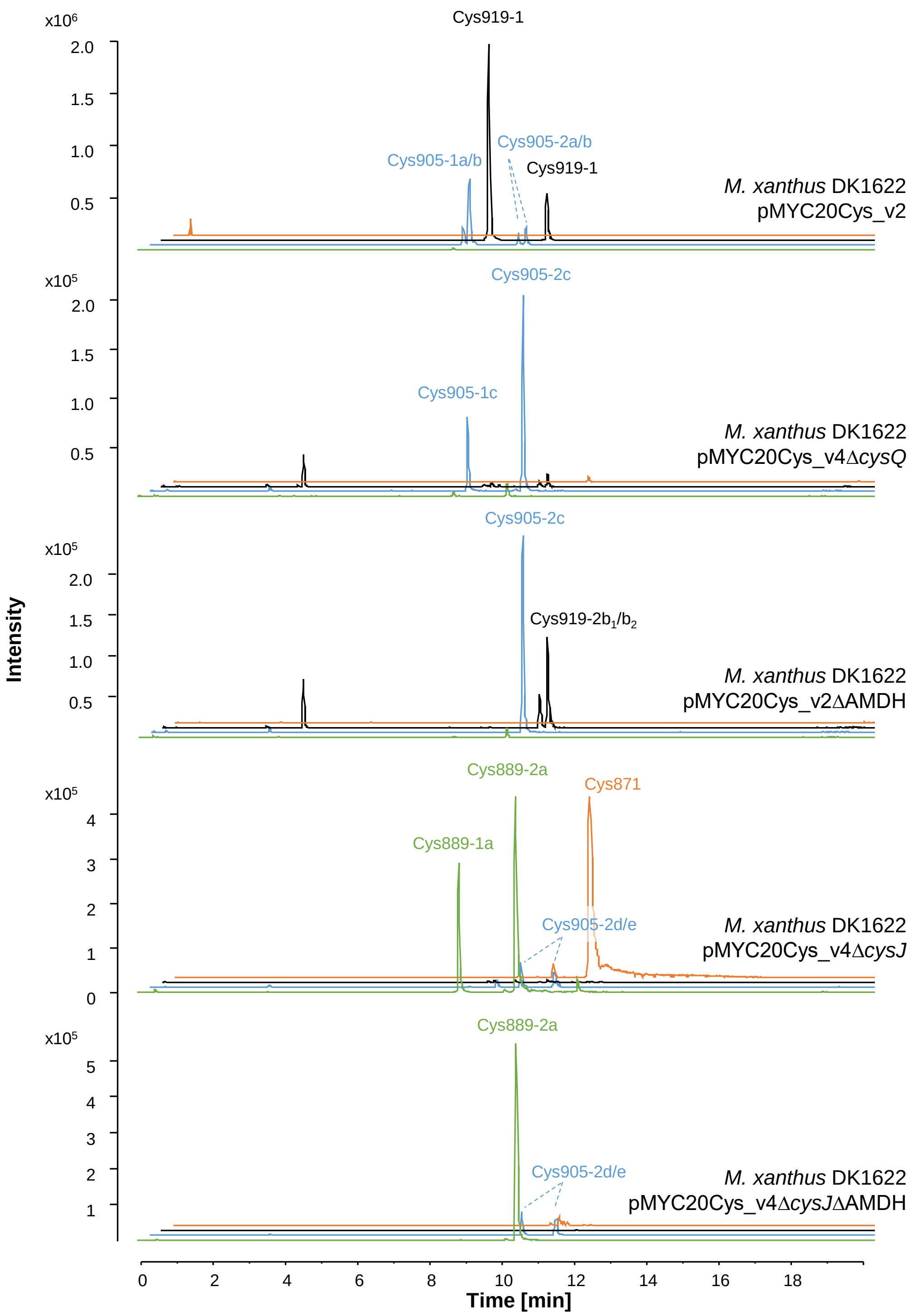

Supplementary Figure 15|Full HPLC-ESI-MS EIC traces of methanolic extracts from different heterologous strains. Stacked/overlayed view of the EICs *m/z* 920.3 $[M+H]^+$ (black), 906.3 (blue), 890.3 (green) and 872.3 (orange).

The authors can elaborate the explanation how the product selectivity toward Cys905-2c over Cys889-1c and Cys871 is decided. Is it kinetically driven? Is the presence of CysJ important even if it is inactive?

Did you mean why the isomerization of L-asparagine to L-isoasparagine is less abundant in the CysJ deletion strain as compared to other strains with active CysJ? This leads to a higher production of Cys889-2a compared to Cys889-1a (we renamed the compounds from c → a; as it was shown in Table 1). Usually cystobactamids of series 1 (L-isoasparagine linker) are more abundant (analyzed based on peak intensity), but after deletion of CysJ the ratio was the other way round and the production of Cys871 increased substantially. This was described on p. 16-17, lines 259-265.

We speculate that CysH, CysJ and CysQ form a protein complex, because CysJ is an *in trans*-acting enzyme and CysQ was shown to be inactive after deletion of the AMDH domain on CysH. If CysJ is active, β-hydroxylation of L-asparagine by CysJ occurs much faster than dehydration of L-asparagine by the AMDH domain. This would explain why we did not find Cys871 in the extract of the strain harboring the entire cluster. However, after deletion of CysJ, Cys871 was produced in relatively high amounts. We assume that in this setting, there is more time for the AMDH domain to dehydrate L-asparagine, because it is not β-hydroxylated anymore. We believe that this part of the reaction is kinetically driven. The question remains why the isomerization of L-asparagine to L-isoasparagine is less efficient in the CysJ deletion strain. We provide two possible explanations for this finding: First, isomerization occurs after β-hydroxylation of L-asparagine in a more efficient way, because the AMDH domain favors β-hydroxyl-L-asparagine over L-asparagine. Second, the protein-protein interactions between CysH and CysJ influence the reactivity of the AMDH domain. If CysJ is not present, isomerization may occur slower, thus reducing the relative amount of isomerized product compared to non-isomerized product. We added respective explanations on p. 16/17 in the manuscript:

"Consequently, we hypothesize that, in presence of CysJ, β-hydroxylation of L-asparagine occurs much faster than dehydration of L-asparagine by the AMDH domain."

[…]

"We speculate that the isomerization of β-hydroxyl-L-asparagine by the AMDH domain is more efficient than the isomerization of L-asparagine, or that CysH, CysJ and CysQ form a protein complex influencing the reactivity of the AMDH domain. If CysJ is deleted, the isomerization reaction may occur much slower, thus leading to a decreased L-isoasparagine/L-asparagine linker ratio and an increased probability of L-asparagine dehydration."

I understand that that the authors' attempt to crystallize the AMDH domain failed. Alternatively, the authors are recommended to mutate some of the conserved motifs listed in Supplementary Figure 10 and see how AMDH activity changes.

We had tried to introduce single point mutations into conserved core motifs of the AMDH domain in the past and we have been trying it anew for the last three months. However, until now we failed to clone the desired constructs. This is not unexpected as in our experience the introduction of single point mutations into expression constructs harboring highly repetitive regions of this size (>60 kb) is a challenging task.

The first cloning strategy we pursued was the replacement of the AMDH domain by a kanamycin selection marker, which is flanked by *Bsa*I restriction sites, using Red/ET recombineering (generating pMYC20Cys_v2ΔAMDH-kanR, pMYC20Cys_v4Δ*cysQ*ΔAMDH-kanR and pMYC20Cys_v4Δ*cysJ*ΔAMDH-kanR). Next, we hydrolyzed the resulting constructs with *Bsa*I to remove the selection marker. Instead of subsequently re-ligating the constructs (as we did it to obtain targeted gene deletions), we tried to ligate a mutated AMDH domain into it, which we generated by PCR using mutagenic primers. In total we prepared four independent AMDH domain products with point mutations at different positions and we planned to clone them into pMYC20Cys_v2, pMYC20Cys_v4Δ*cysQ* and pMYC20Cys_v4Δ*cysJ*. However, this final ligation step did not work for any of the constructs so far. The ligation of relatively small DNA fragments (1.7 kb) into very large constructs (> 60 kb) is difficult and goes along with super low cloning efficacy.

Thus, we developed a second cloning strategy to circumvent the inefficient ligation step. We fused the mutated AMDH domain products to an ampicillin resistance cassette by PCR and tried to introduce this newly obtained product into pMYC20Cys_v2ΔAMDH-kanR, pMYC20Cys_v4Δ*cysQ*ΔAMDH-kanR and pMYC20Cys_v4Δ*cysJ*ΔAMDH-kanR via Red/ET recombineering, thereby replacing the kanamycin selection marker. This cloning step did not work for several weeks, probably because of random recombination events caused by the repetitive sequence segments which span over large parts of the gene cluster. In case the cloning step was successful, we would only have to hydrolyze the resulting constructs with *Bsa*I and re-ligate them. With this strategy, we do not rely on the inefficient ligation step to introduce the mutated AMDH domain, but the mutated AMDH domain is introduced by Red/ET recombineering.

At this time, we cannot foresee how much time the remaining cloning steps will take and if they will eventually work at all. Furthermore, we are also still trying to crystallize the AMDH domain to gain more insight into its functionality. Therefore, we believe that both the mutation of conserved core motifs and the crystal structure of the AMDH domain will be the focus of an additional publication.

Minor Improvements Needed

Pg 3, Figure 1. Because R1 and R2 only refer to MeO and OH, respectively, the authors could simply incorporate them as part of the Albicidin structure.

Done.

Pg 3, Line 43. It would be helpful if the authors refer to Table 1.

Done.

Pg 9, Table. Is this part of Table 1 or Table 2? The titles of the first two columns are different from those of Table 1.

This table is part of Table 1. We changed the titles and the construct labelling and we hope it is more clearly visible now.

Pg 9, Table. Should Cyc899-1a and Cys899-2a be Cys899-1c and Cys899-2c?

We changed the naming of the derivatives several times during the writing process. Cys889-1a/2a is correct, but we forgot to change the other Cys889-1c/2c in some parts of the text. Thank you for finding this mistake.

Pg 13, Line 199. Please specify which supplementary information is referred to (I assumed it is Supplementary Table 14).

Done.

Pg 13, Line 210. Please specify which supplementary information is referred to (I assumed it is Supplementary Table 15).

Done.

The labels in Supplementary Figure 3 is too small.

Done.

Reviewer #2 (Remarks to the Author):

The paper by Groß et al desribes details in the biosynthesis of the potent antibiotic cystobactamid and includes compound identification of several novel derivatives, in vitro reconstitution of important steps and branching points in the biosynthesis and describes the biochemical function of a new domain in NRPS that has aminomutase and amide dehydratase activity.

This is a complex but excellent paper with lots of new details that shows how far one can go even with complex natural products with respect to analysis and biochemistry and it must be published. However, I have a few suggestions that might help to even improve it:

For NRPS introduction, please also cite Mainz and Süßmuth, the latest and most comprehensive review in the field

Done.

For all MS analysis data: Did the authors also perform Papst ejection assays?

Did you mean Ppant ejection assay? We only carried out unloading assays coupled with derivatization of the analyte to investigate the hydroxylation and isomerisation as reported in Figure 4. We agree that Ppant ejection assays may be an alternative method to potentially obtain even more precise data; however, the complexity of such assays including the chemical synthesis of the necessary reference substances led us to favor the use of intact protein MS for analysis of the reactions.

Fig 3g: delta in protein name

Done.

Fig. 3: How ist the isomerization in f (last step) confirmed?

We were not able to confirm the isomerization from (β-hydroxy-)L-asparagine to (α-hydroxy-)L-isoasparagine by protein MS, since the exact masses of both substrates are identical and the fragmentation patterns do not allow their differentiation either. In the cysteamine unloading assay (see Figure 4) we were only able to synthesize Di(ethylcarbonyl)-L-(iso)asparaginyl-dicysteamine references; however, we did not strive for the synthesis of hydroxylated references since too many synthesis steps would be required. In the cysteamine unloading assay we could show that CysJ hydroxylated the CysH-bound L-asparagine, but we were not able to show if the hydroxylated product was indeed β-hydroxy-L-asparagine or α-hydroxy-L-isoasparagine as no reference compound was available. Since we could not confirm isomerization in this set of experiments (p. 13, lines 214-216), we showed the isomerization step in Figure 3f in brackets. However, we eventually confirmed the isomerization reaction by the AMDH domain after its targeted deletion from CysH (see Figure 5), which

was easier to achieve as compared to the synthesis of hydroxylated references for the cysteamine unloading assay.

We added the following explanation in the figure description of Figure 3:

"**f:** CysH incubated with L-asparagine and CysJ leads to loading of the substrate onto CysH with subsequent hydroxylation by CysJ (see Figure 4). The isomerization of (β-hydroxy-)L-asparagine to (α hydroxy-)L-isoasparagine is shown in parenthesis, because this step cannot be observed by MS. The isomerization was confirmed by deletion of the AMDH domain from the BGC and analysis of the production profile after heterologous expression of the respective construct in *M. xanthus* DK1622 (see Figure 5)."

Line 200: be more specific where to find the data in the supplementary information

Done.

Fig. 6: In order to show shuttling the arrows should be directional not bidirectional as some of them are?

We exchanged most of the bidirectional arrows against directional ones. However, we used bidirectional arrows to show the transfer of L-asparagine from CysH to CysB, because we hypothesized this reaction to be reversible (p. 20, lines 341-343).

Line 342: the sentence with the reversible reaction is unclear to me. Isn't in Fig. 6b twice the same result just with a different pH? Please explain in more detail.

We first observed incomplete loading of CysB with L-asparagine from CysH at a pH of 6.8, because we only detected a small peak of L-asparagine-loaded CysB compared to a big peak of "empty" CysB. When we changed the reaction conditions to pH 8.0, the equilibrium between CysH- and CysB-loaded L-asparagine shifted, but still unloaded CysB was detected. On the contrary, in the second part of the shuttling reaction the transfer of L-asparagine from CysB to CysK-M3 was almost stoichiometric. Thus, we speculated that the first part of the shuttling process is reversible since we observed a reaction condition-dependent dynamic equilibrium but never a full loading of CysB. We showed the same reaction at different reaction conditions to highlight that the equilibrium is dynamic and the incomplete loading of CysB was not the consequence of just one reaction with possibly inappropriate reaction conditions.

We made the following changes in the manuscript on p. 20, lines 340-343 (changes shown in blue here):

Protein MS analysis showed that CysB was only partially loaded with L-asparagine in the presence of CysH, because the major peak still derived from unloaded CysB, whereas no free L-asparagine was loaded (Figure 6b). To exclude that the partial loading is caused by inappropriate reaction conditions, we tested different pH values. Even though the equilibrium between L-asparagine-loaded CysH and CysB shifted pH-dependently, we still observed partial loading of CysB. Thus, we conclude that the reaction is reversible.

The data for the shortened derivative Cys507 is missing and should be added to the SI

Done.

Line 437 and elsewhere: Since the authors have some of the main compounds isolated (also from previous experiments), it should be possible to give the amounts based as MS quantification with the major compound as standard.

We previously did a relative MS-based quantification to get a rough estimation of the production titers of all derivatives. We cultivated 2-3 clones per heterologous producer strain (each clone cultivated only once) and analyzed the extract two times using ESI-HRMS. The peak surface area was integrated manually based on the EIC for the respective derivative. We then compared the average peak surface area of the best producing clones for each derivative with the Cys919-1 production in *M. xanthus* DK1622 pMYC20Cys_v2 (see Table R1).

Table R1|Relative production of all cystobactamid derivatives.

Strain/construct	Derivative	Rel. production compared to Cys919-1 [%]
	**natural cystobactamids**	
	Cys919-1	100
	Cys919-2	28.5
	Cys861-1	1.9
	Cys861-2	2.9
	Cys877-1	5.3
	Cys877-2	6.4
	Cys891-1a	3.9
	Cys891-1b	43.0
	Cys891-2a	3.1
M. xanthus DK1622 pMYC20Cys_v2	Cys891-2b	21.3
	Cys905-1a	9.7
	Cys905-1b	36.2
	Cys905-2a	5.3
	Cys905-2b	10.4
	Cys933-1a	2.5
	Cys933-1b	4.9
	Cys933-2b1	1.0
	Cys933-2b2	0.9
	Cys935-1	3.6
	Cys935-2	10.0
M. xanthus DK1622 pMYC20Cys_v4Δ*cysJ*	Cys871	88.1

Strain/construct	Derivative	Rel. production compared to Cys919-1 [%]
**unnatural cystobactamids**		
M. xanthus DK1622 pMYC20Cys_v4Δ*cysQ*	Cys905-1c	3.1
	Cys905-2c	10.1
M. xanthus DK1622 pMYC20Cys_v2ΔAMDH		16.1
M. xanthus DK1622 pMYC20Cys_v4Δ*cysJ*	Cys905-2d	4.1
	Cys905-2e	6.7
M. xanthus DK1622 pMYC20Cys_v4ΔAMDH	Cys919-2b$_1$	1.9
	Cys919-2b$_2$	5.8
M. xanthus DK1622 pMYC20Cys_v4Δ*cysJ*	Cys889-1a	22.9
	Cys889-2a	49.3
M. xanthus DK1622 pMYC20Cys_v4Δ*cysJ*ΔAMDH		47.8
M. xanthus DK1622 pMYC20Cys_v4Δ*cysR*	Cys889-1b	73.4
	Cys889-2b	89.0

All derivatives exhibit different structural features, which may result in different ionization efficiencies in ESI-HRMS experiments (see e.g. https://pubs.acs.org/doi/10.1021/ac902856t). Thus, the peak surface areas that we integrated to estimate the relative production is probably not relative to the amount of compound actually produced. Furthermore, the numerous cystobactamid derivatives elute at different retention times (Figure R5), meaning that some cystobactamids co-elute with other compounds while other cystobactamids do not co-elute. This may result in ion supression (see e.g. https://doi.org/10.1016/j.talanta.2013.03.048) and reduced signal intensities for the derivatives, which co-elute with other compounds (e.g. peak 5 in Figure R5).

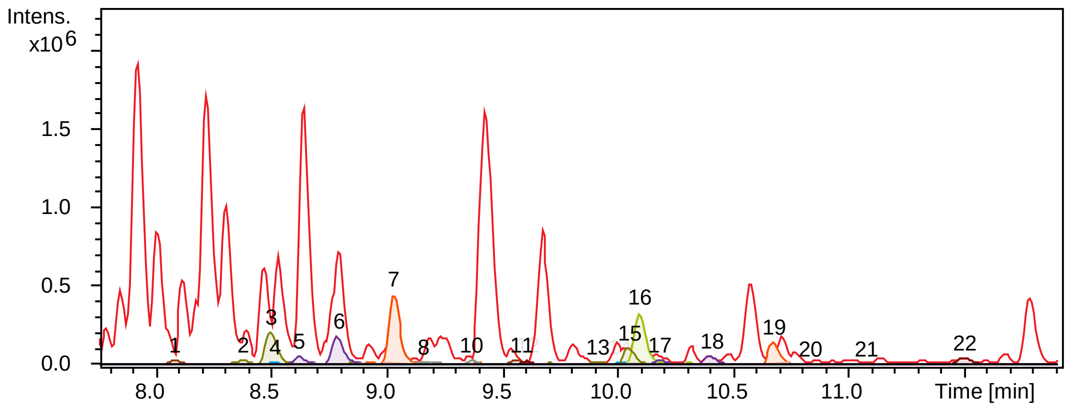

Figure R5|HPLC-ESI-HRMS analysis of the methanolic extract of *M. xanthus* DK1622 pMYC20Cys_v2. The BPC (red) and the EICs of all cystobactamids identified in the extract are shown. Peaks referring to cystobactamids are numbered.

The relative quantification shown here or the absolute quantification using a standard (e.g. Cys919-1) to quantify structurally different derivatives is, in our opinion, only useful to roughly estimate the quantity of cystobactamids. Previously, we decided to not include the relative quantification in the manuscript as it does not significantly contribute to the understanding of the cystobactamid biosynthesis. If the reviewers suggest to include those data, we are happy to do so.

Structures in SI Fig 7 are too small

Structures enlarged.

SI Fig 9: no bend triple bond in bottom molecule

Corrected.

Reviewer #3 (Remarks to the Author):

The present manuscript used both *in vivo* heterologous expression and *in vitro* reconstitution to study the unresolved key steps in cystobactamid antibiotic biosynthesis. The authors presented quite interesting findings, including the unique biosynthesis of diverse linkers connecting the two *para*aminobenzoic acid moieties, the shuttling system that incorporates these linkers into the assembly line, as well as skipping a few upstream modules to generate the truncated product. Especially, the authors obtained initial evidence to connect a novel AMDH domain to the bifunctions of isomerization and dehydration involved in the unique linker biosynthesis. Although the enzymatic and biochemical mechanism study of this bifunctional AMDH domain was beyond the scope of this manuscript, the authors showed some preliminary data in this manuscript to support the findings that which function the AMDH domain performs depends on its interaction with other domains/proteins of the pathway (e.g., CysJ which hydroxylates CysH-bound L-asparagine) and further affects the subsequent pathway function and thus the final product generation (e.g., CysQ catalyzed *O*-methylation only occurs in the presence of this AMDH domain). In addition, the authors demonstrated the establishment of a heterologous expression platform of the Cys pathway that allowed for genetic manipulation of this pathway, leading to new natural and unnatural Cys products as well as facilitating the biosynthetic study of this pathway. The authors provided detailed information on experimental methods and materials. The authors also performed a large set of experiments to generate substantial data supporting their hypothesis and findings. This manuscript revealed some quite interesting and unusual NRPS biosynthetic logics. The identification of the bifunctions of a previously unexplored but widely distributed AMDH domain opens up an opportunity to subsequently elucidate its unique mechanism and enzymology as well as to apply its enzymatic potential to synthetic biology and bioengineering. The heterologous expression methods and platform demonstrated in this manuscript can also be leveraged for expression and manipulation of other myxobacterial biosynthetic pathways. Overall, the novelty and significance of this manuscript validated through the experimental data justifies its suitableness for publication in *Nature Communications*. However, a revision is needed before its acceptance, as commented below.

The Introduction section was not well organized and was lengthy. The authors are suggested to rewrite this section in a logic and concise way leading to the theme and findings of the manuscript.

We have our problems to follow the argumentation of the reviewer and are not sure to which degree he expects us to change the organization of the introduction. Also no other reviewer suggested re-structuring this section. However, we agree that the transitions and the order between text sections can be improved. Thus, we suggest the following changes:

After the initial section describing the structure, activity and mode of action of cystobactamids and their relatedness to albicidin, we bring the section about NRPSs (generally describing their functionality and rules). Therefore, we suggest to remove the section about the establishment of a heterologous production platform and its usability for the elucidation of biosynthetic processes right after the section describing the previously proposed biosynthesis of cystobactamid and albicidin. The order would then be: Cystobactamid structure, activity, mode of action, relatedness to albicidin → general rules and functionality regarding NRPSs → previously proposed biosynthesis of cystobactamid and albicidin → problems to investigate biosynthesis/heterologous expression as potential solution → section about this work and the achieved results.

Figure 3f: *isomerization reaction was shown in a parenthesis* along with a figure caption "**f:** CysH incubated with L-asparagine and CysJ leads to the formation of β-hydroxy-L-asparagine *or α- hydroxy-L-isoasparagine*". In the meanwhile, the authors mentioned in the main text "because the expected *isomerization of L-asparagine cannot be observed by MS*". The authors are suggested to make these places clear and consistent. In addition, can the isomerization not be observed or not be distinguished by MS?

We were not able to confirm the isomerization from (β-hydroxy-)L-asparagine to (α-hydroxy-)L-isoasparagine using protein MS since the masses of both linkers are identical and their fragmentation patterns do not allow differentiation. However, the *in vivo* experiments shown on page 14-16, lines 226-245 (including Figure 5) and p. 17, lines 265269 clearly show that the isomerization occurs and is dependent on the presence of the AMDH domain.

As described above (reply to reviewer #1), we suggest changing the figure caption as follows:

f: CysH incubated with L-asparagine and CysJ leads to loading of the substrate onto CysH with subsequent hydroxylation by CysJ (see Figure 4). The isomerization of (β-hydroxy-)L-asparagine to (α-hydroxy-)L-isoasparagine is shown in parenthesis, because this step cannot be observed by MS. The isomerization was confirmed by deletion of the AMDH domain from the BGC and analysis of the production profile after heterologous expression of the respective construct in *M. xanthus* DK1622 (see Figure 5).

Since "the expected isomerization of L-asparagine cannot be observed by MS", the authors used targeted gene/domain deletions and subsequent heterologous expression of the manipulated pathway to probe the aminomutase function of the AMDH domain. However, the *in vivo* AMDH domain deletion from the entire pathway of the heterologous expression leaves a question about how to ensure the observed results was indeed caused by the loss of domain function, but not by domain

linker region disruption or disruption of protein-protein interactions between the AMDH domain and other possibly unidentified proteins that may contribute to the L-Asn isomerization? For example, the authors observed and mentioned that “We speculate that the abolishment of *O*-methylation in absence of the AMDH domain in CysH is linked to proteinprotein interaction between AMDH and CysQ”. Likewise, there might be some other such protein-protein interactions in the Cys pathway that might haven’t been realized yet. Therefore, did the authors make any efforts/attempts to ensure their observation was due to the loss of the domain function? For example, if the authors could perform some catalytic site point mutation experiments, instead of entire domain deletion, in their future follow-up study when the structure information of the AMDH or similar domains becomes available, the functions of the AMDH domain could be further strengthened.

As explained in the reply to reviewer #1 above, we are currently working on this. However, so far we were not able to clone the respective constructs and suggest that this experiment will be part of a second publication. Furthermore, we agree that this point has to be addressed in the manuscript. Therefore, we suggest to add the following sentence on p. 23, line 398:

“The mutation of conserved amino acid residues in the core motifs of the AMDH domain, e.g. in the ATP binding site, might inactivate only one of the two functions and does not pose the risk to disrupt protein-protein interactions as compared to deletion of the entire domain.”

A question related to Point 3 above: the AA residuals involved in the dehydration function of the AMDH domain is likely to be more predictable (e.g., ATP-binding site) compared to the aminomutase function, so have the authors tried any point mutation(s) to probe this and confirm this function of the AMDH domain?

As described in the reply to reviewer #1, we planned to test single point mutations in four different positions in the AMDH domain. Two of the independent mutations are located in the potential ATP-binding site. We hope to influence or completely abolish the dehydration function. Two further independent mutations change highly conserved cystein residues, which might be involved in metal binding (we observed a dark brown color for CysH indicating the presence of a metal). Maybe we are able to influence the aminomutase activity if the hypothetical metal-binding is affected.

The authors identified all the new Cys products only by HRMS and MS2 fragmentation due to the low yield of the products during the scaled-up cultivation. In their follow-up studies, perhaps the authors can try to optimize the cultivation conditions or genetically manipulate the pathway to enable compounds isolation and subsequent NMR verification of the novel derivatives. This will further

validate the function of the AMDH domain. But due to the timeline and the feasibility, this can be a separate project following this manuscript.

We already tried a few (< 5) different cultivation media and the supplementation of *p*ABA and L-asparagine; however, without improved production yields (data not shown). We agree that further optimization is required, especially when the cultivation volume was scaled-up which resulted in substantial decrease in the production yield. In the future, we aim for a robust fermentation process under constant and controlled cultivation conditions (e.g. pH, aeration, etc.) to isolate the novel derivatives and test their bioactivities, which may also allow to draw further conclusions about structure-activity-relationships.

Lines 158-160 of the main text: "Despite high structural similarity between these two unusual domains, completely different reaction mechanisms were proposed (Supplementary Figure 9b)": should it be both Supplementary Figures 9b and 9c?

Yes, corrected.

In Figure 3g: CysHDAMDH above the domain illustration: should it be CysHΔAMDH?

Yes, corrected.

In subfigures f and g of Figure 3: Asn should be changed to L-Asn for consistence.

Done.

Lines 198-200 of the main text: "Incubation of CysJ with free L-asparagine and α–KG with subsequent analysis using thin layer chromatography (TLC) indicated no hydroxylation of free Lasparagine (Supplementary Information)": should indicate where in the Supplementary Information. Is it Supplementary Figure 14?

Done.

Line 209 of the main text: should also cite Supplementary Figure 15 to show the unloading of LAsn using the cysteamine approach.

Done.

In Figure 5c: CysQ was crossed out by a red cross in the figure, which could be misleading as it seemed that CysQ was deleted, as opposed to the meaning that *O*-methylation by CysQ didn't occur.

Corrected.

Lines 81-82 of the main text: “such as shown for the daptomycins, the only natural product known featuring the unusual amino acid L-kynurenine”: some other natural products also feature L-kynurenine or modified L-kynurenine, such as taromycin.

We changed the sentence to: “[…] such as shown for the daptomycins, a natural product featuring the unusual amino acid L-kynurenine.”

In lines 277-279 of the main text, the authors concluded that “The product ratio of cystobactamid derivatives with different linkers is highly dependent on the reaction kinetics of the enzymes involved in linker biosynthesis”: this manuscript didn’t perform any kinetics studies and this conclusion was only the authors’ hypothesis. This sentence is thus suggested to be revised.

Following the suggestion of reviewer #1 to explain our hypothesis regarding the product ratio and reaction kinetics in more detail, we added the following text in the revised manuscript:

“We speculate that the isomerization of β-hydroxyl-L-asparagine by the AMDH domain is more efficient than the isomerization of L-asparagine, or that CysH, CysJ and CysQ form a protein complex influencing the reactivity of the AMDH domain. If CysJ is deleted, the isomerization reaction may occur much slower, thus leading to a decreased L-isoasparagine/L-asparagine linker ratio and an increased probability of L-asparagine dehydration.”

Furthermore, we changed the sentence that you have mentioned (changes shown in blue): “Furthermore, we speculated that the product ratio […]”

Lines 403-404 of the main text, “Furthermore, from our results we also draw conclusions about the biosynthesis of the albicidin linker”: “draw conclusions” was not used appropriately since the authors didn’t experimentally study this pathway at all in this manuscript. It was the authors’ speculation/deduction based on their current study on Cys pathway.

We deleted the entire paragraph as reviewer #4 suggested and wrote a different paragraph instead, because we had to include recently identified albicidin derivatives into the discussion (see below).

Supplementary Figure 9 b: what does that vertical line from domain B pointing to NH mean there?

The vertical line between B and NH should be a hydrogen bonding between a base (B) in the active center of the unknown domain (labelled as “?”) in AlbIV, which is homologous to the AMDH domain in CysH. We adapted it from the original figure from Cociancich *et al.*, in which also a continuous line was used as shown here (highlighted by red circle):

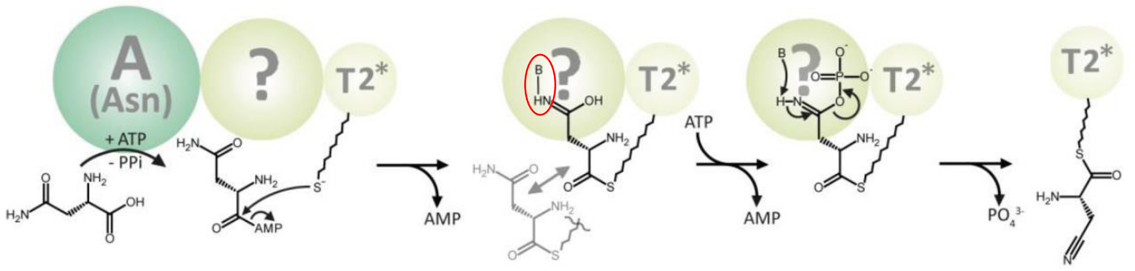

Admittedly, a dashed line would fit better to show the hydrogen bonding. We now modified Supplementary Figure 9 and decided to show a different version of the first two steps compared to the scheme from Cociancich *et al.*, because we think it is now clearer. Furthermore, we added a free electron pair below "B" to underline that B is a catalytic base in the "?" domain. Additionally, we added the stereochemistry to the structures and modified the proposed cystobactamid linker biosynthesis from Baumann *et al.* in Supplementary Figure 9c since it did not exactly show what was previously hypothesized. The modified Supplementary Figure 9b and c plus figure caption are shown below (Supplementary Figure 9a is skipped here):

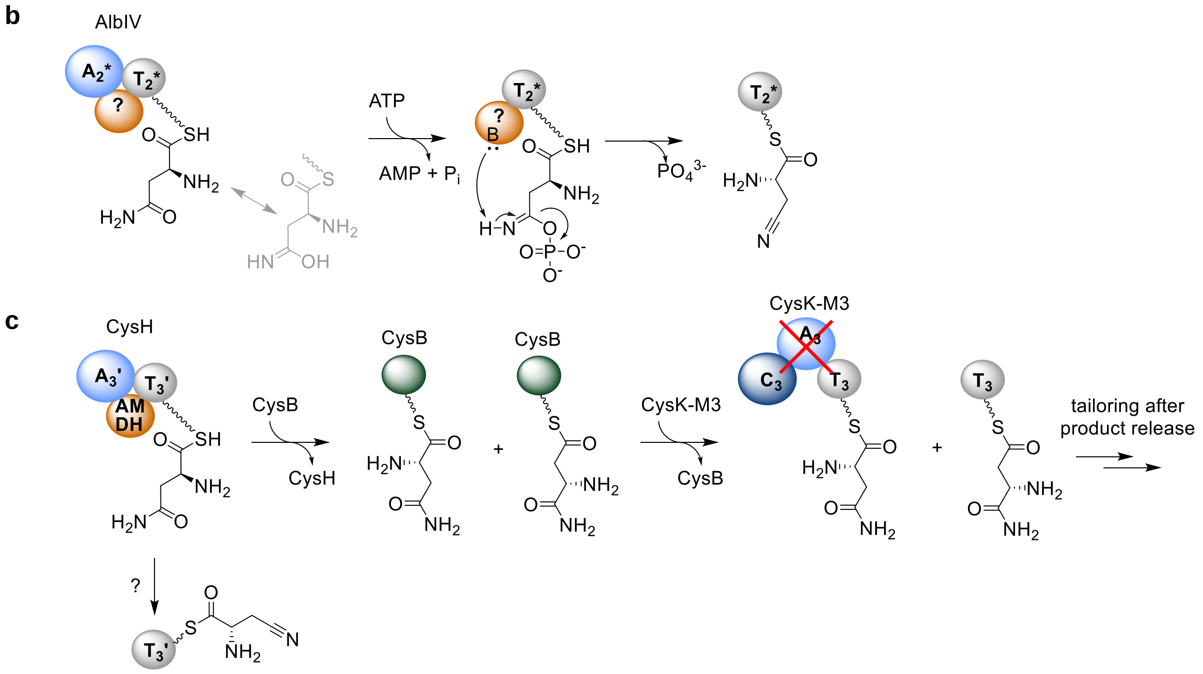

Supplementary Figure 9|Previously proposed linker biosynthesis pathways. b: Proposed reaction mechanism in albicidin linker biosynthesis for the generation of β-cyanoalanine (modified from [8]). **c:** Proposed cystobactamid linker biosynthesis for the generation of L-asparagine and L-isoasparagine. No biosynthesis pathway for β-cyanoalanine was proposed in cystobactamid linker biosynthesis so far.

In the main text when the authors cited the materials from the SI, they used different formats throughout the manuscript. For example, “Figure 4; Supporting Information” (line 209) v.s. “Supplementary Figure 4” (line 637) v.s. “Supplementary Information” (line 199): the format should be unified.

Corrected.

Reviewer #4 (Remarks to the Author):

The paper entitled « In vivo and in vitro reconstitution of unique key steps in cystobactamid antibiotic biosynthesis" by S. Gross et al depicts a quite impressive piece of work aiming, at the first place, at correcting the previously published biosynthetic genes cluster (BGC) involved in the biosynthesis of cystobactamids. Also described is the transfer of the whole BGC into a heterologous host using a state-of-the-art process. But the largest part of the paper allows the authors to propose updated detailed processes for the biosynthesis of the linker part of the molecules (with a special focus on the AMDH domain), as well as to describe the action of several genes involved in hydroxylations and O-methylations of the linker and of the pABA units. Finally, this long paper investigates the shuttling mechanism allowing the transfer of the linker part from a stand-alone NRPS module to the main assembly chain. Overall, the paper is very well written and one can read it in an easy and pleasant manner. Apart from a few exceptions listed below, extensive information is given, either in the main paper or in the supplementary information, regarding experimental conditions and results. Nevertheless, I have some major and minor remarks (see below) that I think, should the authors take them into account, would improve the actual manuscript.

Major Remark 1:

It is obvious that a manuscript centered on cystobactamids requires full comparison with their close neighbors from the albicidin family, considering the many similarities in the structures and in the biosynthetic schemes of the molecules from both families. Thus, several times throughout the paper, the authors compare their results with the corresponding findings in the albicidins family. However, the statements which concern albicidins are often incomplete, to such an extent that they appear almost wrong. I am wondering whether the authors have read the recent papers on albicidins (a small dozen since 2014). I would urge them to carefully read them, and to correct some of their statements regarding albicidins (see two examples below).

Example 1: page 3, legend Figure 1, last sentence (see also page 4, lines 63-64): these statements correspond to the beta-albicidin. However, at least nine other natural analogues have been identified and fully structurally described (see von Eckardstein et al, 2017, Chemistry, a European Journal 23, 15316-15321), some of which do not display beta-cyanoalanine as linker but rather beta-O-methyl-asparagine (two derivatives) or beta-O-methyl-cyanoalanine (two derivatives).

We thank the reviewer to bring this to our attention. We indeed missed to cite this obviously important reference about the recently published derivatives of albicidin.

We made the following changes in our manuscript (with changes in the text shown in blue):

p. 3, Figure 1:

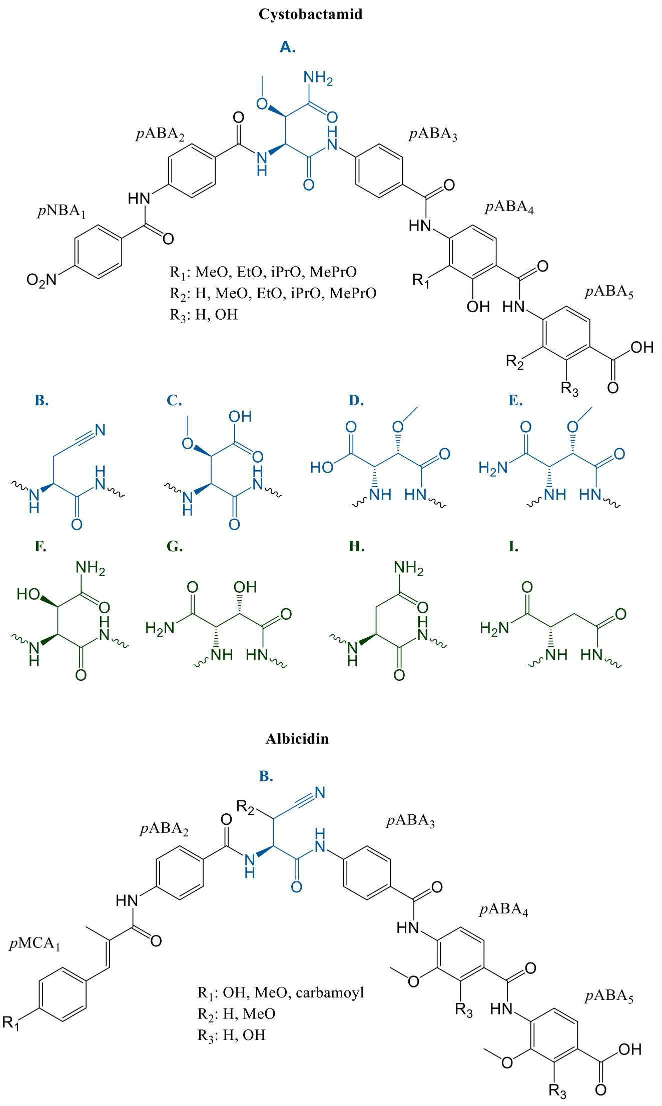

Figure 1|Structural variations among native and unnatural cystobactamids and structure comparison with albicidin. *para*-Nitrobenzoic acid (*p*NBA) and *para*-aminobenzoic acid (*p*ABA) with possible substitutions (R$_1$, R$_2$, R$_3$) are shown in black. Different linker moieties of natural cystobactamids (shown in blue; linker A-E) and unnatural cystobactamids (shown in green; linker F-I; Table 1): A: β-methoxy-L-asparagine, B: β-cyano-L-alanine, C: β-methoxy-L-aspartate, D: α-methoxy-L-isoaspartate, E: α-methoxy-L-isoasparagine, F: β-hydroxy-L-asparagine, G: α -hydroxy-L-isoasparagine, H: L-asparagine, I: L-isoasparagine. Scheme was adapted from Hüttel *et al*. Albicidin carries an N-terminal *para*-methylcoumaric acid (*p*MCA$_1$), two *p*ABAs, two substituted *p*ABAs and a (possibly modified) β-cyano-L-alanine (B) or β-methoxy-L-asparagine (A) linker. Different possible substitutions (R$_1$, R$_2$, R$_3$) are given.

p. 4, lines 60-64: "Notably, cystobactamids show structural similarity with albicidins (shown in Figure 1), a PKS/NRPS product class isolated from *Xanthomonas albilineans*, which also shows antibacterial activity.[8–10] However, significant structural differences between both compound classes are found in the N-terminal parts and the linker moieties. A methylated *para*-coumaric acid moiety (*p*MCA$_1$) typically forms the N-terminal part in albicidins, which can be further tailored e.g. with a carbamoyl group,[9] whereas native cystobactamids are restricted to *p*NBA$_1$. The linker moieties arising in albicidins

are β-cyano-L-alanine, which was also observed in Cys871,[2] and L-asparagine, both optionally methoxylated,[9] but no L-isoasparagine linker was described so far."

p. 6, lines 115-116: "With these results we were able to decipher most of the obscure and unique steps of cystobactamid ~~as well as provide a hypothesis for β-cyanoalanine~~ linker biosynthesis ~~in albicidin~~."

Example 2: page 24, lines 403-408: The authors speculate that, in the albicidin biosynthesis, the domain similar to AMDH "is exclusively dehydrating L-asparagine because no essential interaction partner (like CysJ in cystobactamid biosynthesis) catalyzes hydroxylation of the substrate prior to a potential isomerization". This is rather presumptuous since the cited paper from the Cociancich group describes only the first-ever isolated member of the albicidin family (namely the beta-albicidin). It appears that this molecule displays a beta-cyanoalanine as linker, and as such, the authors proposed the action of the AMDH-like domain to transform asparagine in beta-cyanoalanine. At no point, the authors wrote that the AMDH domain might not have any other function. Moreover, there is also, in the albicidin BGC, a homolog of CysJ (Alb08) which might as well play the role of the interaction partner. Also, I do not understand the last sentence of the same paragraph (page 24, lines 411-413): I do not see the point of expressing heterologously CysJ and CysQ in the native albicidin producer or the heterologous producer since both of them do possess the corresponding homologs (i.e. Alb08 and Alb06). As a conclusion for this example, this paragraph contains many incomplete or false statements, and this leads me to recommend that the authors either correct their statements or delete the whole paragraph.

According to the reviewers suggestion we deleted the whole paragraph and inserted a statement about the AMDH domain as follows:

"Interestingly, no albicidin derivative harboring an L-isoasparagine linker has been identified so far, indicating that the AMDH domain homolog in the albicidin biosynthesis is not able to perform an aminomutation-type reaction. Furthermore, von Eckardstein and coworkers described an albicidin derivative with a methoxylated β-cyano-L-alanine linker, a linker-type not found in cystobactamids. Based on our experiments, we hypothesized that β-hydroxylation of L-asparagine occurs much faster than the dehydration and that the β-hydroxylation prevents the AMDH domain from dehydrating the substrate. This explains why we only identified a considerable amount of Cys871 harboring a β-cyano-L-alanine linker after deletion of the hydroxylase CysJ. However, the existence of the methoxylated β-cyano-L-alanine linker in albicidin either means that AlbVIII (the homolog of CysJ) is able to hydroxylate β-cyano-L-alanine or that the AMDH domain homolog in AlbIV is able to dehydrate

β-hydroxy-L-asparagine (both of which was not observed in the cystobactamid biosynthesis) or that another enzyme than AlbVIII catalyzes the hydroxylation of β-cyano-L-alanine. Since Cys871 is only a very minor derivative in the presence of CysJ, one could also speculate that CysJ, likewise to AlbVIII, is able to hydroxylate β-cyano-L-alanine but the generated cystobactamid derivatives with methoxylated β-cyano-L-alanine linkers are only produced in such minor amounts that the ion intensity in MS does not exceed the detection limit. In this case the inactivation of the aminomutation function of the AMDH domain in CysH might lead to an increased production of Cys871 and the detection of methoxylated β-cyano-L-alanine linkers in cystobactamids. An alternative experiment would be the exchange of the AMDH domain in CysH by its homolog from AlbIV, which could potentially lead to a more pronounced dehydration reaction and subsequent hydroxylation by CysJ. In any case it needs further experimental investigation to understand the order and under which circumstances the respective linker modifications take place in the cystobactamid and albicidin biosyntheses."

The requested corrections in this major remark 1 will also require some additional references (at least von Eckardstein et al., 2017 which describe many of the natural analogues from the albicidin family). I am also wondering why the authors do not mention the coralmycins, which are also strongly resembling cystobactamids and albicidins, although there is less literature available about this family.

Additional albicidin reference was added.

We referenced a coralmycin paper on page 8, line 151 in our original manuscript. The identification of "coralmycin D" in our producer indicates that coralmycins are actually identical to cystobactamids, originating from a highly homologous biosynthesis pathway.

<u>Major remark 2:</u>

Page 10/11, Figure 2 and corresponding legend (Substrate specificity of CysH): I would have been interested in knowing the respective ratios of incorporation of all the tested substrates. The authors indicate a yes-or-no incorporation for a given substrate, but to which extent is a yes meaning? Is there any preferred substrate for CysH? The same question applies for other experiments (page 21, lines 352-354 and Suppl. Fig 11, as well as lines 366-369 and Suppl. Fig. 13).

Incorporation in this context indeed may have different meanings. We interpreted incorporation as "identified as inserted in the final product" whereas specificity of the A-domain can be measured separately but does not take into account any of the various downstream processes. We performed a

malachite green assay to detect phosphate liberation during the adenylation reaction of CysH as an indication for substrate specificity using different substrates as shown below:

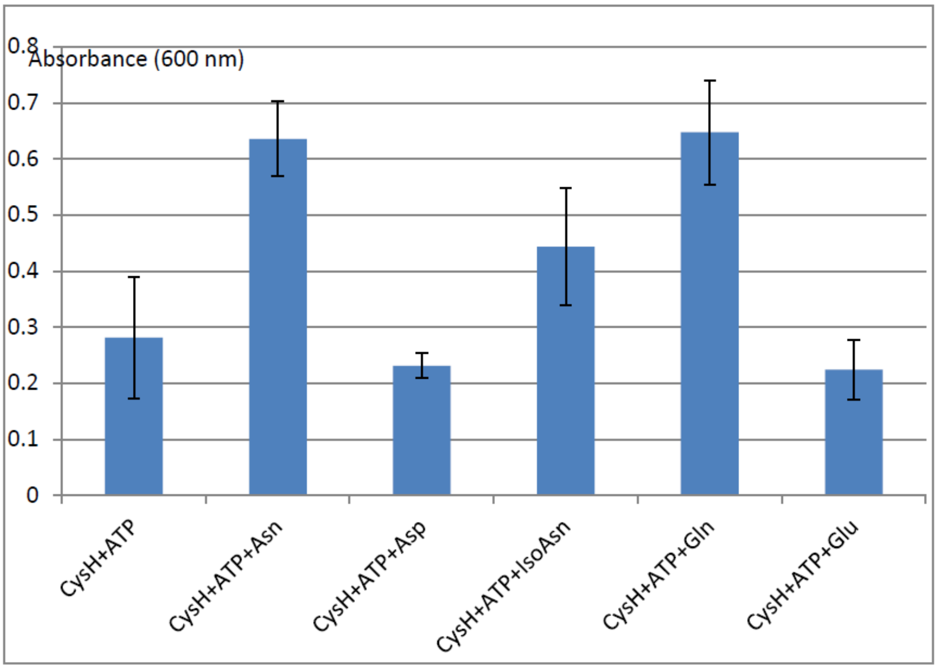

However, we only tested a few substrates, which made a concise statement about the preferred substrate of CysH difficult. The preferred substrates, based on this experiment, are L-asparagine and L-glutamine. However, since no cystobactamid derivative with a linker deriving from L-glutamine was detected so far, we hypothesize that the selectivity of downstream modules in the assembly line hinder its incorporation into cystobactamids. Similar findings have been reported for other biosynthetic systems where A-domains were shown to activate a variety of amino acids *in vitro* although many of these are not found in the final product. Furthermore, L-aspartate was not a valid substrate even though linkers C and D (see p. 3, Figure 1 in the manuscript) were found in some cystobactamid derivatives. Since we did not find a deaminase-encoding gene in the cystobactamid BGC, those linkers may derive from an externally encoded deaminase or from artificial deamination during the cultivation extraction process.

We did not perform comparable experiments for the remaining modules, because our focus lies on the biosynthesis of the linker moiety. In our opinion, the extent to which different substrates are adenylated was not significant enough to show in the manuscript and to gain a better understanding about the cystobactamid biosynthesis. However, we can include these data if the reviewer(s) feel that these experiments enhance the story we have presented.

Also, the authors state several times that cystobactamids exhibiting L-asparagine as linker display a more potent antibacterial activity. I would like to know which is/are the targeted bacteria.

The MICs of the following cystobactamids against certain bacteria were evaluated from Baumann *et al*. and Hüttel *et al*.:

Table S1. Minimum inhibitory concentrations (MIC in µg/ml) of cystobactamids **1-3**.[a]

Indicator strain	**1**	**2**	**3**	Cp[b]
Acinetobacter baumannii DSM-30008	> 59	7.4	32.5 - 65.0	0.2 - 0.4
Burkholderia cenocepacia DSM-16553	> 59	> 59	> 65	> 6.4
Chromobacterium violaceum DSM-30191	> 59	14.7	65.0	0.006 - 0.013
Escherichia coli DSM-1116	14.7 - 29.4	0.9	16.3	0.013
Escherichia coli DSM-12242[c]	29.4	0.9 - 1.8	32.5	0.05
Escherichia coli DSM-26863[d]	7.4	0.5 - 0.9	4.1 - 8.1	≤ 0.003
Escherichia coli ATCC-35218	14.7 - 29.4	0.9	16.3	0.013
Escherichia coli ATCC-25922	7.4	0.5	8.1	0.006
Enterobacter aerogenes DSM-30053	> 59	> 59	> 65	0.1 - 0.2
Klebsiella pneumoniae DSM-30104	> 59	> 59	> 65	0.025
Pseudomonas aeruginosa PA14	> 59	> 59	> 65	0.1
Pseudomonas aeruginosa ATCC-27853	> 59	> 59	> 65	0.1 - 0.2

Bacillus subtilis DSM-10	3.7	0.1	1.0 - 2.0	0.1
Enterococcus faecalis ATCC-29212	3.7 - 7.4	0.1	4.1	0.8
Micrococcus luteus DSM-1790	14.7	0.1 - 0.2	4.1	0.8 - 1.6
Mycobacterium smegmatis ATCC-700084	> 59	> 59	> 65	0.2 - 0.4
Staphylococcus aureus ATCC-29213	32.5	0.1	8.1	0.05 - 0.1
Streptococcus pneumoniae DSM-20566	14.7	0.1	4.1	0.8 - 1.6
Candida albicans DSM-1665	> 59	> 59	> 65	> 6.4
Wickerhamomyces anomalus DSM-6766	> 59	> 59	> 65	> 6.4

[a] MIC values have been determined in at least two independent experiments in a standard microbroth dilution assay and are defined as the lowest concentration of antibiotic that inhibited visible growth of the test organism. [b] Ciprofloxacin (Cp) was used as reference drug. [c] Nalidixic acid (Nal^{R}) resistant strain (> 60 µg/ml). [d] Strain with *tolC3* mutation.

Cys919-1 (**1**), Cys919-2 (**2**), Cys507 (**3**), Cp: Ciprofoxacin (this Table is shown in the Supporting Information of Baumann *et al.*)

and:

Table 2: Antibacterial activity of selected cystobactamids against a panel of Gram-negative bacteria. MIC values in µg mL^{-1} are given (n.d. = not determined).

	1b	1e	1f	1g	1i	1j	1k	1l	1m	CP[a]
Acinetobacter baumannii DSM-30008	8	0.5	16	> 64	64	> 64	> 64	> 64	2	0.8
Citrobacter freundii DSM-30039	1	0.06	8	2	8	> 64	> 64	> 64	1	0.003
Escherichia coli (WT)	0.5	0.13	16	2	4	> 64	> 64	> 64	0.5	0.013
E. coli (CP^{R})	2	0.25	n.d.	n.d.	n.d.	n.d.	n.d.	n.d.	n.d.	0.13
E. coli WT-3 [*gyrA* (S83L, D87G)]	64	0.5	16	> 64	> 64	> 64	> 64	> 64	2	0.8
E. coli (QnrD)	4	0.25	n.d.	n.d.	n.d.	n.d.	n.d.	n.d.	n.d.	0.1
E. coli (QnrS)	1	0.13	n.d.	n.d.	n.d.	n.d.	n.d.	n.d.	n.d.	> 6.4
E. coli WT-III[b] (*marR*Δ74bp)	> 64	0.5	32	> 64	> 64	> 64	> 64	> 64	2	0.1
E. coli DSM-26863 (*tolC3*)	0.25	0.06	n.d.	1	2	64	64	32	0.25	≤ 0.003
Enterobacter cloacae (QnrA)	> 64	1	n.d.	n.d.	n.d.	n.d.	n.d.	n.d.	n.d.	0.8
Klebsiella pneumoniae DSM-30104	> 64	> 64	n.d.	n.d.	n.d.	n.d.	n.d.	n.d.	n.d.	0.02
P. aeruginosa DSM-24600 (CRE)[b]	64	1	64	64	> 64	> 64	> 64	> 64	8	3.2
Proteus vulgaris DSM-2140	4	0.25	2	4	32	> 64	> 64	> 64	1	0.01

[a] Ciprofloxacin. [b] Carbapenem-resistant *Enterobacteriaceae*.

Cys919-2 (**1b**), Cys861-2 (**1e**), Cys871 (**1f**), Cys877-2 (**1g**), Cys905-2 (**1i**), Cys920-1 (**1j**), Cys920-2/coralmycin B (**1k**), Cys934-2 (**1l**), Cys935-2 (**1m**), CP: Ciprofloxacin (this Table is shown in the manuscript of Hüttel *et al.*).

Also, I think it would be of great importance to know the MIC or IC50 (or any other unit for antibacterial activity) for all the mutants of the cystobactamid BGC in the heterologous host (compared to the WT) (for instance, the CysQ, CysJ and AMDH mutants, respectively). This would complement the information gathered by mass spectrometry (see figure 5, for instance).

We agree that the isolation and structure elucidation as well as the MIC values of all new natural and unnatural cystobactamids is of great importance, but despite several attempts to purify sufficient amounts of those derivatives, we so far failed to do so. We are currently working on the production optimization in the heterologous host and as soon as we make improvements, we will try to isolate those derivatives. However, in the herein presented work, we focused on the biosynthesis, especially of the linker moiety, and the activity of the novel derivatives and potential structure-activity-relationships will be addressed in future works.

On this aspect, the authors ask (page 26, lines 450 and following) why Nature established a complex biosynthesis route (trans-acting AMDH and shuttling via CysB) to produce cystobactamids which are less biologically active. This question arises because they observe that, when targeting specific bacteria (and again, I would like to know which ones), cystobactamids with an asparagine as linker is more active than a cystobactamid with a beta-cyanoalanine for instance. I think that really depends on the target. Especially, the authors should remember that cystobactamids are naturally produced by a soil bacterium. Who knows which other bacteria (probably much different from the standard lab strains) they have to leave with, and eventually to fight using cystobactamids. I am convinced that Nature would not, by any means, keep such an energy-demanding complex process for nothing, and therefore I am sure that the answer lies in the natural environment of the cystobactamids-producing myxobacteria.

As shown above, in the microbial indicator strain panels tested by Baumann *et al*. and Hüttel *et al*., cystobactamids with L-asparagine linkers exhibited superior activity compared to the derivatives harboring L-isoasparagine linkers. The most active natural congener is Cys861-2, which is also the basis for improved synthetic derivatives (cited in the introduction, p. 4, lines 58-60). However, the reviewer addresses a critical point in his statement that the cystobactamids with L-isoasparagine linker might show activity against other bacterial strains from the natural habitat of the native cystobactamid producer strains.

Thus, we expanded this topic as follows (changes shown in blue):

"Notably, cystobactamids with L-asparagine linker showed superior antibacterial activity against numerous human pathogens like *A. baumanii*, *Citrobacter freundii*, *E. coli*, *Enterobacter cloacae*, *P. aeruginosa*, *Proteus vulgaris*, *Bacillus subtilis*, *Staphylococcus aureus* and *Streptococcus pneumoniae*.[1,2] The question arises why Nature established such a complex biosynthesis route including a *trans*-acting independent NRPS module with a shuttling process to produce cystobactamids which are biologically less active? It was previously shown[1,2] and confirmed in this study that naturally

a whole cocktail of cystobactamids is produced. Even though cystobactamids with L-asparagine linkers exhibited superior antibacterial activity against a panel of tested human pathogens, the natural producer strains have to outcompete a myriad of rival strains in their natural environment. It thus appears possible that the diversity of cystobactamids produced helps the natural producers to gain advantage over a variety of their competitors. Furthermore, it cannot be excluded that cystobactamids possess another function apart from their antibacterial activity, e.g. the involvement in developmental processes of the cell. However, from a human point of view, the simplest solution to produce more active cystobactamids with medicinal relevance harboring L-asparagine linkers would be the existence of an active L-asparagine-specific CysK-A_3 domain. Restoring the activity of the natively inactive A_3 domain by genetic engineering of the assembly line and thus bypassing the production-limiting shuttling process will be addressed in future experiments."

Minor remarks:

page 5, lines 74/75: some domains do indeed catalyze several reactions (dual condensation / epimerization domains, for instance).

We agree that this sentence is wrong and thank the reviewer for the hint. Since this sentence gives a very general statement and we do not want to complicate it unnecessarily, we decided to add only a "usually":

"Non-ribosomal peptide synthetases (NRPSs) are large enzyme complexes with a multimodular architecture, in which each module is subdivided into independent domains, each usually catalyzing a single reaction."

page 11, lines 179-181: This sentence is not clear. It must be either better explained or removed.

We agree that this sentence has to be explained better. We changed it as follows:

"All naturally occuring cystobactamids (except of the linker-free derivatives Cys449 and Cys507), which were identified thus far, harbor linker moieties deriving from L-asparagine. Since we also observed acceptance of other substrates by CysH, we assume that substrate specificities of downstream modules in the assembly line hinder incorporation of different amino acids than L-asparagine."

page 11, lines 181-186 and fig. 3c: The result depicted here is remarkably interesting (substrate dehydration observed after a prolonged incubation). But there are some discrepancies between what

is said here about the incubation time ("longer than 5 min") and what is mentioned elsewhere in the paper (page 38, lines 738-739, Materials and methods indicates 2h). Please make it clear.

We apologize for this mistake. We corrected it as follows:

"All reactions were incubated for either 5 min or 2 h at room temperature, respectively."

page 14, line 220: Add a space after "dicysteamine".

Done.

page 14, line 227: Add a point after "M"

Done.

page 17, line 265: "leads to a much less abundant product": How much less abundant?

The production of Cys919-1 was between 3-4 times higher compared to the production of Cys919-2 (based on the integrated peak surface areas after ESI-HRMS analysis) in the tested clones. After deletion of *cysJ*, the ratio between cystobactamid series 1 and series 2 (for Cys889-1a and Cys889-2a) was 0.46. This means that the isomerization reaction seems to be 6-8 times less efficient if CysJ is absent.

Based on the comments by reviewer #1, we already expanded the explanation. We suggest to add the following information (labelled in blue):

"Interestingly, isomerization of L-asparagine still occurs, but now leads to a much less abundant product. We speculate that the isomerization of β-hydroxyl-L-asparagine by the AMDH domain is more efficient than the isomerization of L-asparagine, or that CysH, CysJ and CysQ form a protein complex influencing the reactivity of the AMDH domain. If CysJ is deleted, the isomerization reaction may occur much slower, thus leading to a 6-8-fold decreased L-isoasparagine/L-asparagine linker ratio - compared to the α-methoxy-L-isoasparagine/β-methoxy-L-asparagine ratio in Cys919-1 and Cys919-2 - and an increased probability of L-asparagine dehydration."

page 23, line 378, last sentence of the figure legend: "Release of the final product by the TE domain". This is not depicted on fig 7, page 22.

For reasons of space and clarity, we did not change the figure, but we removed the sentence in the figure caption.

page 34, line 650: Replace “Supplementary Table 10 » by “Supplementary Table 11”

Done.

page 34, line 653: Write “M. xanthus” in italics

Done.

page 3, line 685: Replace “and 0.01 mg mL-1” by “and 0.1 mg mL-1”

Done.

Suppl. Info. page 8, paragraph “Synthesis of ...”, line 5: “and 50 µl DMF are added”: this step does not appear in Suppl. Fig 15c.

We added the missing step into the figure and found two more steps, which were not missing but inaccurate (changes shown in red circles):

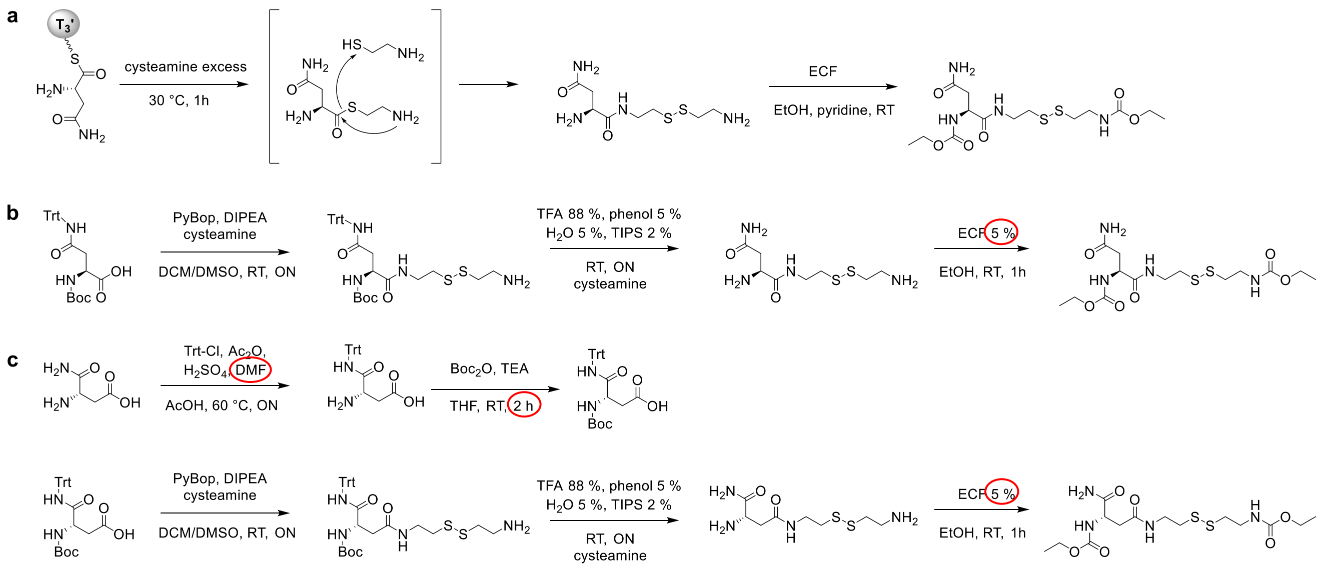

Suppl. Info. page 17, Suppl. Fig. 7: Drawings of the molecules are too small. One does not see the structural differences

Corrected.

Suppl. Info. page 18, legend Suppl. Fig. 8: Many errors lie in legend of Suppl. Fig. 8b:

We apologize for the numerous mistakes and thank the reviewer for the careful proof-reading.

* Remove "BPC and"

We removed the entire sentence.

* "EIC 890.29 (green)" does not appear in the figure

Removed.

* Add "(purple)" after "EIC 936.30"

Done.

* "show the existence of Cys919 1/2, Cys507, ..." does not correspond to part b of the figure. It is a copy and paste from part a. Please correct.

Corrected.

Suppl. Info. page 19, legend Suppl. Fig. 9: Replace "see Suppl. Fig. 12" by "see Suppl. Fig. 10"

Done.

Suppl. Info. page 40, Suppl. Table 10:

* line 2: Replace "see Suppl. Fig. 1" by "see Suppl. Fig. 6"

Done. We also added the sentence "Previously described derivatives are marked in grey." in the table description.

* Line 3: "see Suppl. Fig. 6" by "see Suppl. Fig. 8"

Done.

Reviewer #1 (Remarks to the Author):

All my comments were sufficiently addressed. I recommend the manuscript be accepted for publication in Nature Communications.

By the way, I noticed that R3 for Cys934-2 was missing "H" on Table 1. Authors are recommended to correct it.

Reviewer #3 (Remarks to the Author):

Reviewer 3: The authors have addressed my questions in the revised manuscript.

On behalf of Reviewer 2 per the editorial request: to the best of my knowledge, I believe that the authors have also addressed in the revised manuscript reviewer 2's original questions.

In summary, the revised manuscript is recommended for publication in Nature Communications.

Reviewer #4 (Remarks to the Author):

I thank the authors for the modifications taking into account most of my remarks, as well as those of other reviewers, bringing this paper to a high quality level. However, before I can suggest acceptation for the publication of this paper, a few (= six) points remain (some being minor) that should be cleared by the authors (see below; pages and lines numbering corresponds to the revised version of the paper):

* Page 26, line 434: I would replace "...the albicidin biosynthesis is not able to perform..." by "...the albicidin biosynthesis might not be able to perform...". Maybe albicidin derivatives harboring an L-isoasparagine linker do exist but they still have not been discovered yet (being below the threshold of detection for the techniques used so far, for instance).

* In the authors' response to my major remark 2, there is a paragraph starting with "We did not perform comparable experiments.....". In this paragraph, the authors state that they did focus on the biosynthesis of the linker moiety, and therefore did not consider significant enough to detail experiments about substrate specificity for the other modules. Still, the initial statements based on which I made my remark in the first round of review remain unchanged in the paper: at page 23 (lines 379-382 and lines 392-396), as well as in suppl. figs 11 & 13, the authors state results regarding specificity for A-domains substrates for modules other than the one responsible for the incorporation of the linker moiety. Therefore, they should, as proposed in their response to my remark, include the corresponding data in this paper.

* In the authors' response to my major remark 2, there is a paragraph starting with "We agree that the isolation and structure elucidation as well as the MIC values...". I fully understand the point made by the authors in this paragraph considering the lack of information about derivatives available in ultra-low quantities, but then, please, include this statement in the paper.

* Page 6, line 112: replace "9" by "nine".

* Suppl Info, page 8, paragraph "Identification of the new...", 7th and 8th lines: the sentence starting with "Fragment c..." lacks a verb.

* My last comment concerns a point that I did not see in the first round of review: it concerns the Suppl. Fig 16 (TLC analysis of CysJ activity on free L-asparagine): one can see that all lanes are not

taken from the same TLC plate, especially lane 3 which results obviously from a “copy and paste” operation. I would have preferred to see all the results on a single TLC plate. However, I leave it to the authors answer and to the editor’s decision regarding photo manipulation.

Once again, this paper is very impressive, and will be a landmark for the cystobactamids/albicidins/coralmycins community.

Authors response labelled in red. The page and line indications refer to the resubmitted manuscript.

Reviewer #1 (Remarks to the Author):
All my comments were sufficiently addressed. I recommend the manuscript be accepted for publication in Nature Communications.

By the way, I noticed that R3 for Cys934-2 was missing "H" on Table 1. Authors are recommended to correct it.

Corrected.

Reviewer #3 (Remarks to the Author):
Reviewer 3: The authors have addressed my questions in the revised manuscript.

On behalf of Reviewer 2 per the editorial request: to the best of my knowledge, I believe that the authors have also addressed in the revised manuscript reviewer 2's original questions.

In summary, the revised manuscript is recommended for publication in Nature Communications.

Reviewer #4 (Remarks to the Author):
I thank the authors for the modifications taking into account most of my remarks, as well as those of other reviewers, bringing this paper to a high quality level. However, before I can suggest acceptation for the publication of this paper, a few (= six) points remain (some being minor) that should be cleared by the authors (see below; pages and lines numbering corresponds to the revised version of the paper):

* Page 26, line 434: I would replace "...the albicidin biosynthesis is not able to perform..." by "...the albicidin biosynthesis might not be able to perform...". Maybe albicidin derivatives harboring an L-isoasparagine linker do exist but they still have not been discovered yet (being below the threshold of detection for the techniques used so far, for instance).

Corrected.

* In the authors' response to my major remark 2, there is a paragraph starting with "We did not perform comparable experiments.....". In this paragraph, the authors state that they did focus on the biosynthesis of the linker moiety, and therefore did not consider significant enough to detail experiments about substrate specificity for the other modules. Still, the initial statements based on which I made my remark in the first round of review remain unchanged in the paper: at page 23 (lines 379-382 and lines 392-396), as well as in suppl. figs 11 & 13, the authors state results regarding specificity for A-domains substrates for modules other than the one responsible for the incorporation of the linker moiety. Therefore, they should, as proposed in their response to my remark, include the corresponding data in this paper.

We do not agree with this point, because we made no statements about A domain specificities but only about the substrate tolerance of the entire modules. In our opinion the substrate tolerance of a module does not have to include the information to which extent a substrate is activated by the A domain, because the degree of substrate activation does not correlate with the extent of

incorporation into the final product (due to the selectivity of downstream modules). We think that a yes-or-no information (based on the presented intact protein LC-MS results) is sufficient to make a statement about the substrate tolerance of a module.

In the manuscript p. 26, lines 379-382 we stated: “Separate overexpression and purification of modules 1, 2 and 4 (CysK) with subsequent *in vitro* loading experiments revealed in the protein MS analyses that various *p*ABA derivatives are accepted” and p. 26, lines 393-395: “We separately overexpressed and purified modules 5 and 6 from *E. coli* BL21 and analyzed their substrate specificity *in vitro*.”

In case of our second statement, we changed the wording to “We separately overexpressed and purified modules 5 and 6 from *E. coli* BL21 and analyzed ~~their substrate specificity which~~ which substrates are processed *in vitro*.” to avoid that “substrate specificity” is unintentionally interpreted as A domain specificity.

The malachite green assay (monitoring phosphate release during amino acid activation by the A domain) was only performed in an early experiment phase in case of CysH to get a quick idea which substrates are tolerated by the A domain (before we established the intact protein LC-MS measurements). However and most importantly the malachite green assay was **not** performed for modules 1, 2, 4, 5 and 6. Our suggestion that “we can include these data if the reviewer(s) feel that these experiments enhance the story we have presented.” was related to the malachite green assay, which was **only performed for CysH**. We are sorry, if our phrasing was misleading. Furthermore, in our opinion it does not make sense to perform additional malachite green assays for the remaining modules because this will not change the storyline of the manuscript and will neither contribute in any other way to the understanding of the biosynthesis.

Based on our arguments presented in the first point-to-point response and here, we did not include the malachite green assay result of the CysH A domain. If the reviewer or editor is still of the opinion that we should include this result, we are willing to do so.

* In the authors’ response to my major remark 2, there is a paragraph starting with “We agree that the isolation and structure elucidation as well as the MIC values...”. I fully understand the point made by the authors in this paragraph considering the lack of information about derivatives available in ultra-low quantities, but then, please, include this statement in the paper.

On p. 28, lines 488-490 we added the text highlighted in blue: “However, the establishment of a robust fermentation process combined with media optimization has to be addressed in future experiments to enable purification, ~~and~~ NMR verification and determination of the antibacterial activity of the novel cystobactamid derivatives from this study.”

* Page 6, line 112: replace “9” by “nine”.
Corrected.

* Suppl Info, page 8, paragraph “Identification of the new...”, 7th and 8th lines: the sentence starting with “Fragment c...” lacks a verb.

We added “contains” to the sentence.

* My last comment concerns a point that I did not see in the first round of review: it concerns the Suppl. Fig 16 (TLC analysis of CysJ activity on free L-asparagine): one can see that all lanes are not taken from the same TLC plate, especially lane 3 which results obviously from a “copy and paste”

operation. I would have preferred to see all the results on a single TLC plate. However, I leave it to the authors answer and to the editor's decision regarding photo manipulation.
Yes, this figure is indeed built from more than one picture. This experiment was performed in the very beginning of our investigations and we also tested if CysJ acts on *p*ABA and other potential precursors in cystobactamid biosynthesis. As CysJ was later shown to be involved in the linker hydroxylation, we wanted to reduce the complexity of the picture and only show the lanes that are related to linker biosynthesis and are really necessary to understand that CysJ does not have activity on free L-Asn. We added a short sentence to the figure description to avoid any speculation on photo manipulation: "This figure was built from different TLC plates and depicts only relevant lanes showing that CysJ has no activity on free L-Asn."

Once again, this paper is very impressive, and will be a landmark for the cystobactamids/albicidins/coralmycins community.

We thank all four Reviewers for the fruitful discussion and for the help to improve our manuscript to a higher quality level!
